# Verbenalinosides A and B, Two Iridoid–Phenylethanoid Glycoside Conjugates from *Verbena officinalis* and Their Hepatoprotective Activity

**DOI:** 10.1021/acsomega.4c11149

**Published:** 2025-02-27

**Authors:** Xiao-Mei Liu, Wen-Jing Ren, Hao-Yuan Lyu, Cheng-Yu Chen, Xiao-Hui Pan, Li-Ping Bai, Wei Zhang, Zhi-Hong Jiang, Guo-Yuan Zhu

**Affiliations:** † State Key Laboratory of Quality Research in Chinese Medicine & Faculty of Chinese Medicine, Macau University of Science and Technology, Macau 999078, People’s Republic of China; ‡ State Key Laboratory of Quality Research in Chinese Medicine, Macau Institute for Applied Research in Medicine and Health, Macau University of Science and Technology, Macau 999078, People’s Republic of China; § Zhuhai MUST Science and Technology Research Institute, Macau University of Science and Technology, Zhuhai 519000, People’s Republic of China; ∥ National Engineering Research Center for Modernization of Traditional Chinese Medicine, Grand TCM Product-Cultivating Branch, Jiaheng (Zhuhai Hengqin) Pharmaceutical Technology Co., Ltd., Zhuhai 519000, People’s Republic of China

## Abstract

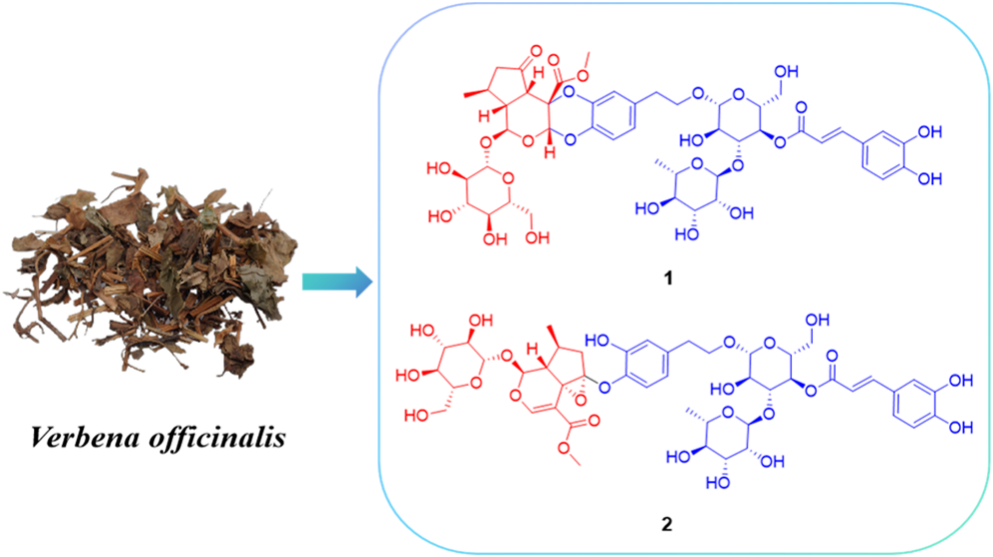

Two novel iridoid–phenylethanoid glycoside heterodimers, verbenalinoside A (**1**) and B (**2**), along with two known precursors, verbenalin (**3**) and verbascoside (**4**) were isolated from the dry aerial parts of *Verbena officinalis*. Verbenalinoside A (**1**) possesses a new 5/6/6/6 core fused by the double bond of iridoid and phenolic hydroxyl groups of the phenylethanoid unit. Verbenalinoside B (**2**) is a conjugate of verbenalin (**3**) and verbascoside (**4**) by an ether bond. The structures of these compounds were elucidated through the comprehensive analysis of spectroscopic data, supported by electronic circular dichroism (ECD) calculations and DP4 plus NMR calculations. Biological assay results showed that **1**–**4** can concentration-dependent rescue the ethanol-induced hepatotoxicity in LO2 and HepG2 cells, indicating that **1**–**4** should be potential hepatoprotective constituents of *V. officinalis*.

## Introduction

**Figure 1:**
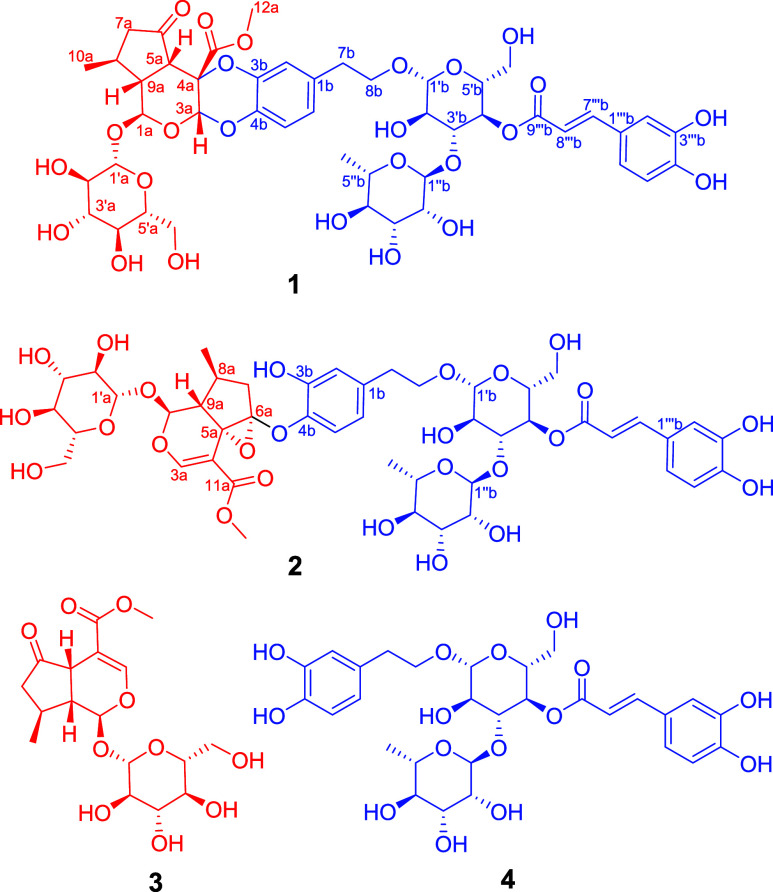
Structures of compounds **1**–**4**.

*Verbena officinalis* L. (Verbenaceae family), a perennial herb, is widely distributed across tropical and subtropical regions globally, with a predominant presence in central and southwestern China.^1,2^ It has been widely employed in traditional healing practices across Europe, Asia, and North America, with a well-documented history of use spanning centuries.^3^ In China, *Verbena* herb is primarily used to treat abdominal masses, dysmenorrhea, amenorrhea, throat swelling, abscesses, edema, jaundice, and malaria.^4^ Previous phytochemical investigations and biological studies on *V. officinalis* resulted in the isolation and characterization of iridoid glycosides,^5^ phenylethanoid glycosides,^6^ flavonoids,^7^ phenolic acids,^8^ and terpenoids,^9^ which displayed various biological effects including antitumor, antioxidant, antibacterial, and anti-inflammatory effects.^10−13^ Notably, *V. officinalis* has been extensively documented in ethnopharmacological studies as a traditional therapeutic agent for hepatic infectious diseases.^14^ However, there is limited research on the chemical constituents and hepatoprotective activities of *V. officinalis*. At present, only two characteristic iridoid glycosides, verbenalin and hastatoside, have been reported to have anti-HCV, antialcohol-associated steatohepatitis, and antiliver fibrosis activities.^15−17^ Additionally, our preliminary pharmacological data revealed that the extract of *V. officinalis* has a protective effect against alcohol-induced liver injury. To discover more novel compounds with hepatoprotective activities from *V. officinalis*, the chemical constituents of the aerial parts of *V. officinalis* have been investigated in this study. Guided by liquid chromatography–mass spectrometry (LC–MS), two undescribed compounds (**1** and **2**) and two known precursors (**3** and **4**) were isolated from the aerial parts of *V. officinalis* (Figure 1), which demonstrated the hepatoprotective effects against ethanol-induced normal hepatocyte cells (LO2) and human hepatoma cells (HepG2).

## Results and Discussion

The 80% EtOH extract of the aerial parts of *V. officinalis* (10 kg) was partitioned with PE, EtOAc, and *n*-BuOH. The EtOAc and *n*-BuOH parts were then separated by the silica gel column chromatography, Medium-pressure liquid chromatography (MPLC), and HPLC to yield two undescribed compounds (**1** and **2**) and two known glucosides (**3** and **4**). Known compounds **3** and **4** were identified as verbenalin (**3**)^18^ and verbascoside (**4**)^19^ by comparing their NMR (Supporting Information, Tables S1 and S2) and high-resolution mass spectrometry (HRMS) data to those in the literature.

**Figure 2:**
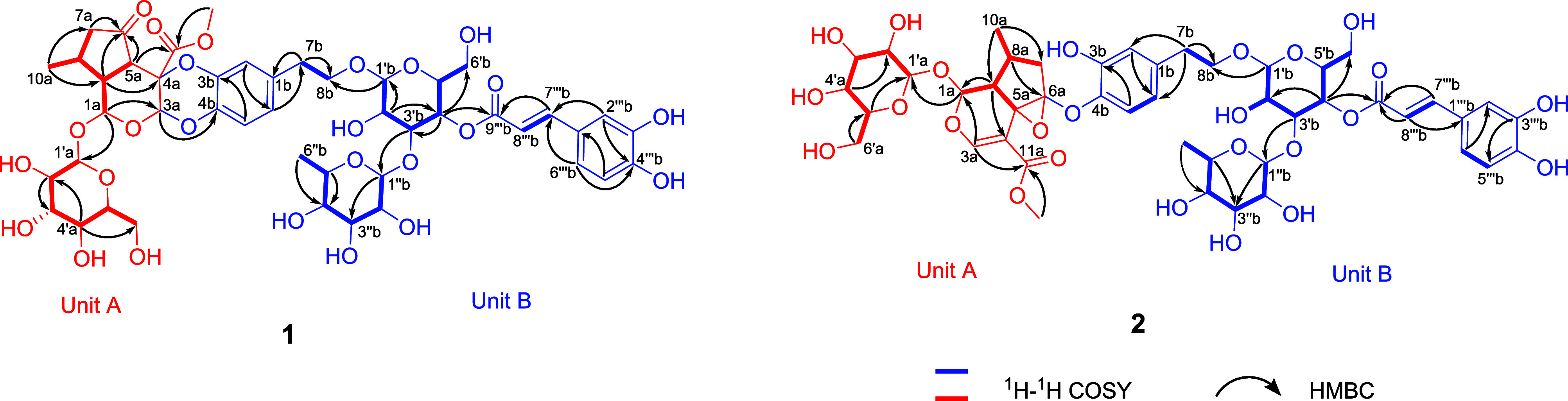
Key ^1^H–^1^H COSY and HMBC correlations of compounds **1** and **2**.

Compound **1** was obtained as a yellow powder with a molecular formula of C_46_H_58_O_25_, determined by its HRESIMS ion at *m*/*z* 1028.3611 [M + NH_4_]^+^ (calculated for C_46_H_62_NO_25_, 1028.3605), corresponding to 18 degrees of unsaturation. Its IR spectrum displayed characteristic absorptions of hydroxy, carbonyl, and olefinic functional groups at 3333, 1713, 1605, and 1042 cm^–1^. One-dimensional (1D) NMR (Table 1) and HSQC spectra identified the signals for one carbonyl group (δ_C_ 216.3), two ester carbonyls (δ_C_ 170.9 and 168.3), two ABX aromatic rings [δ_C_ 123.7, 118.1, 118.0, δ_H_ 6.81 (d, *J* = 2.2 Hz), 6.78 (dd, *J* = 8.2, 2.2 Hz), 6.75 (d, *J* = 8.2 Hz); and δ_C_ 123.2, 116.6, 115.3, δ_H_ 7.06 (d, *J* = 2.2 Hz), 6.95 (dd, *J* = 8.2, 2.2 Hz), 6.77 (d, *J* = 8.2 Hz)], one *trans*-olefinic bond [δ_C_ 148.0 and 114.7, δ_H_ 7.59 (d, *J* = 15.8 Hz) and 6.27 (d, *J* = 15.8 Hz)], and three terminal carbon signals of sugars [δ_C_ 104.2, 103.1, and 99.2, δ_H_ 5.19 (1H, d, *J* = 1.8 Hz), 4.75 (1H, d, *J* = 8.0 Hz), and 4.38 (1H, d, *J* = 8.0 Hz)]. Compared the NMR data (Table 1) to those of verbenalin (**3**) and verbascoside (**4**), the main constituents in *V. officinalis*,^18,19^ implied that **1** is a heterodimer consisting of an iridoid glycoside (Figure 2, unit A in red) and a phenylethanoid glycoside (Figure 2, unit B in blue). In unit A, the double bond of verbenalin (**3**) is replaced by an oxygenated methine at C-3a [δ_C_ 88.4, δ_H_ 5.98, s] and an oxygenated quaternary carbon at C-4a [δ_C_ 76.9] in **1**. The NMR data for unit B of **1** are close to those of verbascoside (**4**), except for upshifts of C-3b (3.0 ppm) and C-4b (4.1 ppm) in **1**. The HMBC correlation from H-3a (δ_H_ 5.98, s) to C-4b (δ_C_ 139.2) and the MS data of **1** indicated that units A and B are connected to form a 1,4-dioxane ring through two ether bonds (C-3a–O–C-4b and C-4a–O–C-3b). A detailed 2D NMR data analysis (Figure 2) supported that compound **1** is a conjugate of **3** and **4**, which possesses a new 5/6/6/6 core.

**Table 1:** ^1^H NMR (600 MHz) and ^13^C NMR (150 MHz) Data for **1** and **2** (δ in ppm, CD_3_OD)

	**1**		**2**	
no.	δ_C_, type	δ_H_ (*J* in Hz)	δ_C_, type	δ_H_ (*J* in Hz)
iridoids				
1a	97.0, CH	5.60, br.s	101.3, CH	6.06, d (9.6)
3a	88.4, CH	5.98, s	160.7, CH	7.63, s
4a	76.9, C		109.0, C	
5a	51.8, CH	3.38, d, 8.7	79.0, C	
6a	216.3, C		103.8, C	
7a	48.0, CH_2_	1.97, dd (18.9, 10.6)	46.7, CH_2_	1.77, dd (14.0, 4.8)
		2.64, dd (18.9, 8.0)		2.39, dd (14.0, 4.8)
8a	33.8, CH	2.53, m	30.1, CH	2.31, m
9a	46.2, CH	2.11, dd (12.1, 8.7)	53.5, CH	2.33, d (9.6)
10a	19.1, CH_3_	1.17, d (6.4)	21.1, CH_3_	1.17, d (6.1)
11a	170.9, C		168.1, C	
12a	53.6, CH_3_	3.73, s	52.0, CH_3_	3.51, s
1′a	99.2, CH	4.75, d (8.0)	100.8, CH	4.82, d (7.9)
2′a	74.8, CH	3.26, m	75.0, CH	3.26, t (8.8)
3′a	78.2, CH	3.42, m	78.1, CH	3.43, t (9.0)
4′a	71.6, CH	3.32, overlap	71.6, CH	3.33, overlap
5′a	78.4, CH	3.32, overlap	78.5, CH	3.36, overlap
6′a	62.7, CH_2_	3.67, dd (11.4, 4.8)	62.8, CH_2_	3.71, dd (12.0, 5.7)
		3.88, d (11.4)		3.92, overlap
phenylethanoid glycoside				
1b	135.1, C		134.1, C	
2b	118.0, CH	6.81, d (2.2)	118.0, CH	6.75, d (1.9)
3b	142.4, C		143.7, C	
4b	139.2, C		140.3, C	
5b	118.1, CH	6.75, d (8.2)	117.8, CH	6.71, d (8.2)
6b	123.7, CH	6.78, dd (8.2, 2.2)	122.6, CH	6.68, dd (8.2, 1.9)
7b	36.5, CH_2_	2.86, m	36.5, CH_2_	2.84, t (7.2)
8b	71.7, CH_2_	3.76, m	71.9, CH_2_	3.75, dt (9.8, 7.2)
		4.10, dt (9.6, 7.2)		4.07, dt (9.8, 7.2)
1′b	104.2, CH	4.38, d (8.0)	104.2, CH	4.38, d (7.9)
2′b	76.2, CH	3.38, overlap	76.2, CH	3.39, overlap
3′b	81.7, CH	3.80, t (9.1)	81.7, CH	3.81, t (9.2)
4′b	70.7, CH	4.92, overlap	70.6, CH	4.92, overlap
5′b	76.1, CH	3.53, m	76.1, CH	3.54, overlap
6′b	62.4, CH_2_	3.51, m	62.4, CH_2_	3.53, overlap
		3.62, d (10.2)		3.61, m
1″b	103.1, CH	5.19, d (1.8)	103.1, CH	5.19, d (1.8)
2″b	72.4, CH	3.92, m	72.4, CH	3.92, overlap
3″b	72.1, CH	3.58, overlap	72.1, CH	3.58, overlap
4″b	73.8, CH	3.29, m	73.8, CH	3.28, t (8.9)
5″b	70.5, CH	3.56, overlap	70.5, CH	3.56, overlap
6″b	18.5, CH_3_	1.09, d (6.2)	18.5, CH_3_	1.09, d (6.2)
1″′b	127.7, C		127.7, C	
2″′b	115.3, CH	7.06, d (2.2)	115.3, CH	7.06, d (2.1)
3″′b	146.9, C		146.9, C	
4″′b	149.8, C		149.8, C	
5″′b	116.6, CH	6.77, d (8.2)	116.6, CH	6.78, d (8.2)
6″′b	123.2, CH	6.95, dd (8.2, 2.2)	123.3, CH	6.95, dd (8.2, 2.1)
7″′b	148.0, CH	7.59, d (15.8)	148.1, CH	7.59, d (15.8)
8″′b	114.7, CH	6.27, d (15.8)	114.7, CH	6.28, d (15.8)
9″′b	168.3, C		168.3, C	

**Figure 3:**
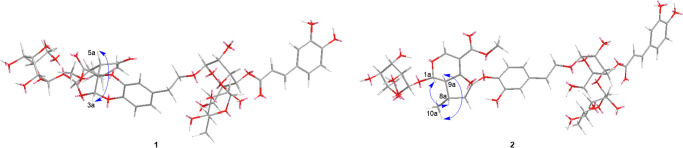
Key NOESY correlations of **1** and **2**.

**Figure 4:**
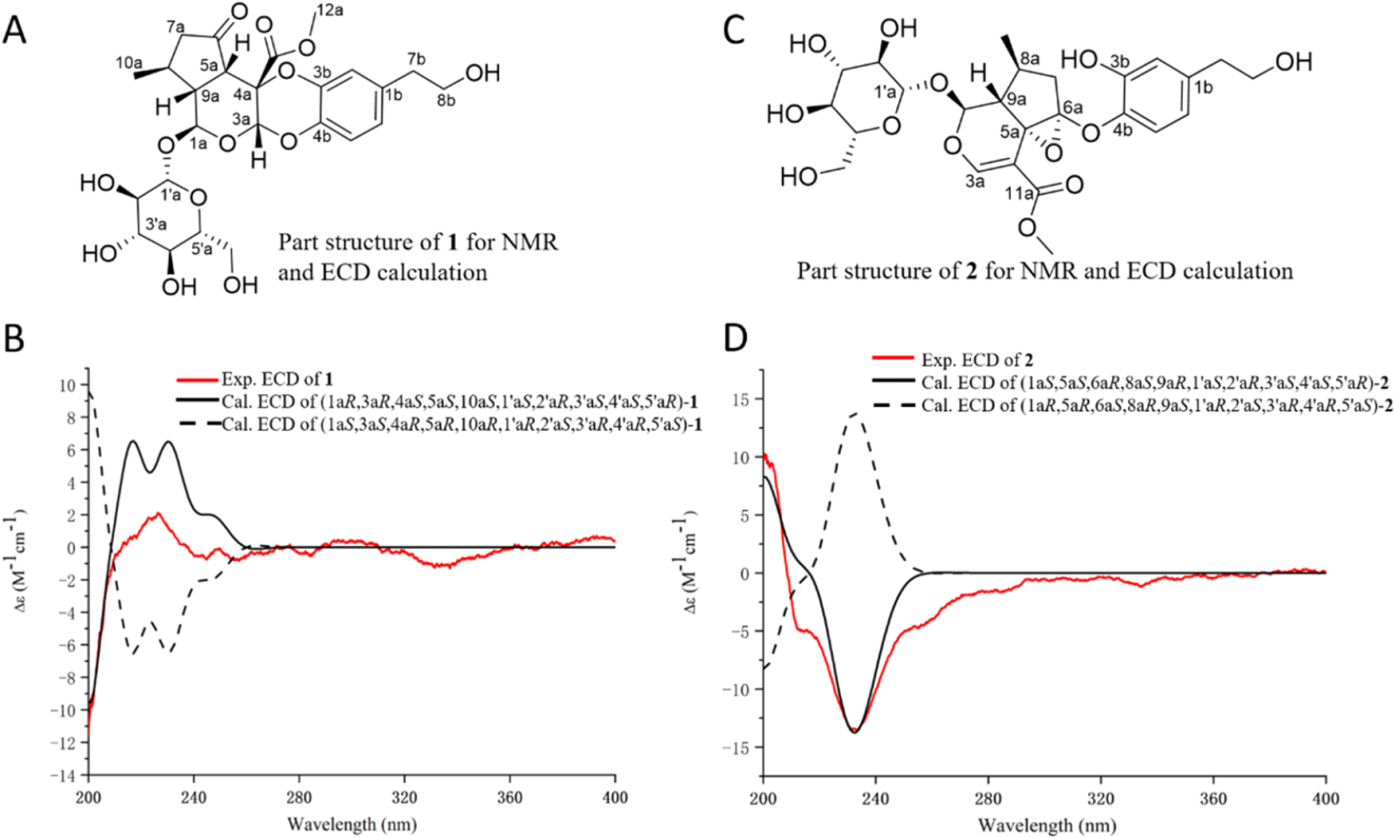
Experimental and calculated ECD data for **1** and **2**. (A) The structure part of **1** for NMR and ECD calculation. (B) The **e**xperimental and calculated ECD spectra for **1**. (C) The structure part of **2** for NMR and ECD calculation. (D) The **e**xperimental and calculated ECD spectra for **2**.

Considering the biogenetic origin of **1**, the relative configuration of **1** was inferred by analyzing its 1D NMR data and NOESY spectrum to be the same as those of its precursors, verbenalin (**3**) and verbascoside (**4**), except for two new chiral carbons (C-3a and C-4a). The NOESY signals between H-3a and H-5a suggested that these two protons are β-oriented. Unfortunately, the configuration of the quaternary carbon at C-4a cannot be assigned by NOESY correlations (Figure 3). The QM-NMR calculation was then employed to determine the configuration C-3a and C-4a. The DP4+ analysis was conducted on the calculated ^13^C chemical shifts of C-4a isomers of **1** (To calculate more accurately, the part structure of **1** including unit A and the phenylethanoid moiety was used. Figure 4A). The calculation results confidently suggested the calculated NMR data for 4a*S* of **1** with a 100% probability and an impressive correlation coefficient (*R*^2^) of 0.9972 (Supporting Information, Figure S24). Furthermore, the ECD spectra of (1a*R*,3a*R*,4a*S*,5a*S*,10a*S*,1’a*S*,2’a*R*,3′a*S*,4’a*S*,5′a*R*)-**1** and (1a*S*,3a*S*,4a*R*,5a*R*,10a*R*,1’a*R*,2’a*S*,3′a*R*,4’a*R*,5′a*S*)-**1** (Figure 4B) were predicted using the time-dependent density-functional theory (TD-DFT) simulations at the PBE0/def2-TZVP level in methanol solvent. The results indicated that the experimental ECD spectrum of **1** matched well with that of (1a*R*,3a*R*,4a*S*,5a*S*,10a*S*,1’a*S*,2’a*R*,3′a*S*,4’a*S*,5′a*R*)-**1** (Figure 4B), which further confirmed the configuration of **1**. Therefore, the structure of **1** was determined as depicted and designated as verbenalinoside A.

Verbenalinoside B (**2**) exhibited a quasi-molecular ion at *m*/*z* 1028.3605 [M + NH_4_]^+^ in its HRESIMS, consistent with the molecular formula C_46_H_58_O_25_. The ^1^H and ^13^C NMR spectroscopic data of **2** (Table 1) closely resembled those of **1**, both possessing the same iridoid glycoside (verbenalin, unit A) and phenylethanoid glycoside (verbascoside, unit B), except for a change in the connection position between units A and B. Compared with verbenalin (**3**), two oxygenated quaternary carbons at C-5a and C-6a (δ_C_ 79.0 and 103.8) in unit A of **2** were observed, which indicated that the methine (C-5) and ketone (C-6) groups of verbenalin (**3**) were changed in **2**. These results together with the HRMS data of **2** suggested that an additional ternary oxygen ring was formed between C-5a and C-6a. The chemical shift of C-6a (δ_C_ 103.8) in unit A and the upfield shift of C-4b (δ_C_ 140.3) in unit B combined with the HRMS data indicated that they are conjugated by an ether bond (C-6a–O–C-4b). The planar structure of **2** was fully confirmed through an additional 2D NMR analysis (Figure 2). The NOESY interactions (Figure 3) revealed that protons H-1a and H-8a are β-oriented, whereas the correlations between H-9a and H-10a indicated a α-orientation. These results confirmed that the configurations of C-1a, C-8a, and C-9a in **2** are the same as those of verbenalin (**3**). To establish the configurations of new chiral C-5a and C-6a in **2**, a theoretical calculation of ^13^C NMR chemical shifts for two isomers (Supporting Information, Figure S26) was conducted. The DP4+ analysis suggested configurations of 5a*S*, 6a*R* with 100% probability and a high linear correlation coefficient (*R*^2^ = 0.9908, Supporting Information, Figure S27). Additionally, the configuration of compound **2** was determined as (1a*S*,5a*S*,6a*R*,8a*S*,9a*R*,1’a*S*,2’a*R*,3′a*S*,4’a*S*,5′a*R*) through the comparison of its experimental and calculated ECD spectra (Figure 4C,4D). With these data as a foundation, the structure of verbenalinoside B (**2**) was established as presented.

**Figure 5:**
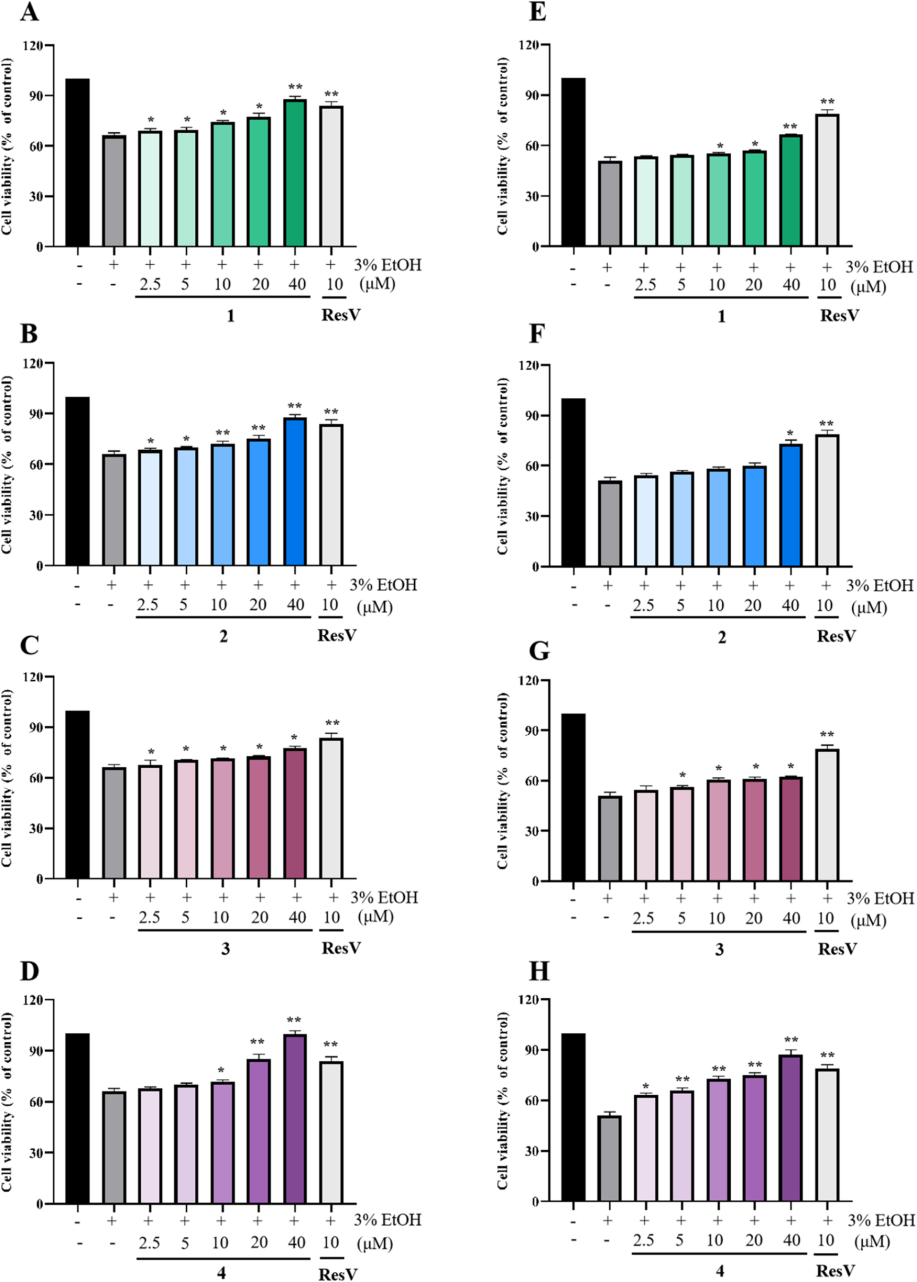
Hepatoprotective activities of **1**–**4** in LO2 cells (A–D) and HepG2 cells (E–H). All the data are expressed as mean ± SD from three individual experiments. ** *P* < 0.01 and * *P* < 0.05 indicate significant differences compared to the 3% EtOH group.

It has been reported that verbenalin (**3**) can alleviate hepatic damage in alcohol-associated steatohepatitis.^16^ However, the effect of verbenalin (**3**) and other constituents from *V. officinalis* on acute alcoholic liver injury is unclear. In this study, the hepatoprotective effects of compounds **1**–**4** on ethanol-induced injury were evaluated in LO2 and HepG2 cells.^20^ The MTT results showed that compounds **1**–**4** did not have remarkable cytotoxicity against LO2 and HepG2 cells at a high concentration of 40 μM. As shown in Figure 5, treatment with different concentrations (2.5–40 μM) of compounds **1**–**4** significantly improved the cell survival of the ethanol-induced LO2 and HepG2 cells in a dose-dependent manner. In alcohol-induced LO2 cell models, 40 μM of **1**–**4** treatment showed cell viabilities of 77.6, 99.7, 87.9, and 88.0%, respectively, comparing 65.7% of the alcohol-induced group, while exhibited cell viabilities of 66.5, 73.1, 62.2, 87.1% comparing 50.7% of the alcohol-induced group in HepG2 cell models. These results suggested that **1**–**4** have hepatoprotective activity in alcohol-induced liver cell damage.

## Conclusions

In summary, two new iridoid–phenylethanoid glycoside conjugates, verbenalinoside A (**1**) and B (**2**), and their precursors, verbenalin (**3**) and verbascoside (**4**) were isolated from *V. officinalis*. Verbenalinoside A (**1**) possesses a new 5/6/6/6 core fused by the double bond of iridoid with phenolic hydroxyl groups of the phenylethanoid unit. Verbenalinoside B (**2**) is a conjugate of verbenalin (**3**) and verbascoside (**4**) by an ether bond through a possible reaction between the phenolic hydroxyl group of the phenylethanoid unit and the ketone of the iridoid moiety. Verbenalinoside A (**1**) and B (**2**) are the first examples of the iridoid–phenylethanoid glycoside heterodimer, which not only enriches the chemical structural diversity of natural iridoids and phenylethanoids but also provides new insight into new hybrids of different types of natural compounds. Biological assays demonstrated that compounds **1**–**4** have hepatoprotective effects against ethanol-induced hepatotoxicity in LO2 and HepG2 cells, indicating that compounds **1**–**4** should be potential hepatoprotective constituents of *V. officinalis*.

## Experimental Procedures

### General Experimental Procedures

The extraction equipment used is the RTN-50 model thermal reflux extraction single-effect concentration unit from Zhejiang Jin’an Pharmaceutical Machinery Co., Ltd., People’s Republic of China. IR spectra were recorded with an IR Affinity-1S spectrometer from Shimadzu, Japan. UV spectra were acquired on a J-1500 circular dichroism spectrometer from JASCO, Japan. Optical rotations were measured by using a Rudolph Research Analytical Autopol I automatic polarimeter. NMR experiments were conducted on a Bruker Ascend-600 spectrometer (Bruker, Germany), utilizing deuterated methanol (CD_3_OD) as the solvent and tetramethylsilane (TMS) as an internal standard. HRESIMS data were collected on an Agilent-6230 LC/TOF mass spectrometer, which was operated in positive mode and interfaced with an Agilent 1260 HPLC system, both from Agilent Technologies. MPLC was carried out using a Buchi C-620 system (Buchi, Switzerland) fitted with a Siliabond C_18_ column (ODS gel, 5 μm, 36 mm × 460 mm). The flow rate was maintained at 20 mL/min, and UV detection was performed at 210 nm. Preparative HPLC separation was carried out using a WUFENG LC-100 HPLC system (Wufeng, People’s Republic of China) fitted with a YMC-Actus Triart C_18_ column (5 μm, 30 mm × 250 mm, YMC, Japan). Semipreparative HPLC separation was conducted using an Agilent 1200 HPLC system (Agilent) equipped with two columns: an XBridge BEH C_18_ column (5 μm, 10 mm × 250 mm, Waters) and an XTerra MS C_18_ column (5 μm, 10 mm × 250 mm, Waters). The separation was performed at a flow rate of 3 mL/min with UV detection at 210 and 254 nm. The column chromatography (CC) procedure utilized silica gel (100–200 mesh) (Qingdao Haiyang Chemical Co. Ltd., People’s Republic of China).

### Plant Material

The dried aerial parts of *V. officinalis* were collected from Nanyang of Henan Province, People’s Republic of China, in October 2021. The species was identified by Dr. Guo-yuan Zhu from the Macau University of Science and Technology. The samples were kept at the State Key Laboratory of Quality Research in Chinese Medicine, Macau University of Science and Technology.

### Extraction and Isolation

The dried aerial parts of *V. officinalis* (10.0 kg) were powdered and refluxed with 80% EtOH (40 L × 3) at 60 °C. After concentration under a vacuum range of −0.06 to −0.1 Pa, the residue (800 g) was diluted in water and extracted using PE, EtOAc, and *n*-BuOH, respectively. The EtOAc part (170 g) was subjected to silica gel CC, and eluted with a gradient of CH_2_Cl_2_–MeOH (from 100:1 to 5:1), yielding 11 fractions (Fr.E1–E11). Fr.E2 was separated by preparative HPLC (MeOH–H_2_O, 30:70) to obtain compound **3** (*t*_R_ = 21 min, 5.2 g). Fr.E5 was purified using MPLC on a Siliabond C_18_ column with a MeOH–H_2_O (30:70) eluent. Further purification was achieved by semipreparative HPLC with 17% MeCN as the eluent to give **4** (*t*_R_ = 17 min, 71.0 mg).

The *n*-BuOH fraction (274 g) was separated using MPLC eluted with a gradient mobile phase of MeOH–H_2_O (30:70 to 100:0) to yield 25 fractions (Fr.B1–B25). Fr.B19 was further chromatographed into eight fractions (Fr.B19–1–8) by MPLC eluted with MeCN–H_2_O (10:90 to 100:0). Fr.B19–8 was purified by MPLC (30% MeCN in H_2_O) to obtain Fr.B19–8–1–7. Fr.B19–8–2 was subjected to further purification via semipreparative HPLC using a mobile phase of MeOH-0.1% FA (41:59) to afford **2** (t_*R*_ = 23 min, 17.0 mg). Compound **1** (*t*_R_ = 17 min, 7.4 mg) was purified from Fr.B19–8–4 using semipreparative HPLC with a mobile phase of MeOH-0.1% FA (40:60).

### Verbenalinoside A (**1**)

Yellow powder; [α]_D_^25^ −9.5 (*c* 0.5, MeOH); UV (MeOH) λ_max_ (log ε) 203 (5.19), 280 (4.55), 330 (4.73) nm; IR (KBr) ν_max_ 3333, 1713, 1697, 1604, 1519, 1273, 1042, 810 cm^–1^; ^1^H (CD_3_OD, 600 MHz) and ^13^C NMR (CD_3_OD, 150 MHz) data, see Table 1; HRESIMS *m*/*z* 1028.3611 [M + NH_4_] ^+^ (calcd for C_46_H_62_NO_25_, 1028.3605).

### Verbenalinoside B (**2**)

Yellow powder; [α]_D_^25^ −18.8 (*c* 0.5, MeOH); UV (MeOH) λ_max_ (log ε) 203 (5.07), 280 (4.32), 332 (6.20) nm; IR (KBr) ν_max_ 3402, 1705, 1605, 1512, 1443, 1373, 1281, 1065, 1042 cm^–1^; ^1^H (CD_3_OD, 600 MHz) and ^13^C NMR (CD_3_OD, 150 MHz) data, see Table 1; HRESIMS *m*/*z* 1028.3605 [M + NH_4_]^+^ (calcd for C_46_H_62_NO_25_, 1028.3605).

### ECD Calculations

In ECD calculations, conformational analyses revealed one lowest energy conformer of **1** and five lowest energy conformers of **2** (the key part structure of unit A and the phenylethanoid moiety in unit B, Figure 4). The detailed TD-DFT calculation method was shown in our previous study.^21,22^ All conformers and ECD calculation raw data are presented in the Supporting Information (Figures S25 and S28 and Tables S5–S7).

### Conformational Search and DP4 Plus Analysis

According to our previous DP4+ NMR calculation method,^21^ nine lowest energy conformers for isomers of **1** and **2** (the key part structure of unit A and the phenylethanoid moiety in unit B, Figure 4) were determined. The NMR calculation raw data are shown in the Supporting Information (Figures S23, S24, S26, and S27 and Tables S3 and S4).

### Cell Lines and Cell Culture

The LO2 and HepG2 cell lines were sourced from ATCC in the USA. LO2 cells were cultured in the Roswell Park Memorial Institute 1640 (RPMI-1640) medium, while HepG2 cells were maintained in Dulbecco’s modified Eagle’s medium (DMEM). The culture media for both cell lines were supplemented with 10% fetal bovine serum (FBS) and a 1% antibiotic mixture. The cells were maintained in a humidified incubator at 37 °C with 5% CO_2_.

### Cell Viability

LO2 and HepG2 cells were plated at a density of 1 × 10^4^ cells per well in 96-well plates and allowed to incubate for an overnight period. Two cell lines were treated with or without 40 μM compounds **1**–**4** for 24 h. Following this treatment, each well was treated with 20 μL of MTT reagent (5 mg/mL) and incubated for an additional 4 h. Subsequently, the optical density (OD) was measured at 570 nm using a microplate reader. Resveratrol (ResV, 10 μM) was used as the positive for all the experiments.^23^

### Hepatoprotective Activity

The LO2 and HepG2 cell lines were seeded into 96-well plates at a density of 1 × 10^4^ cells per well and incubated for 24 h. Subsequently, compounds **1**–**4** (1.25, 2.5, 5, 10, 20, and 40 μM) and the positive control (ResV,10 μM) were added to the wells, and the plates were incubated for 2 h. Cells were then treated with or without 3% ethanol for another 24 h. The cell viability was determined using the MTT assay. All samples were tested in three independent experiments.

## Supporting Information Available

^1^H and ^13^C NMR data of **3** and **4**; 1D and 2D NMR, HRESIMS, and IR spectra of **1** and **2**; NMR calculation data of **1** and **2** (PDF)

## References

[ref1] Kubica P.; Szopa A.; Dominiak J.; Luczkiewicz M.; Ekiert H. *Verbena officinalis* (Common Vervain) – A Review on the investigations of this medicinally important plant species. Planta Med. 2020, 86, 1241–1257. 10.1055/a-1232-5758. 32937665

[ref2] Zhang X.-L. The research overview on the effective components and bioactivity of *Verbena officinalis*. China Pract. Med. 2009, 4, 232–235.

[ref3] Kubica P.; Szopa A.; Prokopiuk B.; Komsta L.; Pawlowska B.; Ekiert H. The influence of light quality on the production of bioactive metabolites – verbascoside, isoverbascoside and phenolic acids and the content of photosynthetic pigments in biomass of *Verbena officinalis* L. cultured *in vitro*. J. Photochem. Photobiol., B 2020, 203, 111768 10.1016/j.jphotobiol.2019.111768. 31931383

[ref4] He J.; Fan Y.-Q.; Yang F.-W.; Huang M.; Zhang J.-H.; Zhang B.-L. Research advances on chemical components and pharmacological activities of *Verbena officinals*. Tianjin J. Tradit. Chin. Med. 2020, 37, 1205–1212.

[ref5] Shu J.-C.; Chou G.-X.; Wang Z.-T. Two new iridoids from *Verbena officinalis* L. Molecules 2014, 19, 10473 10.3390/molecules190710473. 25045888 PMC6271327

[ref6] Li J.; Dong D.-Y.; Peng C.-Y.; Yang Q.; Liu J.-Q.; Shu J.-C. Phenylethanoid glycosides from *Verbenae herba*. Chin. Tradit. Pat. Med. 2024, 46, 137–142.

[ref7] Rehecho S.; Hidalgo O.; de Cirano M. G.-Ĩ.; Navarro I.; Astiasaran I.; Ansorena D.; Cavero R.-Y.; Calvo M. I. Chemical composition, mineral content and antioxidant activity of *Verbena officinalis* L. LWT - Food Sci. Technol. 2011, 44, 875–882. 10.1016/j.lwt.2010.11.035.

[ref8] Kubica P.; Szopa A.; Ekiert H. Production of verbascoside and phenolic acids in biomass of *Verbena officinalis* L. (vervain) cultured under different *in vitro* conditions. Nat. Prod. Res. 2017, 31, 1663–1668. 10.1080/14786419.2017.1286477. 28278649

[ref9] Yang J.-Y.; Guo C.-S.; Su L.; Xu C. X.; Li R.-T.; Zhong J.-D. Four undescribed triterpenes from the aerial parts of *Verbena officinalis*. Fitoterapia 2023, 170, 105670 10.1016/j.fitote.2023.105670. 37690598

[ref10] Casanova E.; Garcia-Mina J.-M.; Calvo M.-I. Antioxidant and antifungal activity of *Verbena officinalis* L. leaves. Plant Foods Hum. Nutr. 2008, 63, 93–97. 10.1007/s11130-008-0073-0. 18498054

[ref11] Kou W.-Z.; Yang J.; Yang Q.-H.; Wang Y.; Wang Z.-F.; Liu J. Study on *In-Vivo* Anti-Tumor Activity of *Verbena officinalis* Extract. Afr. J. Tradit., Complementary Altern. Med. 2013, 10, 512–517. 10.4314/ajtcam.v10i3.19 PMC3777594 24146482

[ref12] Ahmed D.; Chaudhary M. A.; Raza A.; Waheed A.; Khan S.-R.; Ikram M. Comparative Study of Antibacterial Activity and Mineral Contents of Various Parts of *Verbena officinalis* Linn. Asian J. Chem. 2012, 24, 68–72.

[ref13] Calvo M.-I. Anti-inflammatory and analgesic activity of the topical preparation of *Verbena officinalis* L. J. Ethnopharmacol. 2006, 107, 380–382. 10.1016/j.jep.2006.03.037. 16723201

[ref14] Wang M.-Y.; Jia M.-R. An overview of the medicinal use of *Verbena officinalis* by 25 ethnic groups in China. J. Med. Pharm. Chin. Min. 2002, 8, 20–21.

[ref15] Zhang H.-J.; Rothwangl K.; Mesecar A.-D.; Sabahi A.; Rong L.-J.; Fong H. H.-S. Lamiridosins, Hepatitis C Virus Entry Inhibitors from *Lamium album*. J. Nat. Prod. 2009, 72, 2158–2162. 10.1021/np900549e. 19904996

[ref16] Dong J.-H.; Du C.-L.; Xu C.-T.; Wang Q.; Wang Z.-H.; Zhu Q.; Lv X.-W.; Zhang L.; Li J.; Huang C.; Wang H.; Ma T.-T. Verbenalin attenuates hepatic damage and mitochondrial dysfunction in alcohol–associated steatohepatitis by regulating MDMX/PPARα-mediated ferroptosis. J. Ethnopharmacol. 2023, 307, 116227 10.1016/j.jep.2023.116227. 36739928

[ref17] Du C.-L.; Dong J.-H.; Wang Q.; Xu C.-T.; Feng S.-Q.; Feng R.; Lv X.-W.; Li J.; Zhang L.; Huang C.; Huang C.; Ma T.-T. Hastatoside attenuates carbon tetrachloride-induced liver fibrosis by targeting glycogen synthase kinase-3β. Phytomedicine 2023, 109, 154585 10.1016/j.phymed.2022.154585. 36610117

[ref18] Teborg D.; Junior P. Iridoid Glucosides from *Penstemon nitidus*. Planta Med. 1991, 57, 184–186. 10.1055/s-2006-960062. 17226148

[ref19] Wu J.; Huang J.-S.; Xiao Q.; Zhang S.; Xiao Z.-H.; Li Q.-X.; Long L.-J.; Huang L.-M. Complete assignments of ^1^H and ^13^C NMR data for 10 phenylethanoid glycosides. Magn. Reson. Chem. 2004, 42, 659–662. 10.1002/mrc.1393. 15181637

[ref20] Vestena A.; Piton Y.; Bordignon S.-A.-L.; Garcia S.; Arbo M.-D.; Zuanazzi J.-A.; Poser G.-V. Hepatoprotective activity of *Verbena litoralis*, Verbena montevidensis and their main iridoid, brasoside. J. Ethnopharmacol. 2019, 239, 111906 10.1016/j.jep.2019.111906. 31028856

[ref21] Chen F.-L.; Liu D.-L.; Fu J.; Fu L.; Gao J.; Bai L.-P.; Zhang W.; Jiang Z.-H.; Zhu G.-Y. Atrachinenynes A–D, four diacetylenic derivatives with unprecedented skeletons from the rhizomes of *Atractylodes chinensis*. New J. Chem. 2022, 46, 15530–15537. 10.1039/D2NJ02149H.

[ref22] Ren W.-J.; Io C.-C.; Jiang R.; Ng K.-F.; Liu J.-Z.; Bai L.-P.; Zhang W.; Jiang Z.-H.; Liu Y.-H.; Zhu G.-Y. Di- and Triterpenoids from the Rhizomes of Isodon amethystoides and Their Anti-inflammatory Activities. J. Nat. Prod. 2023, 86, 1230–1239. 10.1021/acs.jnatprod.2c01136. 37146221

[ref23] Chupradit S.; Bokov D.; Zamanian M.-Y.; Heidari M.; Hakimizadeh E. Hepatoprotective and therapeutic effects of resveratrol: A focus on anti-inflammatory and antioxidative activities. Fundam. Clin. Pharmacol. 2022, 36, 468–485. 10.1111/fcp.12746. 34935193

