# Supplementary material for: Verbenalinosides A and B, Two Iridoid–Phenylethanoid Glycoside Conjugates from Verbena officinalis and Their Hepatoprotective Activity
Source: ACS Omega. 2025 Feb 27;10(9):9684–90. doi: 10.1021/acsomega.4c11149 (PMC11904641; doi:10.1021/acsomega.4c11149)
Supplement: Supplementary file 1 — ao4c11149_si_001.pdf [file ao4c11149_si_001.pdf]

# Supporting information

# Verbenalinosides A and B, Two iridoid-phenylethanoid glycoside conjugates from the *Verbena officinalis* and Their Hepatoprotective Activity

Xiao-Mei Liu,[1] Wen-Jing Ren,[2] Hao-Yuan Lyu,[2] Cheng-Yu Chen,[4] Xiao-Hui Pan,[4] Li-Ping Bai,[2] Wei Zhang,[2] Zhi-Hong Jiang,*[,2] and Guo-Yuan Zhu *[,2,3]

[1]*State Key Laboratory of Quality Research in Chinese Medicine & Faculty of Chinese Medicine, Macau University of Science and Technology, Macau 999078, People's Republic of China*

[2] *State Key Laboratory of Quality Research in Chinese Medicine, Macau Institute for Applied Research in Medicine and Health, Macau University of Science and Technology, Macau 999078, People's Republic of China*

[3]*Zhuhai MUST Science and Technology Research Institute, Macau University of Science and Technology, Zhuhai 519000, People's Republic of China*

[4]*National Engineering Research Center for Modernization of Traditional Chinese Medicine, Grand TCM Product-Cultivating Branch, Jiaheng (Zhuhai Hengqin) Pharmaceutical Technology Co., Ltd., Zhuhai 519000, People's Republic of China*

# Table of Contents

Table S1. $^{1}$H NMR (600 MHz) and $^{13}$C NMR (150 MHz) Data of Verbenalin (**3**) ($\delta$ in ppm, $CD_3OD$) ........ S3
Table S2. $^{1}$H NMR (600 MHz) and $^{13}$C NMR (150 MHz) Data of Verbascoside (**4**) ($\delta$ in ppm, $CD_3OD$) ........ S4
Figure S1. HRESIMS spectrum of verbenalinoside A (**1**)........ S5
Figure S2. $^{1}$H NMR spectrum of verbenalinoside A (**1**)........ S5
Figure S3. $^{13}$C NMR spectrum of verbenalinoside A (**1**)........ S6
Figure S4. DEPT-135 NMR spectrum of verbenalinoside A (**1**)........ S6
Figure S5. $^{1}$H-$^{1}$H COSY spectrum of verbenalinoside A (**1**)........ S7
Figure S6. HSQC spectrum of verbenalinoside A (**1**) ........ S7
Figure S7. HMBC spectrum of verbenalinoside A (**1**) ........ S8
Figure S8. NOESY spectrum of verbenalinoside A (**1**)........ S8
Figure S9. IR Value of verbenalinoside A (**1**) ........ S9
Figure S10. UV spectrum of verbenalinoside A (**1**) ........ S9
Figure S11. CD spectrum of verbenalinoside A (**1**) ........ S10
Figure S12. HRESIMS spectrum of verbenalinoside B (**2**)........ S10
Figure S13. $^{1}$H NMR spectrum of verbenalinoside B (**2**)........ S11
Figure S14. $^{13}$C NMR spectrum of verbenalinoside B (**2**)........ S11
Figure S15. DEPT-135 NMR spectrum of verbenalinoside B (**2**)........ S12
Figure S16. $^{1}$H-$^{1}$H COSY spectrum of verbenalinoside B (**2**)........ S12
Figure S17. HSQC spectrum of verbenalinoside B (**2**)........ S13
Figure S18. HMBC spectrum of verbenalinoside B (**2**) ........ S13
Figure S19. NOESYspectrum of verbenalinoside B (**2**)........ S14
Figure S20. IR Value of verbenalinoside B (**2**) ........ S14
Figure S21. UV spectrum of verbenalinoside B (**2**) ........ S15
Figure S22. CD spectrum of verbenalinoside B (**2**)........ S15
Figure S23. Nine lowest energy conformers of **1** ( Unit A and the phenylethanoid moiety in unit B) by DP4+ calculation. ........ S16
Figure S24. DP4 plus Calculation Results of compound **1** ( Unit A and the phenylethanoid moiety in unit B) ........ S16
Figure S25. DFT computational optimized conformations of (1a*R*,3a*R*,4a*S*,5a*S*,10a*S*,1'a*S*,2'a*R*,3'a*S*,4'a*S*,5'a*R*)**-1** (Unit A and the phenylethanoid moiety in unit B) ........ S17

Figure S26. Nine lowest energy conformers of **2** (Unit A and the phenylethanoid moiety in unit B) by DP4+ calculation. ......................................................................S17
Figure S27. DP4 plus Calculation Results of compound **2** (Unit A and phenylethanoid moiety in unit B) ..........................................................................................S18
Figure S28. DFT computational optimized conformations of (1a*S*,5a*S*,6a*R*,8a*S*,9a*R*,1'a*S*,2'a*R*,3'a*S*,4'a*S*,5'a*R*)**-2** (Unit A and the phenylethanoid moiety in unit B) ..........................................................................................S18
Table S3. Cartesian coordinates for the low-energy reoptimized random research conformers of compound **1** (Unit A and the phenylethanoid moiety in unit B) (obtained by DP4+ calculation) .....................................................................................S19
Table S4. Cartesian coordinates for the low-energy reoptimized random research conformers of compound **2** (Unit A and the phenylethanoid moiety in unit B) (obtained by DP4+ calculation) .....................................................................................S35
Table S5. Experimental and calculated $^{13}$C chemical shift values of the part structure of **1** and **2** including unit A and the phenylethanoid moiety in unit B ..........................S51
Table S6. Cartesian coordinates for the low-energy reoptimized random research conformers of compound **1** (unit A and the phenylethanoid moiety in unit B) (obtained by ECD calculation)...................................................................................S52
Table S7. Cartesian coordinates for the low-energy reoptimized random research conformers of compound **2** (unit A and the phenylethanoid moiety in unit B) (obtained by ECD calculation)...................................................................................S54

**Table S1.** $^{1}H$ NMR (600 MHz) and $^{13}C$ NMR (150 MHz) Data of Verbenalin **(3)** ($\delta$ in ppm, $CD_3OD$)

| No. | $\delta_C$ | $\delta_H$ ($J$ in Hz) | $\delta_C$*[1] | $\delta_H$ ($J$ in Hz)*[1] |
|---|---|---|---|---|
| 1a | 97.1 | 5.24 (1H, d, 7.0) | 97.07 | 5.25 (1H, d, 7.1) |
| 3a | 153.8 | 7.46 (1H, d, 1.2) | 153.77 | 7.48 (1H, d, 1.3) |
| 4a | 105.5 | | 105.43 | |
| 5a | 43.6 | 3.52 (1H, d, 8.1) | 43.52 | 3.53 (1H, br.d, 8.1) |
| 6a | 215.8 | | 215.78 | |
| 7a | 43.7 | 2.01 (1H, dd, 4.0, 18.9 Hz) | 43.69 | 2.02 (1H, dd, 3.8, 17.8 Hz) |
| | | 2.55 (1H, dd, 8.8, 18.9 Hz) | | 2.57 (1H, dd, 8.7, 17Hz) |
| 8a | 29.9 | 2.48 (1H， m) | 29.85 | 2.51 (1H, m) |
| 9a | 45.5 | 2.21 (1H, m) | 45.45 | 2.24 (1H, dt, 3.7,7.4,7.7) |
| 10a | 20.6 | 1.23 (3H， d, 6.9) | 20.57 | 1.24（3H, d, 6.8) |
| 11a | 168.9 | | 168.77 | |
| 12a | 52.0 | 3.72 (s) | 51.97 | 3.74 (s) |
| 1'a | 100.5 | 4.66 (1H, d, 7.9 Hz) | 100.54 | 4.65 (1H， d, 7.8 Hz) |
| 2'a | 74.7 | 3.22 (1H, m) | 74.60 | —— |
| 3'a | 78.0 | 3.36 (1H, d, 8.9) | 77.90 | —— |
| 4'a | 71.6 | 3.26 (1H, m) | 71.53 | —— |
| 5'a | 78.4 | 3.29 (1H, m) | 78.29 | —— |
| 6'a | 62.8 | 3.87 (1H, dd, 2.4, 12.0) | 62.71 | 3.91 (1H, dd, 1.9, 12.0) |
| | | 3.64 (1H, dd, 5.6, 12.0) | | 3.66 (1H, dd, 5.7, 12.0) |

* The literature NMR data of verbenalin.

[1] Teborg, D.; Junior, P. Iridoid Glucosides from *Penstemon nitidus*. *Planta Med.* **1991**, *57*, 184–186.

**Table S2.** $^{1}H$ NMR (600 MHz) and $^{13}C$ NMR (150 MHz) Data of Verbascoside **(4)** ($\delta$ in ppm, $CD_3OD$)

| No. | $\delta_C$ | $\delta_H$ *(J in Hz)* | $\delta_C$*[2] | $\delta_H$ (*J* in Hz)*[2] |
|---|---|---|---|---|
| Aglycone | | | | |
| 1b | 130.1 | | 131.52 | |
| 2b | 115.7 | 6.70 (1H, d, 2.0) | 116.35 | 6.71 (1H, d, 2.0) |
| 3b | 145.4 | | 146.13 | |
| 4b | 143.3 | | 144.67 | |
| 5b | 114.9 | 6.68 (1H, d, 8.2) | 117.15 | 6.68 (1H, d, 8.0) |
| 6b | 119.8 | 6.58 (1H, dd, 2.0, 8.2) | 121.29 | 6.57 (1H, dd, 2.0, 8.0) |
| 7b | 35.1 | 2.80 (2H, m) | 36.56 | 2.76 (2H, m) |
| 8b | 70.8 | 4.05 (1H, m) | 72.09 | 4.05 (1H, dd,17.0, 8.0) |
| | | 3.72 (1H, m) | | 3.72 (1H, dd, 17.0, 8.0) |
| Glucose | | | | |
| 1'b | 102.8 | 4.38 (1H, d, 7.9) | 104.21 | 4.38 (1H, d, 8.0) |
| 2'b | 74.6 | 3.39 (1H, dd, 8.0 , 9.1) | 76.22 | 3.40 (1H, dd, 8.0 , 9.0) |
| 3'b | 80.2 | 3.82 (1H, t, 9.2) | 81.66 | 3.81 (1H, t, 9.2) |
| 4'b | 69.0 | 4.91 (1H, d, 9.5) | 70.62 | 4.91 (1H, d, 9.6) |
| 5'b | 74.8 | 3.52 (1H, t, 6.0) | 76.03 | 3.53 |
| 6'b | 60.9 | 3.62 (1H, d, 10.0) | 62.39 | 3.62 |
| | | 3.58 (1H, t, 2.6) | | 3.52 |
| Rhamnose | | | | |
| 1"b | 101.6 | 5.19 (1H, d, 1.6) | 103.02 | 5.20 (1H, d, 1.4) |
| 2"b | 70.9 | 3.92 (1H, m) | 72.37 | 3.93 (1H, br.s.) |
| 3"b | 70.6 | 3.57 (1H, dd, 1.8, 7.2) | 72.25 | 3.58 |
| 4"b | 72.3 | 3.27 (1H, t, 10.8) | 73.99 | 3.30 (1H, t, 9.5) |
| 5"b | 69.2 | 3.54 (1H, d, 5.6) | 70.04 | 3.55 |
| 6"b | 17.0 | 1.09 (3H, d, 6.2) | 18.47 | 1.08 (3H, d, 6.0) |
| Caffeic acid | | | | |
| 1"'b | 126.2 | | 127.69 | |
| 2"'b | 113.8 | 7.06 (1H, d, 2.0) | 115.29 | 7.06 (1H, d, 1.8) |
| 3"'b | 144.7 | | 146.83 | |
| 4"'b | 148.4 | | 149.78 | |
| 5"'b | 115.1 | 6.78 (1H, d, 8.2) | 116.55 | 6.78 (1H, d, 8.2) |
| 6"'b | 121.8 | 6.96 (1H, dd, 2.0, 8.2) | 123.23 | 6.96 (1H, dd, 1.8, 8.2) |
| 7"'b | 146.6 | 7.59 (1H, d, 15.9) | 148.02 | 7.59 (1H, d, 16) |
| 8"'b | 113.3 | 6.28 (1H, d, 15.9) | 114.75 | 6.25 (1H, d, 16) |
| 9"'b | 166.9 | | 168.31 | |

* The literature NMR data of verbascoside.

[2] Wu, J.; Huang, J.-S.; Xiao, Q.; Zhang, S.; Xiao, Z.-H.; Li, Q.-X.; Long, L.-J.; Huang, L.-M. Complete assignments of $^{1}$H and $^{13}$C NMR data for 10 phenylethanoid glycosides. *Magn. Reson. Chemi.* **2004**, *42*, 659–662.

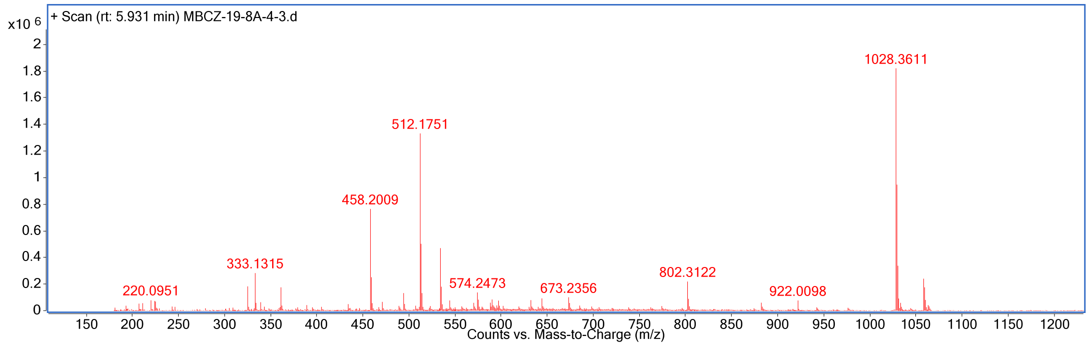


| Formula (M) | Score(MFG) | Mass | Mass (MFG) | m/z (Calc) | Diff (ppm) | Ion Formula | *m/z* |
|---|---|---|---|---|---|---|---|
| $C_{46}H_{58}O_{25}$ | 99.75 | 1010.3273 | 1010.3267 | 1028.3605 | -0.55 | $C_{46}H_{62}NO_{25}$ | 1028.3611 |

**Figure S1.** HRESIMS spectrum of verbenalinoside A (**1**)

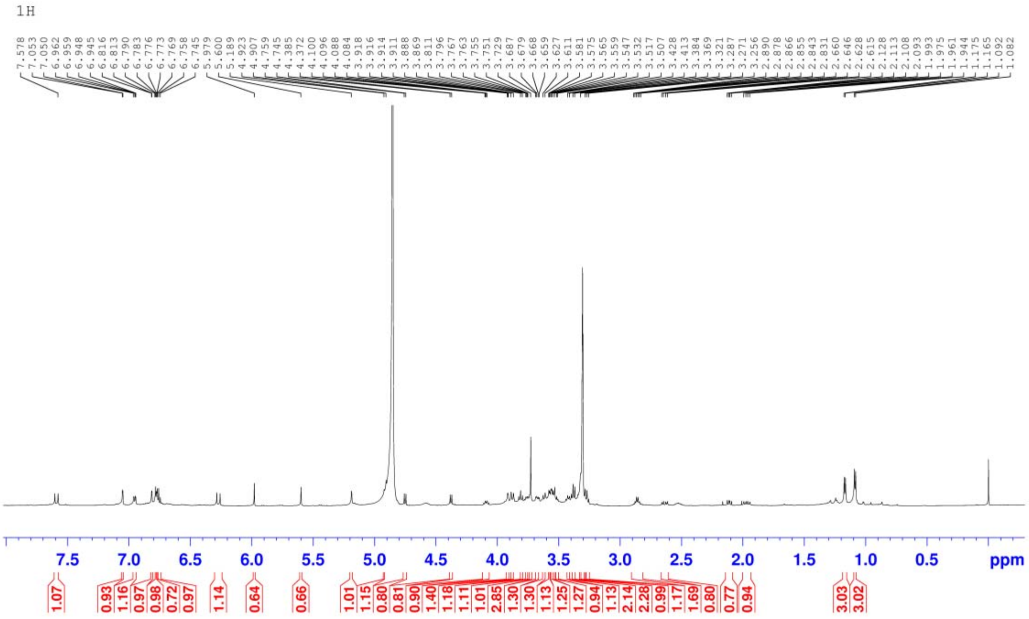


**Figure S2.** $^{1}$H NMR spectrum of verbenalinoside A (**1**)

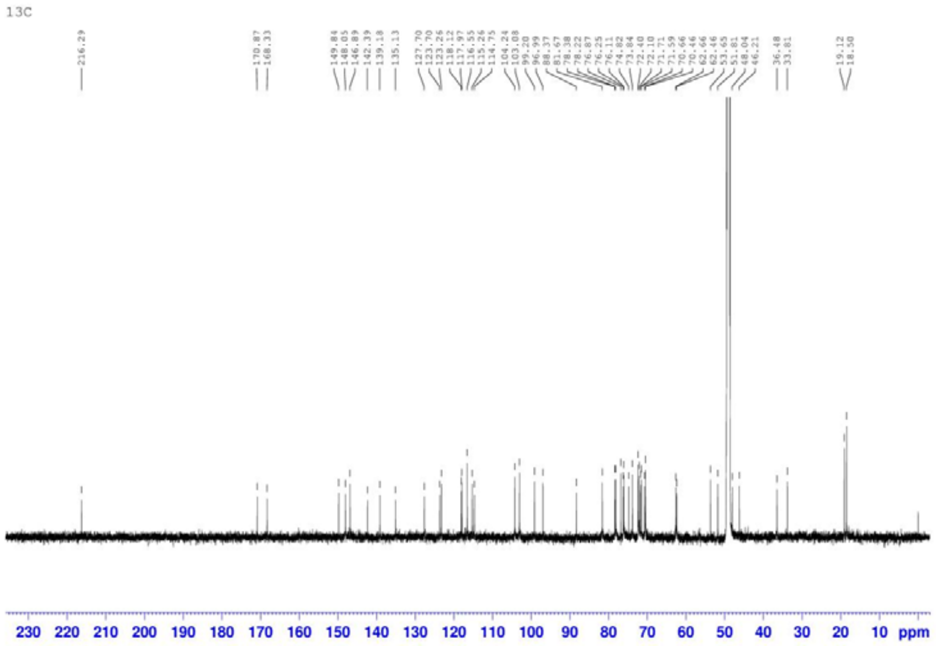

**Figure S3.** $^{13}C$ NMR spectrum of verbenalinoside A (**1**)

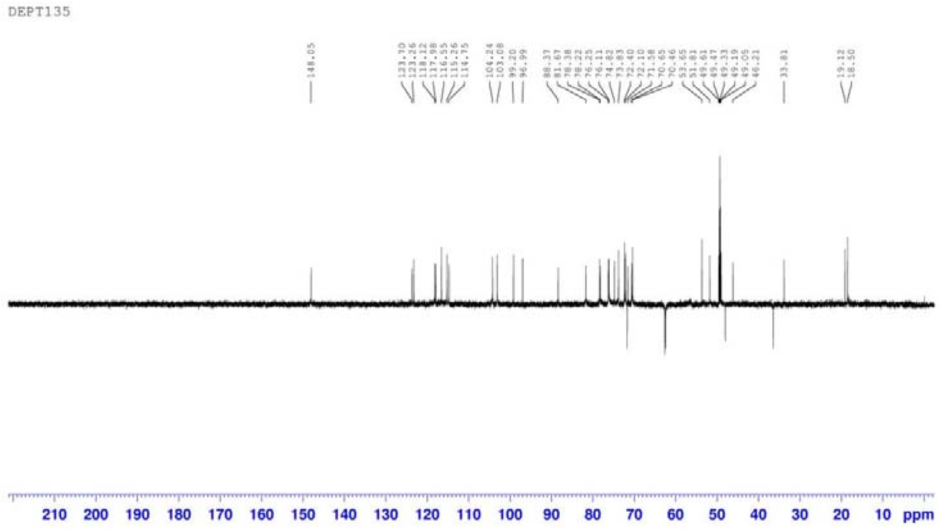

**Figure S4.** DEPT-135 NMR spectrum of verbenalinoside A (**1**)

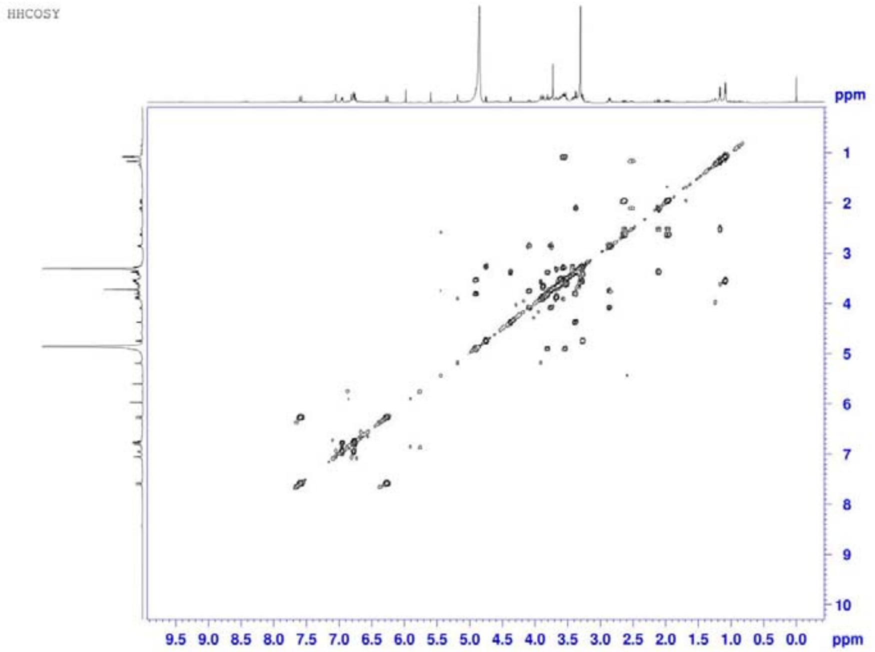


**Figure S5.** $^{1}H$-$^{1}H$ COSY spectrum of verbenalinoside A (**1**)

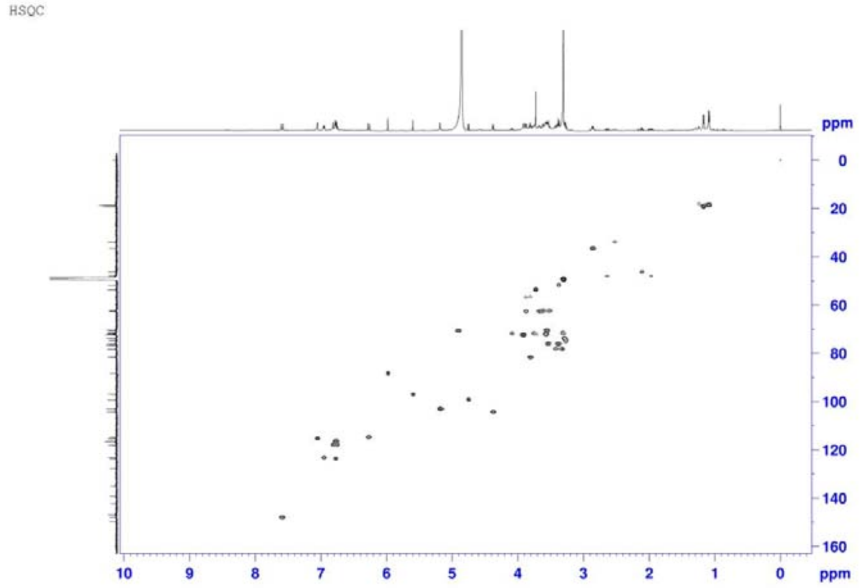


**Figure S6.** HSQC spectrum of verbenalinoside A (**1**)

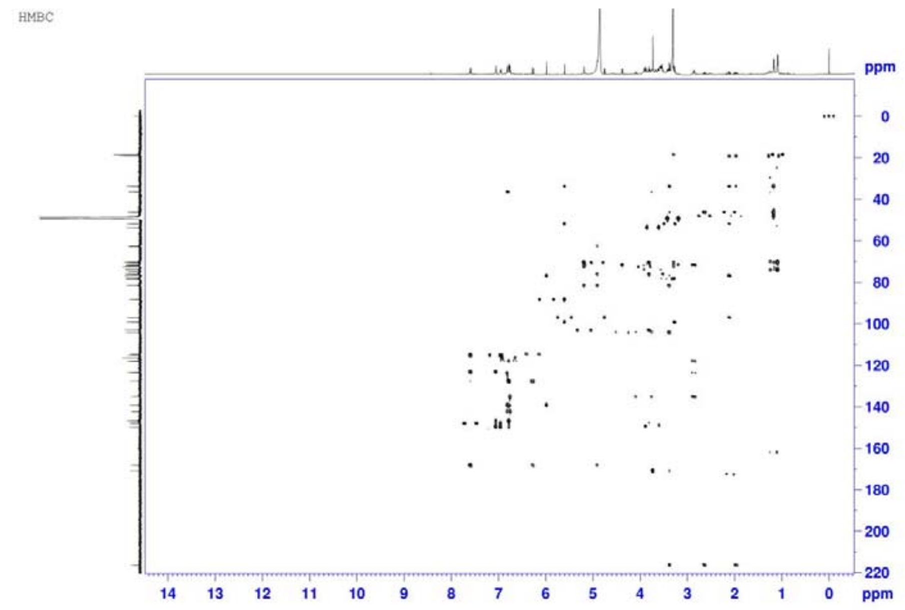


**Figure S7.** HMBC spectrum of verbenalinoside A (**1**)

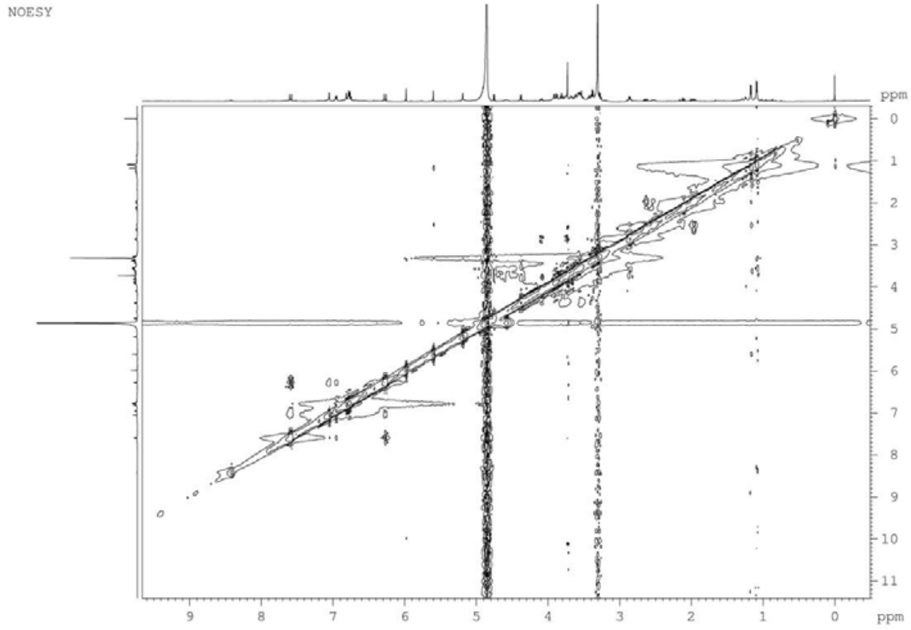


**Figure S8.** NOESY spectrum of verbenalinoside A (**1**)

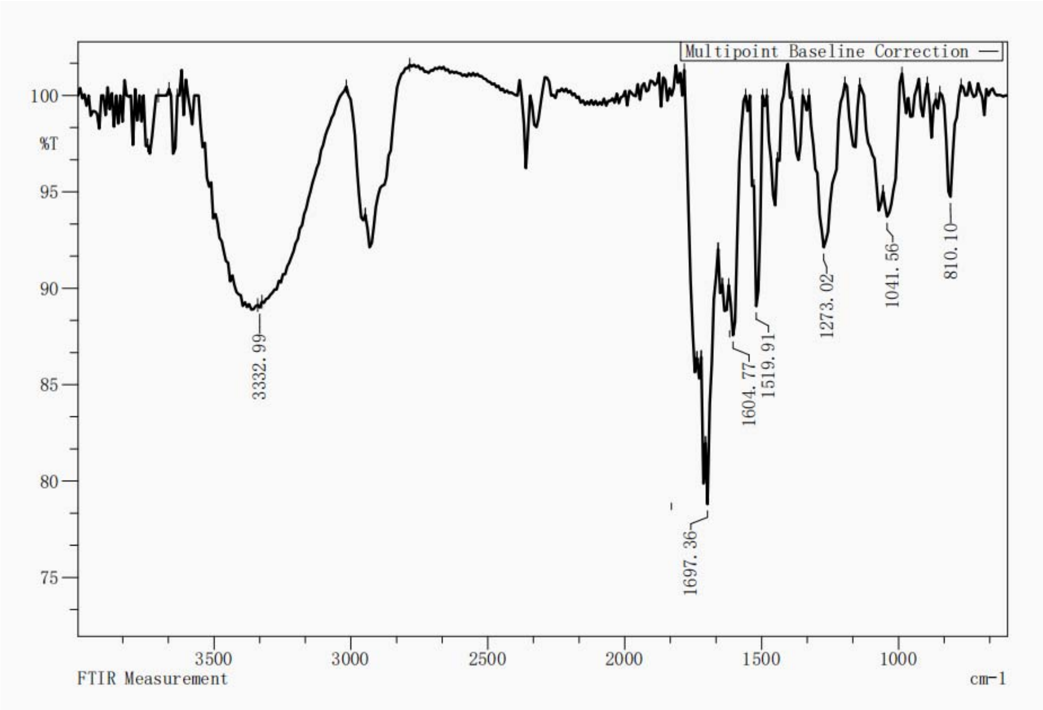


**Figure S9.** IR Value of verbenalinoside A (**1**)

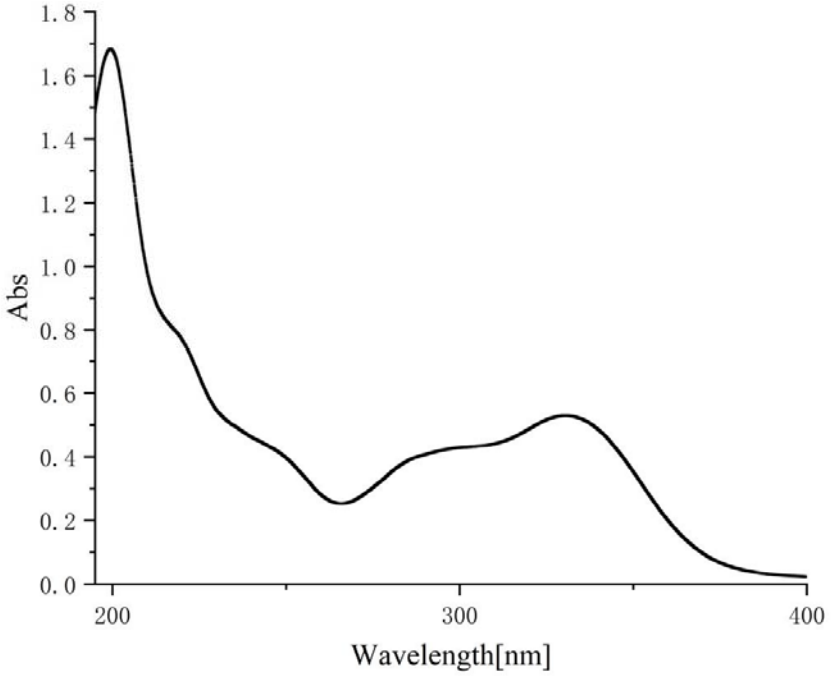


**Figure S10.** UV spectrum of verbenalinoside A (**1**)

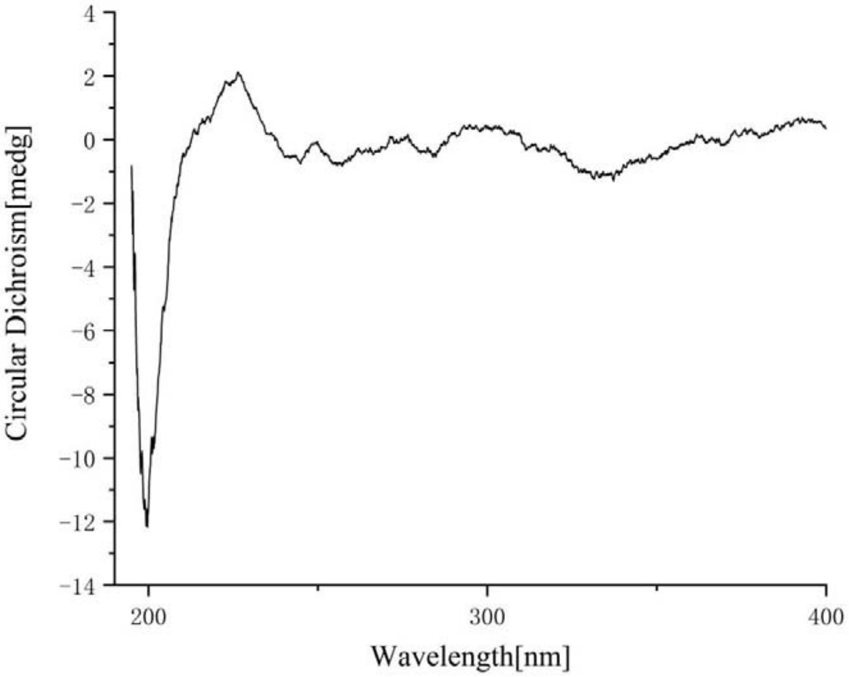


**Figure S11.** CD spectrum of verbenalinoside A (**1**)

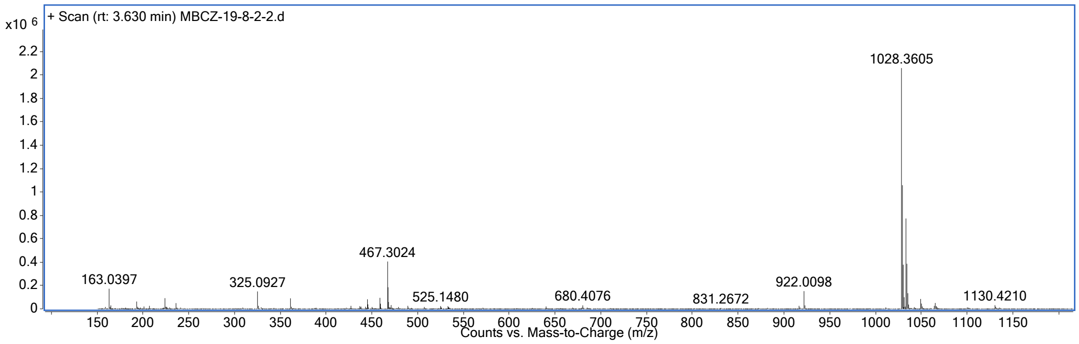


| Formula (M) | Score(MFG) | Mass | Mass (MFG) | m/z (Calc) | Diff (ppm) | Ion Formula | *m/z* |
|---|---|---|---|---|---|---|---|
| $C_{46}H_{58}O_{25}$ | 100 | 1010.3267 | 1010.3267 | 1028.3605 | 0.04 | $C_{46}H_{62}NO_{25}$ | 1028.3605 |

**Figure S12.** HRESIMS spectrum of verbenalinoside B (**2**)

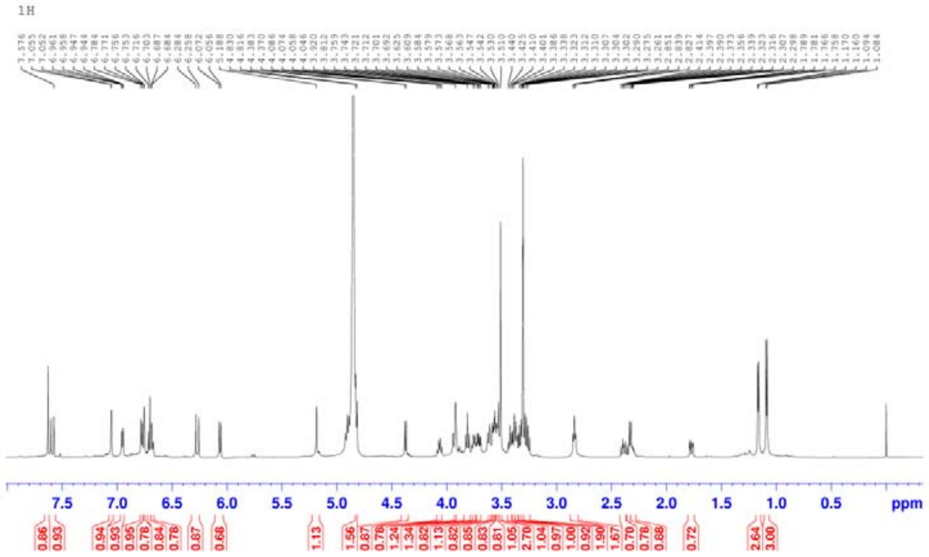

**Figure S13.** $^{1}$H NMR spectrum of verbenalinoside B (**2**)

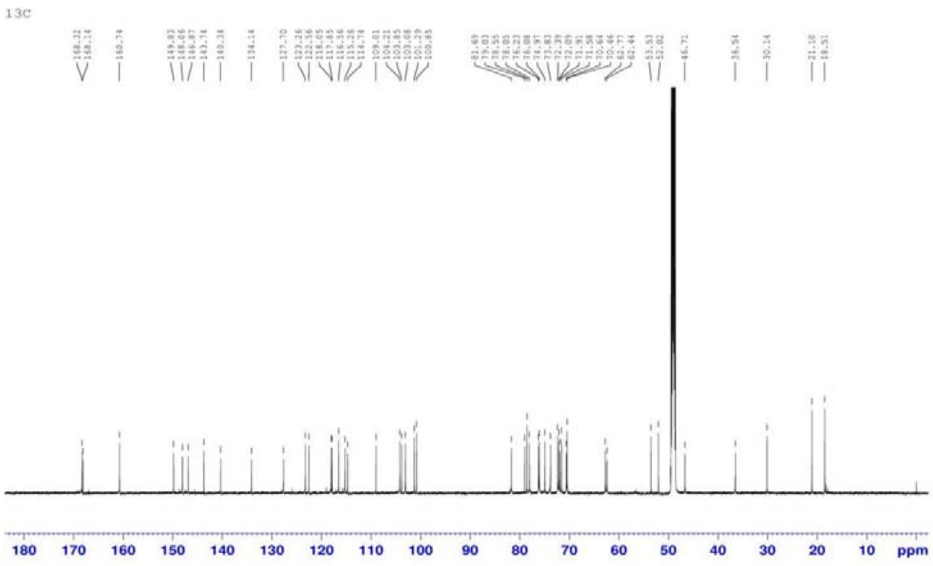

**Figure S14.** $^{13}$C NMR spectrum of verbenalinoside B (**2**)

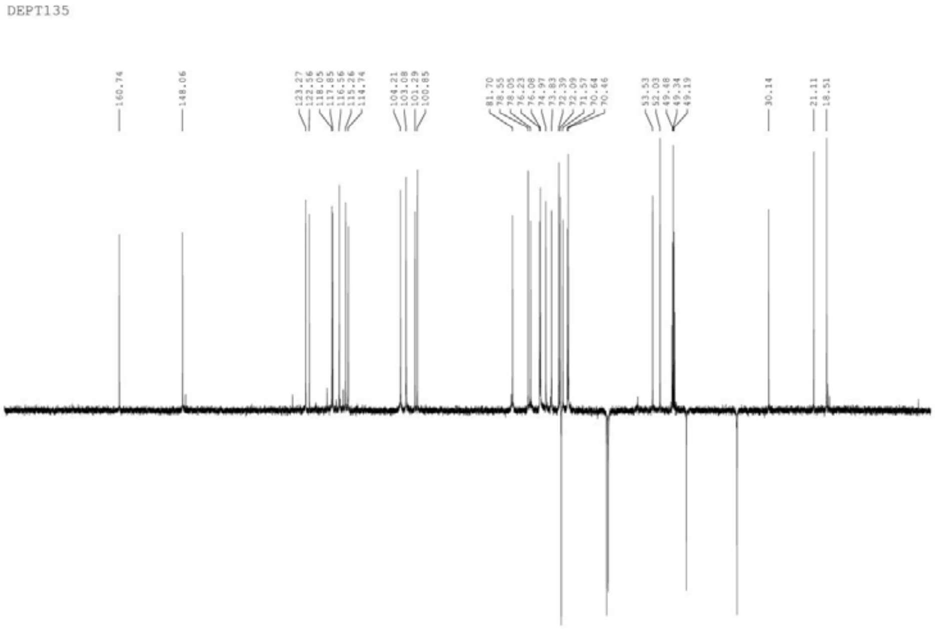


**Figure S15.** DEPT-135 NMR spectrum of verbenalinoside B (**2**)

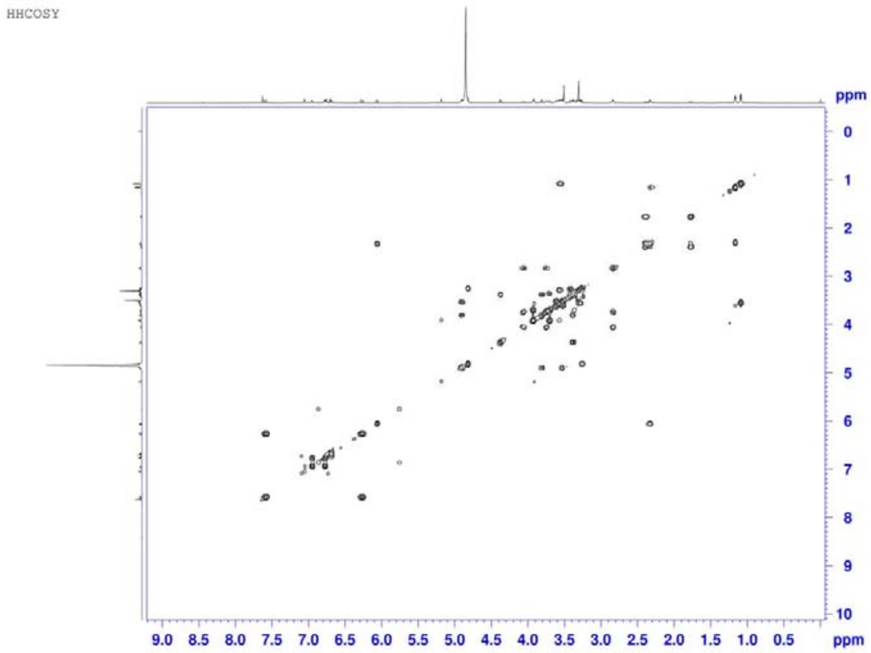


**Figure S16.** $^{1}H$-$^{1}H$ COSY spectrum of verbenalinoside B (**2**)

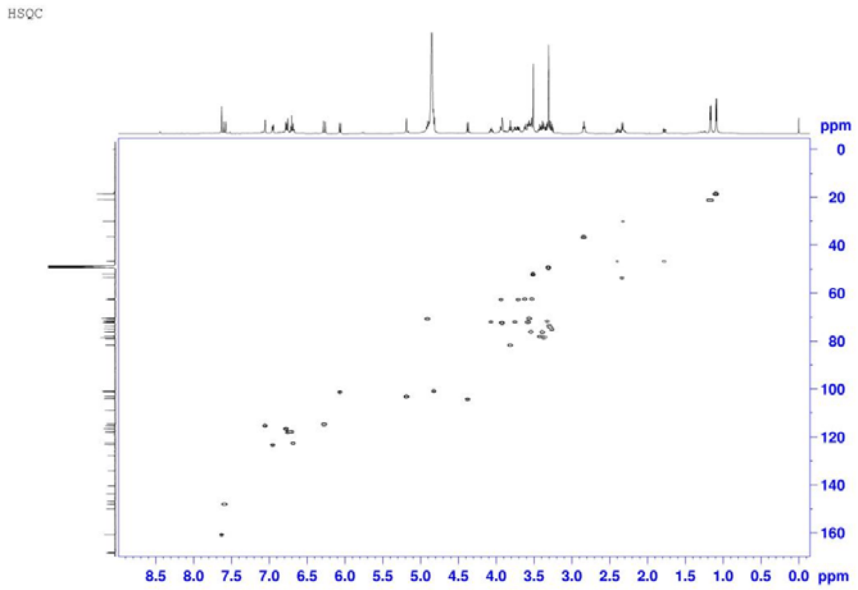


**Figure S17.** HSQC spectrum of verbenalinoside B (**2**)

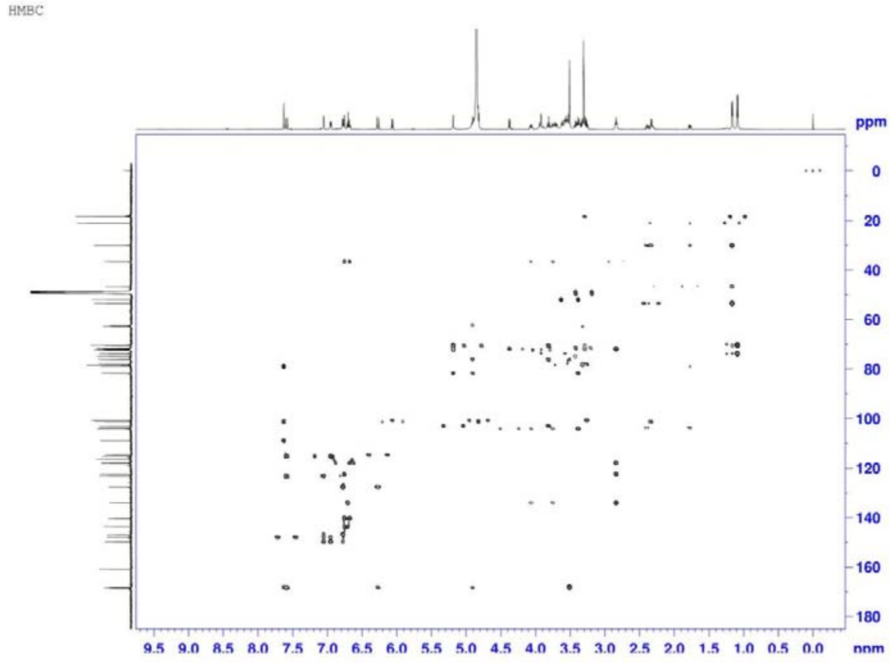


**Figure S18.** HMBC spectrum of verbenalinoside B (**2**)

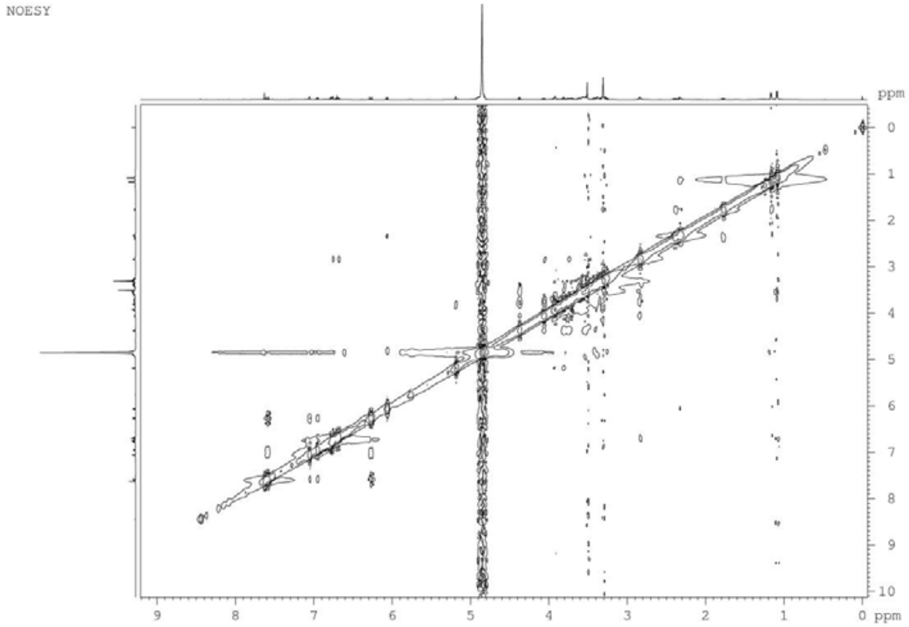


**Figure S19.** NOESYspectrum of verbenalinoside B (**2**)

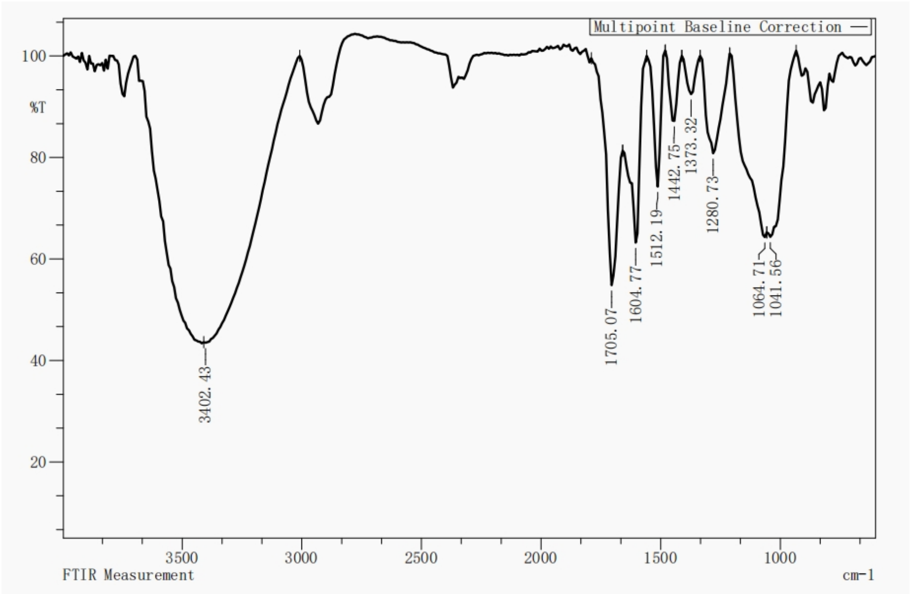


**Figure S20.** IR Value of verbenalinoside B (**2**)

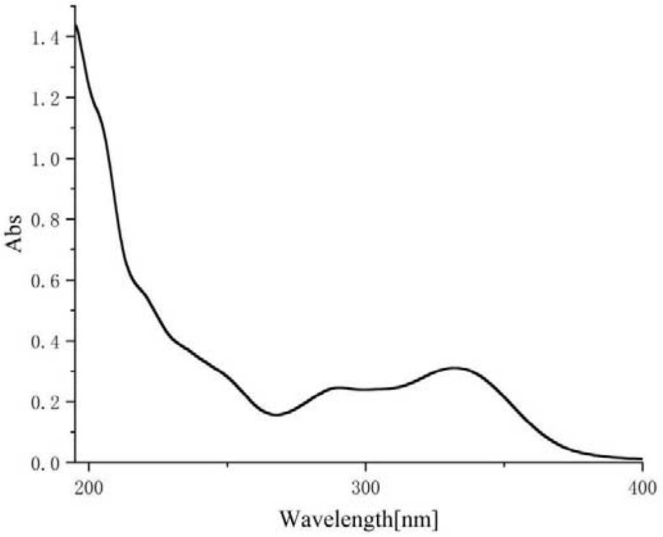


**Figure S21.** UV spectrum of verbenalinoside B (**2**)

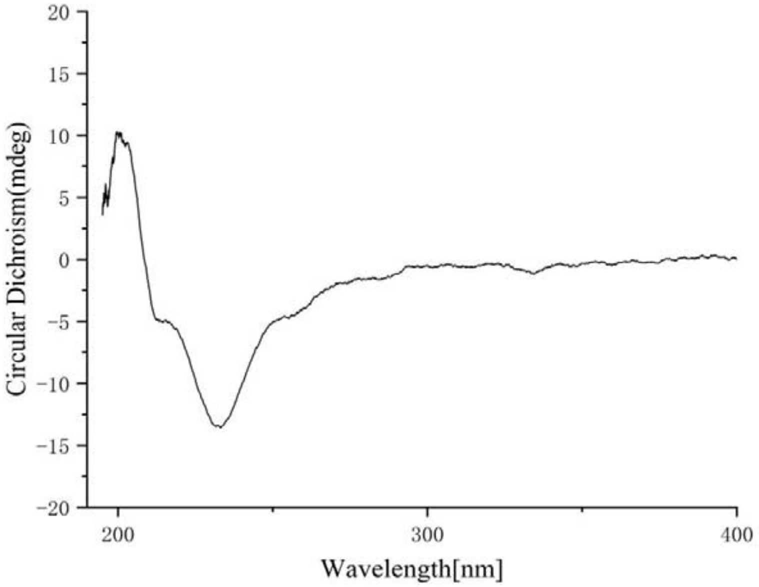


**Figure S22.** CD spectrum of verbenalinoside B (**2**)

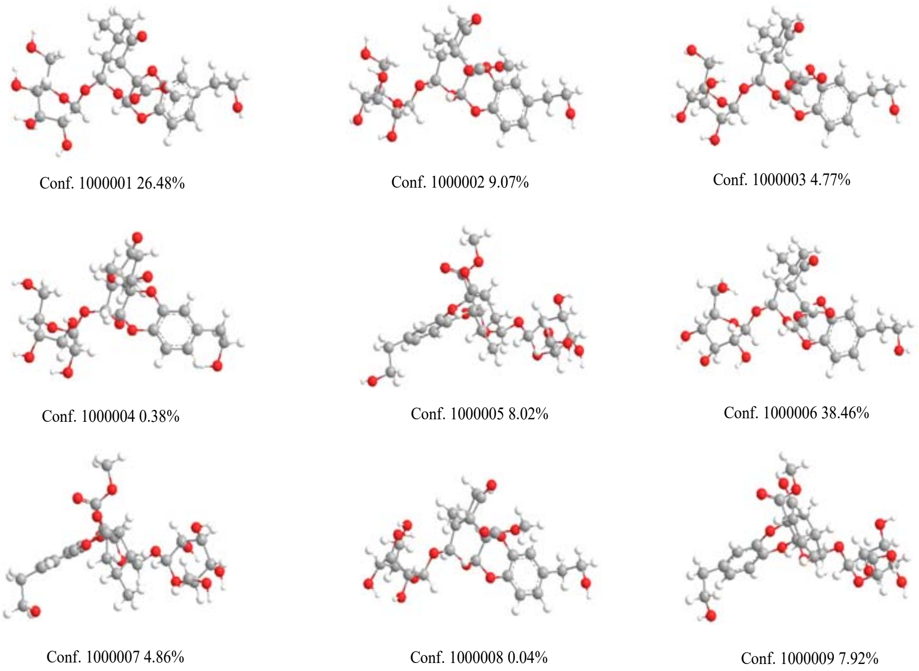


**Figure S23.** Nine lowest energy conformers of **1** ( Unit A and the phenylethanoid moiety in unit B) by DP4+ calculation.

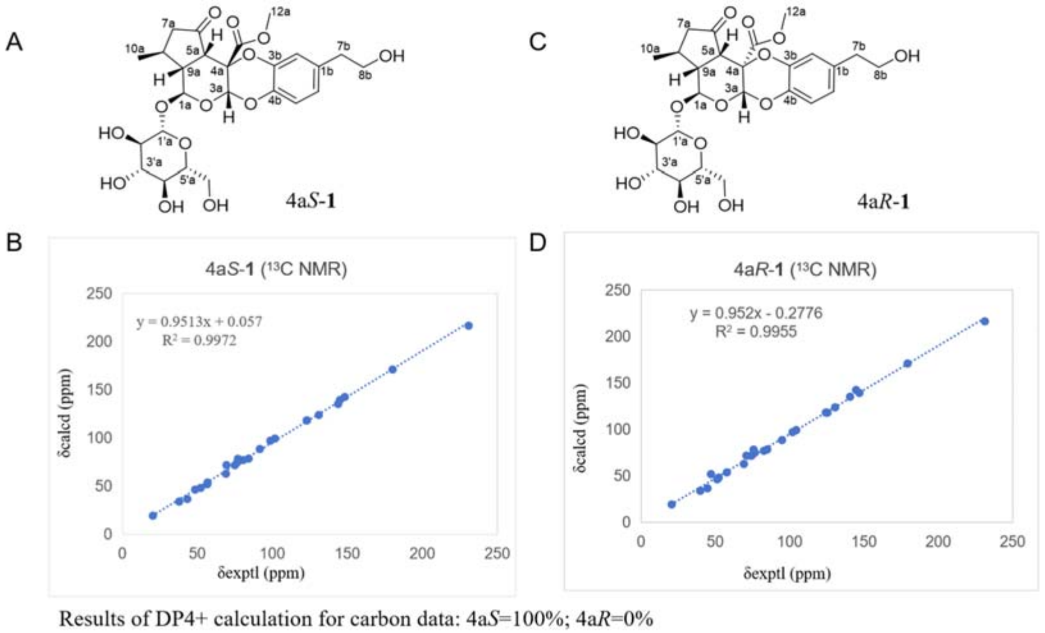


**Figure S24.** DP4 plus Calculation Results of compound **1** ( Unit A and the phenylethanoid moiety in unit B)

(A) The structure of 4a*S*-**1** isomer. (B) Regression analysis of experimental versus calculated $^{13}$C NMR chemical shifts for 4a*S*-**1**. (C) The structure of 4a*R*-**1** isomer. (D)

Regression analysis of experimental versus calculated $^{13}$C NMR chemical shifts for 4a*R*-**1**.

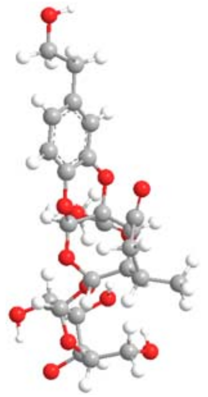

Conf. 1000001 100%

**Figure S25.** DFT computational optimized conformations of (1a*R*,3a*R*,4a*S*,5a*S*,10a*S*,1'a*S*,2'a*R*,3'a*S*,4'a*S*,5'a*R*) **- 1** (Unit A and the phenylethanoid moiety in unit B)

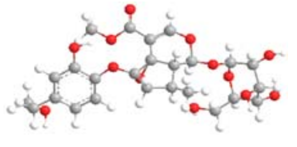

Conf. 2000001 6.57%

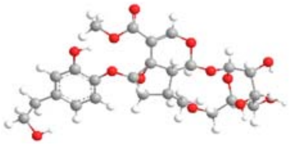

Conf. 2000002 8.92%

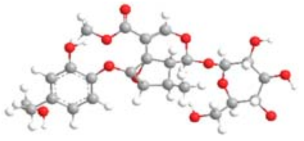

Conf. 2000003 23.36%

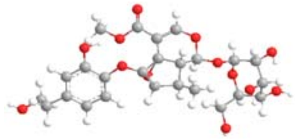

Conf. 2000004 4.48%

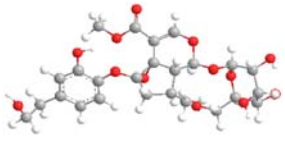

Conf. 2000005 13.04%

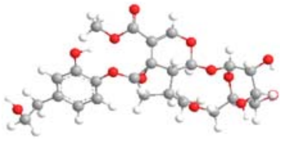

Conf. 2000006 13.38%

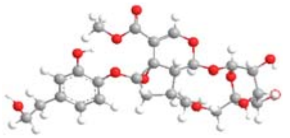

Conf. 2000007 12.98%

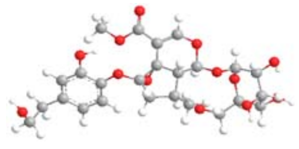

Conf. 2000008 15.9%

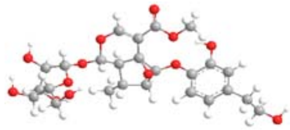

Conf. 2000009 1.36%

**Figure S26.** Nine lowest energy conformers of **2** (Unit A and the phenylethanoid moiety in unit B) by DP4+ calculation.

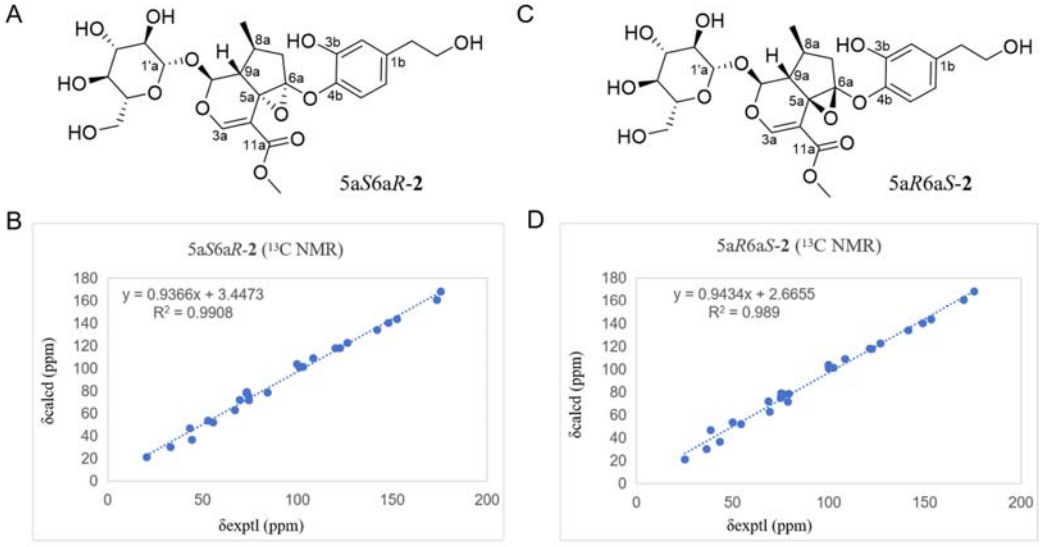


Results of DP4+ calculation for carbon data: 5a*S*6a*R*=100%; 5a*R*6a*S*=0%

**Figure S27.** DP4 plus Calculation Results of compound **2** (Unit A and phenylethanoid moiety in unit B)

(A) The structure of 5a*S*6a*R*-**2** isomer. (B) Regression analysis of experimental versus calculated $^{13}$C NMR chemical shifts for 5a*S*6a*R*-**2**. (C) The structure of 5a*R*6a*S*-**2** isomer. (D) Regression analysis of experimental versus calculated $^{13}$C NMR chemical shifts for 5a*R*6aS-**2**.

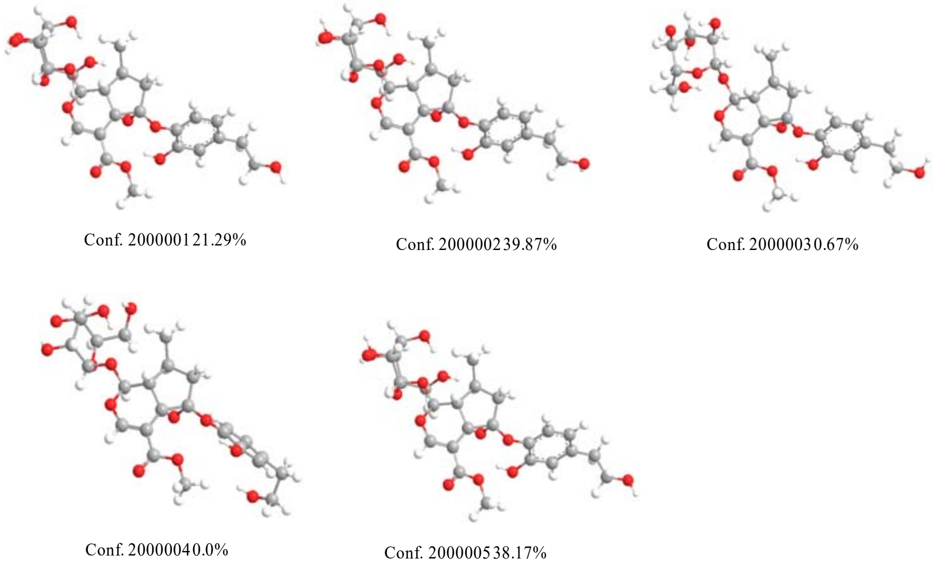


**Figure S28.** DFT computational optimized conformations of (1a*S*,5a*S*,6a*R*,8a*S*,9a*R*,1'a*S*,2'a*R*,3'a*S*,4'a*S*,5'a*R*) - **2** (Unit A and the phenylethanoid moiety in unit B)

**Table S3.** Cartesian coordinates for the low-energy reoptimized random research conformers of compound **1** (Unit A and the phenylethanoid moiety in unit B) (obtained by DP4+ calculation)

| Compound**1**-DP4+000001_en_ | | Standard Orientation (A.U.) | | | |
|---|---|---|---|---|---|
| Center number | Atomic number | Atomic Type | X | Y | Z |
| 0 | 6 | 0 | 7.682854 | 0.84947 | -1.928282 |
| 1 | 6 | 0 | 5.507201 | -0.09217 | -0.793694 |
| 2 | 6 | 0 | 4.874055 | -2.641608 | -1.049618 |
| 3 | 6 | 0 | 6.446455 | -4.249782 | -2.408426 |
| 4 | 6 | 0 | 8.641037 | -3.305106 | -3.518283 |
| 5 | 6 | 0 | 9.278406 | -0.743226 | -3.30864 |
| 6 | 6 | 0 | 11.69855 | 0.267228 | -4.428838 |
| 7 | 6 | 0 | 13.941591 | 0.109648 | -2.590951 |
| 8 | 8 | 0 | 14.501539 | -2.406952 | -1.817743 |
| 9 | 6 | 0 | 0.896864 | -1.800397 | 0.69143 |
| 10 | 6 | 0 | 2.112727 | 0.438787 | 2.104153 |
| 11 | 6 | 0 | 0.10295 | 2.484236 | 2.626655 |
| 12 | 6 | 0 | -1.584543 | 3.070455 | 0.331864 |
| 13 | 6 | 0 | -2.279803 | 0.743337 | -1.217184 |
| 14 | 8 | 0 | -0.205126 | -0.922383 | -1.582169 |
| 15 | 6 | 0 | 1.47629 | 4.990496 | 3.153949 |
| 16 | 6 | 0 | 1.007725 | 6.7842 | 0.961309 |
| 17 | 6 | 0 | -0.18572 | 5.173569 | -1.150346 |
| 18 | 8 | 0 | 2.734078 | 5.394805 | 5.028924 |
| 19 | 6 | 0 | -1.896966 | 6.670304 | -2.927112 |
| 20 | 8 | 0 | -4.267652 | -0.503899 | 0.073563 |
| 21 | 8 | 0 | 2.712502 | -3.618782 | 0.057718 |
| 22 | 8 | 0 | 3.999411 | 1.554697 | 0.539761 |
| 23 | 6 | 0 | 3.185441 | -0.525542 | 4.619556 |
| 24 | 8 | 0 | 5.67738 | -0.25811 | 4.792884 |
| 25 | 8 | 0 | 1.83581 | -1.466571 | 6.222585 |
| 26 | 6 | 0 | 6.808997 | -1.177107 | 7.101247 |
| 27 | 1 | 0 | -3.35879 | 3.863842 | 1.047727 |
| 28 | 1 | 0 | -0.972931 | 1.885176 | 4.281715 |
| 29 | 6 | 0 | -5.47166 | -2.428869 | -1.373661 |
| 30 | 6 | 0 | -7.013839 | -4.040546 | 0.495928 |
| 31 | 6 | 0 | -9.82319 | -3.419686 | 0.326741 |
| 32 | 6 | 0 | -10.139705 | -0.595404 | -0.078824 |
| 33 | 6 | 0 | -9.08589 | 0.077346 | -2.698461 |

| | | | | | |
|---|---|---|---|---|---|
| 34 | 8 | 0 | -6.892791 | -1.388049 | -3.369916 |
| 35 | 8 | 0 | -6.513455 | -6.620793 | -0.071783 |
| 36 | 8 | 0 | -12.758713 | -0.021713 | 0.077721 |
| 37 | 8 | 0 | -11.004932 | -4.253452 | 2.58856 |
| 38 | 6 | 0 | -8.535592 | 2.896599 | -2.942574 |
| 39 | 8 | 0 | -10.892797 | 4.163188 | -2.525286 |
| 40 | 1 | 0 | -0.490012 | -2.7444 | 1.888381 |
| 41 | 1 | 0 | 8.116436 | 2.842834 | -1.699863 |
| 42 | 1 | 0 | 5.922077 | -6.224758 | -2.585091 |
| 43 | 1 | 0 | 9.864826 | -4.571862 | -4.569628 |
| 44 | 1 | 0 | 12.178232 | -0.782326 | -6.153186 |
| 45 | 1 | 0 | 11.463147 | 2.251276 | -4.981388 |
| 46 | 1 | 0 | 15.603208 | 1.012292 | -3.455383 |
| 47 | 1 | 0 | 13.494591 | 1.140867 | -0.853945 |
| 48 | 1 | 0 | 14.998475 | -3.339751 | -3.316745 |
| 49 | 1 | 0 | -2.87296 | 1.236486 | -3.132304 |
| 50 | 1 | 0 | 2.747461 | 7.760408 | 0.421995 |
| 51 | 1 | 0 | -0.332968 | 8.232462 | 1.616016 |
| 52 | 1 | 0 | 1.334079 | 4.29126 | -2.242349 |
| 53 | 1 | 0 | -2.722663 | 5.45974 | -4.389989 |
| 54 | 1 | 0 | -3.449457 | 7.560002 | -1.880115 |
| 55 | 1 | 0 | -0.835032 | 8.166164 | -3.885341 |
| 56 | 1 | 0 | 6.051188 | -0.140187 | 8.715986 |
| 57 | 1 | 0 | 6.426743 | -3.192314 | 7.322604 |
| 58 | 1 | 0 | 8.82589 | -0.832729 | 6.890913 |
| 59 | 1 | 0 | -4.055022 | -3.61391 | -2.294827 |
| 60 | 1 | 0 | -6.372017 | -3.573568 | 2.412617 |
| 61 | 1 | 0 | -10.616716 | -4.41529 | -1.319429 |
| 62 | 1 | 0 | -9.090533 | 0.426366 | 1.397989 |
| 63 | 1 | 0 | -10.537952 | -0.436689 | -4.082067 |
| 64 | 1 | 0 | -7.537107 | -7.619041 | 1.079538 |
| 65 | 1 | 0 | -12.889414 | 1.759713 | -0.389501 |
| 66 | 1 | 0 | -12.698092 | -3.538053 | 2.56004 |
| 67 | 1 | 0 | -7.779462 | 3.317444 | -4.828456 |
| 68 | 1 | 0 | -7.135011 | 3.461379 | -1.525377 |
| 69 | 1 | 0 | -10.547669 | 5.923908 | -2.156701 |

| Compound**1**-DP4+000002_en_ | | Standard Orientation (A.U.) | | | |
|---|---|---|---|---|---|
| Center number | Atomic number | Atomic Type | X | Y | Z |
| 0 | 6 | 0 | 7.659905 | -0.064644 | -2.097968 |

| | | | | | |
|---|---|---|---|---|---|
| 1 | 6 | 0 | 5.485593 | -0.418075 | -0.663224 |
| 2 | 6 | 0 | 4.827381 | -2.832312 | 0.174278 |
| 3 | 6 | 0 | 6.372918 | -4.880579 | -0.394554 |
| 4 | 6 | 0 | 8.56433 | -4.515283 | -1.809553 |
| 5 | 6 | 0 | 9.227066 | -2.107785 | -2.694731 |
| 6 | 6 | 0 | 11.638742 | -1.691004 | -4.159059 |
| 7 | 6 | 0 | 13.957867 | -1.335171 | -2.447316 |
| 8 | 8 | 0 | 14.479632 | -3.482329 | -0.909446 |
| 9 | 6 | 0 | 0.865351 | -1.297845 | 1.394382 |
| 10 | 6 | 0 | 2.103305 | 1.317515 | 1.739058 |
| 11 | 6 | 0 | 0.113994 | 3.410922 | 1.339164 |
| 12 | 6 | 0 | -1.570104 | 2.986145 | -0.993193 |
| 13 | 6 | 0 | -2.295541 | 0.229492 | -1.409084 |
| 14 | 8 | 0 | -0.227206 | -1.451262 | -1.039378 |
| 15 | 6 | 0 | 1.514795 | 5.889657 | 0.758976 |
| 16 | 6 | 0 | 1.071545 | 6.584233 | -1.991754 |
| 17 | 6 | 0 | -0.149842 | 4.245551 | -3.223359 |
| 18 | 8 | 0 | 2.773731 | 7.043011 | 2.291081 |
| 19 | 6 | 0 | -1.853663 | 4.871693 | -5.466086 |
| 20 | 8 | 0 | -4.282698 | -0.337945 | 0.286154 |
| 21 | 8 | 0 | 2.665975 | -3.229217 | 1.596392 |
| 22 | 8 | 0 | 4.005388 | 1.65447 | -0.138041 |
| 23 | 6 | 0 | 3.161982 | 1.488482 | 4.4325 |
| 24 | 8 | 0 | 5.659399 | 1.759588 | 4.494659 |
| 25 | 8 | 0 | 1.79846 | 1.332775 | 6.274684 |
| 26 | 6 | 0 | 6.775231 | 1.867626 | 6.983851 |
| 27 | 1 | 0 | -3.33494 | 4.026135 | -0.692171 |
| 28 | 1 | 0 | -0.96631 | 3.579447 | 3.088612 |
| 29 | 6 | 0 | -5.561113 | -2.633067 | -0.271663 |
| 30 | 6 | 0 | -7.113967 | -3.422209 | 2.047133 |
| 31 | 6 | 0 | -9.5299 | -1.843847 | 2.373262 |
| 32 | 6 | 0 | -10.967679 | -1.688947 | -0.15244 |
| 33 | 6 | 0 | -9.26539 | -0.920017 | -2.366374 |
| 34 | 8 | 0 | -7.023086 | -2.442556 | -2.494461 |
| 35 | 8 | 0 | -7.736947 | -6.026231 | 1.835491 |
| 36 | 8 | 0 | -11.983524 | -4.180533 | -0.523254 |
| 37 | 8 | 0 | -8.859482 | 0.58135 | 3.309672 |
| 38 | 6 | 0 | -8.691563 | 1.917575 | -2.457396 |
| 39 | 8 | 0 | -11.018466 | 3.13457 | -3.079473 |
| 40 | 1 | 0 | -0.529265 | -1.635382 | 2.87357 |
| 41 | 1 | 0 | 8.113071 | 1.835631 | -2.728077 |
| 42 | 1 | 0 | 5.828237 | -6.741781 | 0.273489 |

| | | | | | |
|---|---|---|---|---|---|
| 43 | 1 | 0 | 9.766713 | -6.119738 | -2.239025 |
| 44 | 1 | 0 | 11.993931 | -3.2961 | -5.425544 |
| 45 | 1 | 0 | 11.465269 | -0.010188 | -5.359226 |
| 46 | 1 | 0 | 15.604194 | -0.839403 | -3.615991 |
| 47 | 1 | 0 | 13.622541 | 0.22661 | -1.132605 |
| 48 | 1 | 0 | 14.913235 | -4.864563 | -2.034562 |
| 49 | 1 | 0 | -2.892887 | -0.126234 | -3.352981 |
| 50 | 1 | 0 | 2.826569 | 7.211274 | -2.88493 |
| 51 | 1 | 0 | -0.243605 | 8.194397 | -2.024422 |
| 52 | 1 | 0 | 1.354367 | 2.96509 | -3.838831 |
| 53 | 1 | 0 | -2.70208 | 3.165837 | -6.277636 |
| 54 | 1 | 0 | -3.39053 | 6.13843 | -4.890538 |
| 55 | 1 | 0 | -0.782209 | 5.808788 | -6.968762 |
| 56 | 1 | 0 | 8.797561 | 2.064017 | 6.663787 |
| 57 | 1 | 0 | 6.033682 | 3.492204 | 8.017048 |
| 58 | 1 | 0 | 6.360463 | 0.132315 | 8.019814 |
| 59 | 1 | 0 | -4.184092 | -4.116474 | -0.68293 |
| 60 | 1 | 0 | -5.928041 | -3.199548 | 3.724548 |
| 61 | 1 | 0 | -10.737571 | -2.851536 | 3.727434 |
| 62 | 1 | 0 | -12.504492 | -0.303898 | -0.006971 |
| 63 | 1 | 0 | -10.268216 | -1.364418 | -4.125548 |
| 64 | 1 | 0 | -9.153238 | -6.105227 | 0.648958 |
| 65 | 1 | 0 | -12.668681 | -4.288267 | -2.222025 |
| 66 | 1 | 0 | -10.414334 | 1.498201 | 3.639083 |
| 67 | 1 | 0 | -7.256036 | 2.240257 | -3.924579 |
| 68 | 1 | 0 | -7.953245 | 2.573265 | -0.646422 |
| 69 | 1 | 0 | -10.826936 | 4.916659 | -2.704315 |

| Compound**1**-DP4+000003_en_ | | Standard Orientation (A.U.) | | | |
|---|---|---|---|---|---|
| Center number | Atomic number | Atomic Type | X | Y | Z |
| 0 | 6 | 0 | 7.835268 | 0.4269 | -1.970306 |
| 1 | 6 | 0 | 5.627533 | -0.244124 | -0.711633 |
| 2 | 6 | 0 | 4.986613 | -2.787603 | -0.423336 |
| 3 | 6 | 0 | 6.582861 | -4.654137 | -1.357262 |
| 4 | 6 | 0 | 8.808711 | -3.974899 | -2.591621 |
| 5 | 6 | 0 | 9.455557 | -1.428648 | -2.928817 |
| 6 | 6 | 0 | 11.90608 | -0.686677 | -4.184719 |
| 7 | 6 | 0 | 14.12767 | -0.523792 | -2.320797 |
| 8 | 8 | 0 | 14.657222 | -2.860773 | -1.094473 |
| 9 | 6 | 0 | 0.971894 | -1.590934 | 0.995164 |

| | | | | | |
|---|---|---|---|---|---|
| 10 | 6 | 0 | 2.164489 | 0.907645 | 1.903138 |
| 11 | 6 | 0 | 0.15484 | 3.021287 | 1.91728 |
| 12 | 6 | 0 | -1.473735 | 3.080331 | -0.493175 |
| 13 | 6 | 0 | -2.149998 | 0.466335 | -1.491729 |
| 14 | 8 | 0 | -0.088044 | -1.238674 | -1.441634 |
| 15 | 6 | 0 | 1.526617 | 5.58447 | 1.898627 |
| 16 | 6 | 0 | 1.142345 | 6.8257 | -0.662173 |
| 17 | 6 | 0 | -0.019198 | 4.783781 | -2.381779 |
| 18 | 8 | 0 | 2.71449 | 6.416255 | 3.674773 |
| 19 | 6 | 0 | -1.677669 | 5.84635 | -4.490069 |
| 20 | 8 | 0 | -4.166891 | -0.477031 | 0.017962 |
| 21 | 8 | 0 | 2.790449 | -3.500863 | 0.81324 |
| 22 | 8 | 0 | 4.08525 | 1.653525 | 0.174471 |
| 23 | 6 | 0 | 3.343644 | 0.541034 | 4.531576 |
| 24 | 8 | 0 | 1.556858 | -0.058695 | 6.219871 |
| 25 | 8 | 0 | 5.577544 | 0.713256 | 4.983673 |
| 26 | 6 | 0 | 2.427795 | -0.514108 | 8.76776 |
| 27 | 1 | 0 | -3.254613 | 4.029299 | -0.028652 |
| 28 | 1 | 0 | -0.968369 | 2.821981 | 3.635206 |
| 29 | 6 | 0 | -5.428835 | -2.644352 | -1.005081 |
| 30 | 6 | 0 | -6.947633 | -3.828232 | 1.162909 |
| 31 | 6 | 0 | -9.131092 | -2.063435 | 1.928082 |
| 32 | 6 | 0 | -10.724899 | -1.400595 | -0.420793 |
| 33 | 6 | 0 | -9.132241 | -0.473923 | -2.658697 |
| 34 | 8 | 0 | -6.923696 | -2.00499 | -3.102866 |
| 35 | 8 | 0 | -7.823559 | -6.251254 | 0.438443 |
| 36 | 8 | 0 | -12.037064 | -3.700165 | -1.039729 |
| 37 | 8 | 0 | -8.264128 | 0.144027 | 3.194505 |
| 38 | 6 | 0 | -8.507366 | 2.36527 | -2.524321 |
| 39 | 8 | 0 | -10.735657 | 3.842439 | -2.799688 |
| 40 | 1 | 0 | -0.446714 | -2.254234 | 2.33378 |
| 41 | 1 | 0 | 8.273419 | 2.42195 | -2.17299 |
| 42 | 1 | 0 | 6.05151 | -6.619915 | -1.112266 |
| 43 | 1 | 0 | 10.05116 | -5.440437 | -3.309358 |
| 44 | 1 | 0 | 12.387419 | -2.050856 | -5.672036 |
| 45 | 1 | 0 | 11.709828 | 1.159317 | -5.106936 |
| 46 | 1 | 0 | 15.806614 | 0.193266 | -3.31557 |
| 47 | 1 | 0 | 13.672025 | 0.810832 | -0.807153 |
| 48 | 1 | 0 | 15.179535 | -4.049706 | -2.389852 |
| 49 | 1 | 0 | -2.734759 | 0.51661 | -3.470112 |
| 50 | 1 | 0 | 2.911199 | 7.633696 | -1.361276 |
| 51 | 1 | 0 | -0.190437 | 8.399377 | -0.397651 |

| | | | | | |
|---|---|---|---|---|---|
| 52 | 1 | 0 | 1.514549 | 3.669276 | -3.209046 |
| 53 | 1 | 0 | -2.476962 | 4.338979 | -5.663526 |
| 54 | 1 | 0 | -3.247593 | 6.9508 | -3.707714 |
| 55 | 1 | 0 | -0.581905 | 7.085507 | -5.733873 |
| 56 | 1 | 0 | 3.316779 | 1.188424 | 9.520913 |
| 57 | 1 | 0 | 0.741338 | -0.994884 | 9.842733 |
| 58 | 1 | 0 | 3.778199 | -2.073602 | 8.782593 |
| 59 | 1 | 0 | -4.034339 | -3.984667 | -1.723665 |
| 60 | 1 | 0 | -5.67937 | -4.081393 | 2.776896 |
| 61 | 1 | 0 | -10.361782 | -3.08354 | 3.24072 |
| 62 | 1 | 0 | -12.076769 | 0.090747 | 0.064991 |
| 63 | 1 | 0 | -10.248542 | -0.741925 | -4.385714 |
| 64 | 1 | 0 | -9.342432 | -5.962173 | -0.577822 |
| 65 | 1 | 0 | -12.950189 | -3.452656 | -2.611734 |
| 66 | 1 | 0 | -6.596405 | 0.543727 | 2.52626 |
| 67 | 1 | 0 | -7.104178 | 2.801256 | -3.989458 |
| 68 | 1 | 0 | -7.721612 | 2.876726 | -0.693014 |
| 69 | 1 | 0 | -11.33425 | 3.639868 | -4.522402 |

| Compound**1**-DP4+000004_en_ | | Standard Orientation (A.U.) | | | |
|---|---|---|---|---|---|
| Center number | Atomic number | Atomic Type | X | Y | Z |
| 0 | 6 | 0 | 7.979102 | -0.003065 | 0.319364 |
| 1 | 6 | 0 | 5.403494 | 0.004492 | 0.838581 |
| 2 | 6 | 0 | 4.284601 | -2.065172 | 2.044449 |
| 3 | 6 | 0 | 5.763359 | -4.108197 | 2.774205 |
| 4 | 6 | 0 | 8.35439 | -4.096314 | 2.281045 |
| 5 | 6 | 0 | 9.493289 | -2.049298 | 1.038618 |
| 6 | 6 | 0 | 12.262323 | -2.114634 | 0.351663 |
| 7 | 6 | 0 | 12.694279 | -3.390698 | -2.22348 |
| 8 | 8 | 0 | 11.881322 | -5.955701 | -2.257168 |
| 9 | 6 | 0 | 0.289215 | -0.412903 | 1.112266 |
| 10 | 6 | 0 | 1.506779 | 2.228426 | 1.083208 |
| 11 | 6 | 0 | -0.006514 | 3.990159 | -0.681375 |
| 12 | 6 | 0 | -0.692443 | 2.763161 | -3.229213 |
| 13 | 6 | 0 | -1.439531 | -0.015505 | -3.015572 |
| 14 | 8 | 0 | 0.209811 | -1.384848 | -1.384597 |
| 15 | 6 | 0 | 1.698337 | 6.225824 | -1.412272 |
| 16 | 6 | 0 | 2.433606 | 5.967409 | -4.175894 |
| 17 | 6 | 0 | 1.58614 | 3.303052 | -4.989792 |
| 18 | 8 | 0 | 2.329176 | 7.889098 | 0.036237 |

| | | | | | |
|---|---|---|---|---|---|
| 19 | 6 | 0 | 0.953858 | 3.098081 | -7.797292 |
| 20 | 8 | 0 | -3.950661 | -0.09929 | -2.154717 |
| 21 | 8 | 0 | 1.726099 | -2.09757 | 2.574432 |
| 22 | 8 | 0 | 4.005254 | 2.059656 | 0.090495 |
| 23 | 6 | 0 | 1.639797 | 3.274984 | 3.786437 |
| 24 | 8 | 0 | -0.688977 | 3.604112 | 4.720209 |
| 25 | 8 | 0 | 3.581509 | 3.673913 | 4.926569 |
| 26 | 6 | 0 | -0.82856 | 4.510285 | 7.2908 |
| 27 | 1 | 0 | -2.341237 | 3.759255 | -3.989518 |
| 28 | 1 | 0 | -1.660833 | 4.654446 | 0.354855 |
| 29 | 6 | 0 | -5.295765 | -2.403647 | -2.56539 |
| 30 | 6 | 0 | -5.102557 | -4.24942 | -0.324969 |
| 31 | 6 | 0 | -6.648992 | -3.378672 | 1.973942 |
| 32 | 6 | 0 | -9.34589 | -2.660113 | 1.148107 |
| 33 | 6 | 0 | -9.372853 | -0.892314 | -1.145773 |
| 34 | 8 | 0 | -7.80746 | -1.816407 | -3.165202 |
| 35 | 8 | 0 | -5.935696 | -6.674915 | -1.129315 |
| 36 | 8 | 0 | -10.549069 | -5.02724 | 0.57616 |
| 37 | 8 | 0 | -5.38064 | -1.317283 | 3.143862 |
| 38 | 6 | 0 | -8.811385 | 1.883645 | -0.477685 |
| 39 | 8 | 0 | -10.727739 | 2.87989 | 1.132972 |
| 40 | 1 | 0 | -1.587294 | -0.357884 | 1.957921 |
| 41 | 1 | 0 | 8.786429 | 1.625314 | -0.633912 |
| 42 | 1 | 0 | 4.863172 | -5.689414 | 3.72054 |
| 43 | 1 | 0 | 9.503361 | -5.688166 | 2.878125 |
| 44 | 1 | 0 | 13.03295 | -0.193219 | 0.269976 |
| 45 | 1 | 0 | 13.329429 | -3.156895 | 1.788316 |
| 46 | 1 | 0 | 11.741577 | -2.289192 | -3.709136 |
| 47 | 1 | 0 | 14.71573 | -3.427882 | -2.657996 |
| 48 | 1 | 0 | 10.09099 | -5.949251 | -1.845378 |
| 49 | 1 | 0 | -1.266918 | -0.993875 | -4.831691 |
| 50 | 1 | 0 | 4.447824 | 6.348227 | -4.4395 |
| 51 | 1 | 0 | 1.390285 | 7.419607 | -5.236635 |
| 52 | 1 | 0 | 3.092338 | 1.963038 | -4.525448 |
| 53 | 1 | 0 | 2.606985 | 3.548615 | -8.95863 |
| 54 | 1 | 0 | 0.339075 | 1.186754 | -8.303236 |
| 55 | 1 | 0 | -0.564295 | 4.414088 | -8.30748 |
| 56 | 1 | 0 | 0.118459 | 3.186284 | 8.558273 |
| 57 | 1 | 0 | 0.057478 | 6.368203 | 7.431875 |
| 58 | 1 | 0 | -2.837144 | 4.624604 | 7.719909 |
| 59 | 1 | 0 | -4.534262 | -3.347221 | -4.244474 |
| 60 | 1 | 0 | -3.116319 | -4.434184 | 0.203062 |

| | | | | | |
|---|---|---|---|---|---|
| 61 | 1 | 0 | -6.785717 | -4.99002 | 3.272339 |
| 62 | 1 | 0 | -10.335583 | -1.711661 | 2.704098 |
| 63 | 1 | 0 | -11.280947 | -0.943019 | -1.960551 |
| 64 | 1 | 0 | -7.783272 | -6.599415 | -1.129618 |
| 65 | 1 | 0 | -12.176745 | -4.68411 | -0.198474 |
| 66 | 1 | 0 | -6.213867 | -0.957898 | 4.737638 |
| 67 | 1 | 0 | -8.643086 | 2.95865 | -2.243878 |
| 68 | 1 | 0 | -7.041581 | 2.042168 | 0.555844 |
| 69 | 1 | 0 | -12.27886 | 2.989333 | 0.159446 |

| Compound**1**-DP4+000005_en_ | | Standard Orientation (A.U.) | | | |
|---|---|---|---|---|---|
| Center number | Atomic number | Atomic Type | X | Y | Z |
| 0 | 6 | 0 | -8.100477 | -0.68735 | 0.120835 |
| 1 | 6 | 0 | -5.749225 | 0.224263 | -0.619923 |
| 2 | 6 | 0 | -4.962179 | -0.020148 | -3.128761 |
| 3 | 6 | 0 | -6.555978 | -1.127911 | -4.899344 |
| 4 | 6 | 0 | -8.924222 | -2.01212 | -4.156071 |
| 5 | 6 | 0 | -9.721505 | -1.814455 | -1.637901 |
| 6 | 6 | 0 | -12.222669 | -2.902045 | -0.801346 |
| 7 | 6 | 0 | -11.953427 | -5.668719 | 0.038966 |
| 8 | 8 | 0 | -14.267825 | -6.711835 | 0.933107 |
| 9 | 6 | 0 | -0.886843 | 1.207112 | -1.938581 |
| 10 | 6 | 0 | -2.093771 | 2.678271 | 0.268564 |
| 11 | 6 | 0 | -0.197291 | 2.887148 | 2.473602 |
| 12 | 6 | 0 | 1.158706 | 0.383172 | 3.07216 |
| 13 | 6 | 0 | 1.847665 | -1.149798 | 0.732635 |
| 14 | 8 | 0 | -0.134913 | -1.21191 | -1.085328 |
| 15 | 6 | 0 | -1.682533 | 3.480823 | 4.89986 |
| 16 | 6 | 0 | -1.634413 | 1.173034 | 6.608238 |
| 17 | 6 | 0 | -0.562942 | -1.000946 | 4.995189 |
| 18 | 8 | 0 | -2.713964 | 5.486754 | 5.316195 |
| 19 | 6 | 0 | 0.817014 | -3.015699 | 6.531973 |
| 20 | 8 | 0 | 4.05197 | -0.060362 | -0.316291 |
| 21 | 8 | 0 | -2.626362 | 0.867284 | -3.906328 |
| 22 | 8 | 0 | -4.225156 | 1.311513 | 1.182135 |
| 23 | 6 | 0 | -2.968418 | 5.291103 | -0.644535 |
| 24 | 8 | 0 | -0.984596 | 6.667787 | -1.401345 |
| 25 | 8 | 0 | -5.149377 | 5.972285 | -0.725188 |
| 26 | 6 | 0 | -1.557575 | 9.156885 | -2.365984 |
| 27 | 1 | 0 | 2.938037 | 0.844462 | 4.025126 |

| | | | | | |
|---|---|---|---|---|---|
| 28 | 1 | 0 | 1.113786 | 4.418786 | 2.040849 |
| 29 | 6 | 0 | 5.26349 | -1.611114 | -2.144548 |
| 30 | 6 | 0 | 7.151763 | 0.008714 | -3.634949 |
| 31 | 6 | 0 | 9.5699 | 0.585617 | -2.133011 |
| 32 | 6 | 0 | 10.621433 | -1.861218 | -0.958855 |
| 33 | 6 | 0 | 8.604728 | -3.373626 | 0.464978 |
| 34 | 8 | 0 | 6.37824 | -3.767585 | -1.03954 |
| 35 | 8 | 0 | 7.76799 | -1.252178 | -5.924768 |
| 36 | 8 | 0 | 11.602968 | -3.273979 | -3.061014 |
| 37 | 8 | 0 | 9.010307 | 2.441804 | -0.276027 |
| 38 | 6 | 0 | 8.000687 | -2.302061 | 3.100585 |
| 39 | 8 | 0 | 10.160302 | -2.427157 | 4.703678 |
| 40 | 1 | 0 | 0.702761 | 2.250944 | -2.731843 |
| 41 | 1 | 0 | -8.653464 | -0.478824 | 2.086375 |
| 42 | 1 | 0 | -5.914915 | -1.2868 | -6.84105 |
| 43 | 1 | 0 | -10.162981 | -2.872091 | -5.548536 |
| 44 | 1 | 0 | -13.002542 | -1.813708 | 0.778223 |
| 45 | 1 | 0 | -13.593061 | -2.805601 | -2.355251 |
| 46 | 1 | 0 | -11.167664 | -6.797874 | -1.520544 |
| 47 | 1 | 0 | -10.625875 | -5.788526 | 1.621359 |
| 48 | 1 | 0 | -15.428472 | -6.75641 | -0.486478 |
| 49 | 1 | 0 | 2.179894 | -3.136764 | 1.183789 |
| 50 | 1 | 0 | -3.5054 | 0.810987 | 7.407938 |
| 51 | 1 | 0 | -0.360647 | 1.600031 | 8.195017 |
| 52 | 1 | 0 | -2.120998 | -1.878019 | 3.954309 |
| 53 | 1 | 0 | -0.457958 | -3.903069 | 7.899875 |
| 54 | 1 | 0 | 1.568849 | -4.515962 | 5.318572 |
| 55 | 1 | 0 | 2.401831 | -2.185572 | 7.579144 |
| 56 | 1 | 0 | 0.256572 | 9.965407 | -2.901009 |
| 57 | 1 | 0 | -2.806806 | 9.009521 | -4.001166 |
| 58 | 1 | 0 | -2.45976 | 10.289638 | -0.89652 |
| 59 | 1 | 0 | 3.859675 | -2.369299 | -3.456394 |
| 60 | 1 | 0 | 6.224346 | 1.78691 | -4.132601 |
| 61 | 1 | 0 | 10.974726 | 1.292612 | -3.487134 |
| 62 | 1 | 0 | 12.145697 | -1.409726 | 0.373308 |
| 63 | 1 | 0 | 9.338635 | -5.29424 | 0.749664 |
| 64 | 1 | 0 | 8.994471 | -2.556569 | -5.462006 |
| 65 | 1 | 0 | 12.045774 | -4.957445 | -2.480243 |
| 66 | 1 | 0 | 10.585925 | 2.901754 | 0.543789 |
| 67 | 1 | 0 | 6.39951 | -3.363361 | 3.887286 |
| 68 | 1 | 0 | 7.483091 | -0.316166 | 2.975807 |
| 69 | 1 | 0 | 10.518807 | -4.198013 | 5.023393 |

| Compound**1**-DP4+000006en_ | | Standard Orientation (A.U.) | | | |
|---|---|---|---|---|---|
| Center number | Atomic number | Atomic Type | X | Y | Z |
| 0 | 6 | 0 | 7.934508 | 0.127294 | -1.829716 |
| 1 | 6 | 0 | 5.60797 | -0.353742 | -0.70412 |
| 2 | 6 | 0 | 4.672457 | -2.817746 | -0.595477 |
| 3 | 6 | 0 | 6.09103 | -4.801751 | -1.574104 |
| 4 | 6 | 0 | 8.4348 | -4.316571 | -2.673964 |
| 5 | 6 | 0 | 9.380034 | -1.847385 | -2.828533 |
| 6 | 6 | 0 | 11.950322 | -1.319926 | -3.953664 |
| 7 | 6 | 0 | 14.08846 | -1.471964 | -2.014835 |
| 8 | 8 | 0 | 14.275108 | -4.01686 | -1.15077 |
| 9 | 6 | 0 | 0.752139 | -1.249901 | 0.732235 |
| 10 | 6 | 0 | 2.16985 | 1.049949 | 1.826645 |
| 11 | 6 | 0 | 0.419653 | 3.386391 | 1.868111 |
| 12 | 6 | 0 | -1.07715 | 3.750292 | -0.60108 |
| 13 | 6 | 0 | -2.011585 | 1.276663 | -1.73405 |
| 14 | 8 | 0 | -0.148243 | -0.643998 | -1.722659 |
| 15 | 6 | 0 | 2.08269 | 5.765462 | 2.030909 |
| 16 | 6 | 0 | 1.98291 | 7.14452 | -0.486858 |
| 17 | 6 | 0 | 0.655172 | 5.339707 | -2.346437 |
| 18 | 8 | 0 | 3.263277 | 6.381853 | 3.897626 |
| 19 | 6 | 0 | -0.770364 | 6.687493 | -4.46171 |
| 20 | 8 | 0 | -4.13154 | 0.502694 | -0.267052 |
| 21 | 8 | 0 | 2.355929 | -3.339399 | 0.508045 |
| 22 | 8 | 0 | 4.246846 | 1.656827 | 0.227997 |
| 23 | 6 | 0 | 3.168058 | 0.422526 | 4.480272 |
| 24 | 8 | 0 | 1.243421 | -0.060643 | 6.050544 |
| 25 | 8 | 0 | 5.381538 | 0.324765 | 5.04266 |
| 26 | 6 | 0 | 1.929488 | -0.737176 | 8.605444 |
| 27 | 1 | 0 | -2.755094 | 4.887159 | -0.167706 |
| 28 | 1 | 0 | -0.797432 | 3.243798 | 3.526331 |
| 29 | 6 | 0 | -5.510317 | -1.586382 | -1.29122 |
| 30 | 6 | 0 | -6.955163 | -2.805631 | 0.897431 |
| 31 | 6 | 0 | -9.486333 | -3.759784 | -0.088621 |
| 32 | 6 | 0 | -11.066913 | -1.522526 | -0.992429 |
| 33 | 6 | 0 | -9.514329 | 0.191718 | -2.776533 |
| 34 | 8 | 0 | -7.049364 | -0.858208 | -3.32211 |
| 35 | 8 | 0 | -5.440406 | -4.787253 | 1.878078 |
| 36 | 8 | 0 | -13.255064 | -2.351116 | -2.31853 |

| | | | | | |
|---|---|---|---|---|---|
| 37 | 8 | 0 | -10.777947 | -5.232818 | 1.768459 |
| 38 | 6 | 0 | -9.366938 | 2.93788 | -1.88487 |
| 39 | 8 | 0 | -8.483863 | 3.242917 | 0.632378 |
| 40 | 1 | 0 | -0.793793 | -1.831657 | 1.963308 |
| 41 | 1 | 0 | 8.601948 | 2.067323 | -1.896994 |
| 42 | 1 | 0 | 5.33094 | -6.703864 | -1.468204 |
| 43 | 1 | 0 | 9.544156 | -5.870531 | -3.421215 |
| 44 | 1 | 0 | 12.356507 | -2.668881 | -5.47277 |
| 45 | 1 | 0 | 11.985883 | 0.575008 | -4.789719 |
| 46 | 1 | 0 | 15.865124 | -0.858268 | -2.905369 |
| 47 | 1 | 0 | 13.688214 | -0.176079 | -0.438772 |
| 48 | 1 | 0 | 15.530013 | -4.055428 | 0.183749 |
| 49 | 1 | 0 | -2.558377 | 1.475742 | -3.714686 |
| 50 | 1 | 0 | 3.873115 | 7.741225 | -1.071777 |
| 51 | 1 | 0 | 0.853406 | 8.867593 | -0.209264 |
| 52 | 1 | 0 | 2.075004 | 4.079573 | -3.167638 |
| 53 | 1 | 0 | -1.707991 | 5.340922 | -5.724774 |
| 54 | 1 | 0 | -2.218259 | 7.950868 | -3.684384 |
| 55 | 1 | 0 | 0.521058 | 7.825443 | -5.611003 |
| 56 | 1 | 0 | 3.094241 | -2.439929 | 8.595102 |
| 57 | 1 | 0 | 2.964822 | 0.813136 | 9.489112 |
| 58 | 1 | 0 | 0.148264 | -1.074443 | 9.577283 |
| 59 | 1 | 0 | -4.17031 | -2.946612 | -2.071004 |
| 60 | 1 | 0 | -7.304665 | -1.365342 | 2.356836 |
| 61 | 1 | 0 | -9.169571 | -5.049921 | -1.672727 |
| 62 | 1 | 0 | -11.620157 | -0.417174 | 0.682123 |
| 63 | 1 | 0 | -10.455834 | 0.174042 | -4.615573 |
| 64 | 1 | 0 | -6.577245 | -5.830317 | 2.880882 |
| 65 | 1 | 0 | -14.099205 | -3.550378 | -1.210655 |
| 66 | 1 | 0 | -11.207607 | -4.126935 | 3.174944 |
| 67 | 1 | 0 | -11.280962 | 3.723851 | -1.935369 |
| 68 | 1 | 0 | -8.199552 | 4.004471 | -3.231747 |
| 69 | 1 | 0 | -6.716856 | 2.709092 | 0.637367 |

| Compound**1**-DP4+000007_en_ | | Standard Orientation (A.U.) | | | |
|---|---|---|---|---|---|
| Center number | Atomic number | Atomic Type | X | Y | Z |
| 0 | 6 | 0 | -8.182134 | -0.930067 | -0.339414 |
| 1 | 6 | 0 | -5.846773 | 0.196118 | -0.763977 |
| 2 | 6 | 0 | -5.003694 | 0.62127 | -3.229831 |
| 3 | 6 | 0 | -6.528792 | -0.037343 | -5.264611 |

| | | | | | |
|---|---|---|---|---|---|
| 4 | 6 | 0 | -8.882922 | -1.140261 | -4.830775 |
| 5 | 6 | 0 | -9.733484 | -1.614968 | -2.367993 |
| 6 | 6 | 0 | -12.228008 | -2.91844 | -1.909003 |
| 7 | 6 | 0 | -11.954205 | -5.777273 | -1.47567 |
| 8 | 8 | 0 | -10.490363 | -6.386571 | 0.700531 |
| 9 | 6 | 0 | -0.991184 | 1.610158 | -1.659325 |
| 10 | 6 | 0 | -2.286185 | 2.43148 | 0.818777 |
| 11 | 6 | 0 | -0.439395 | 2.117 | 3.053928 |
| 12 | 6 | 0 | 0.983026 | -0.42104 | 3.027617 |
| 13 | 6 | 0 | 1.770311 | -1.278175 | 0.392651 |
| 14 | 8 | 0 | -0.172277 | -0.924904 | -1.434589 |
| 15 | 6 | 0 | -1.989887 | 2.02885 | 5.509735 |
| 16 | 6 | 0 | -1.912951 | -0.641662 | 6.563429 |
| 17 | 6 | 0 | -0.733433 | -2.298092 | 4.479533 |
| 18 | 8 | 0 | -3.083228 | 3.835982 | 6.404395 |
| 19 | 6 | 0 | 0.680889 | -4.601243 | 5.489862 |
| 20 | 8 | 0 | 3.956083 | 0.108952 | -0.275341 |
| 21 | 8 | 0 | -2.681612 | 1.738176 | -3.69332 |
| 22 | 8 | 0 | -4.388343 | 0.821577 | 1.296131 |
| 23 | 6 | 0 | -3.224597 | 5.168426 | 0.586184 |
| 24 | 8 | 0 | -1.272209 | 6.741978 | 0.242293 |
| 25 | 8 | 0 | -5.422533 | 5.794919 | 0.645258 |
| 26 | 6 | 0 | -1.905522 | 9.380202 | -0.060847 |
| 27 | 1 | 0 | 2.726074 | -0.175574 | 4.117944 |
| 28 | 1 | 0 | 0.830342 | 3.741709 | 3.06328 |
| 29 | 6 | 0 | 5.246692 | -0.862137 | -2.420892 |
| 30 | 6 | 0 | 7.106816 | 1.149851 | -3.371183 |
| 31 | 6 | 0 | 9.483164 | 1.356054 | -1.714223 |
| 32 | 6 | 0 | 10.591015 | -1.290628 | -1.227131 |
| 33 | 6 | 0 | 8.600599 | -3.180662 | -0.306062 |
| 34 | 8 | 0 | 6.408477 | -3.208385 | -1.909249 |
| 35 | 8 | 0 | 7.795126 | 0.58032 | -5.903818 |
| 36 | 8 | 0 | 11.638682 | -2.054593 | -3.613886 |
| 37 | 8 | 0 | 8.840299 | 2.619644 | 0.56578 |
| 38 | 6 | 0 | 7.926235 | -2.874166 | 2.506348 |
| 39 | 8 | 0 | 10.067971 | -3.35861 | 4.064706 |
| 40 | 1 | 0 | 0.576019 | 2.867098 | -2.117158 |
| 41 | 1 | 0 | -8.770837 | -1.254852 | 1.597242 |
| 42 | 1 | 0 | -5.84833 | 0.321032 | -7.166002 |
| 43 | 1 | 0 | -10.066779 | -1.647639 | -6.429192 |
| 44 | 1 | 0 | -13.182268 | -2.091044 | -0.262345 |
| 45 | 1 | 0 | -13.485301 | -2.635236 | -3.532059 |

| | | | | | |
|---|---|---|---|---|---|
| 46 | 1 | 0 | -13.84316 | -6.638408 | -1.365698 |
| 47 | 1 | 0 | -10.960833 | -6.634101 | -3.075299 |
| 48 | 1 | 0 | -11.404142 | -5.7624 | 2.163442 |
| 49 | 1 | 0 | 2.162833 | -3.303918 | 0.32518 |
| 50 | 1 | 0 | -3.792006 | -1.242493 | 7.179669 |
| 51 | 1 | 0 | -0.695378 | -0.611018 | 8.248303 |
| 52 | 1 | 0 | -2.237519 | -2.920882 | 3.202963 |
| 53 | 1 | 0 | 2.20991 | -4.024393 | 6.765331 |
| 54 | 1 | 0 | -0.593253 | -5.847926 | 6.541763 |
| 55 | 1 | 0 | 1.514748 | -5.714777 | 3.955887 |
| 56 | 1 | 0 | -0.109712 | 10.343894 | -0.339332 |
| 57 | 1 | 0 | -3.133364 | 9.627361 | -1.700232 |
| 58 | 1 | 0 | -2.854583 | 10.07464 | 1.634296 |
| 59 | 1 | 0 | 3.890234 | -1.274299 | -3.923009 |
| 60 | 1 | 0 | 6.13252 | 2.972025 | -3.385097 |
| 61 | 1 | 0 | 10.8858 | 2.441207 | -2.792411 |
| 62 | 1 | 0 | 12.085475 | -1.17945 | 0.206753 |
| 63 | 1 | 0 | 9.386831 | -5.088171 | -0.532389 |
| 64 | 1 | 0 | 9.05395 | -0.769408 | -5.787419 |
| 65 | 1 | 0 | 12.113364 | -3.823895 | -3.507849 |
| 66 | 1 | 0 | 10.387403 | 2.860834 | 1.522395 |
| 67 | 1 | 0 | 6.352787 | -4.155043 | 2.945537 |
| 68 | 1 | 0 | 7.341707 | -0.943858 | 2.904717 |
| 69 | 1 | 0 | 10.485397 | -5.138407 | 3.90689 |

| Compound**1**-DP4+000008_en_ | | Standard Orientation (A.U.) | | | |
|---|---|---|---|---|---|
| Center number | Atomic number | Atomic Type | X | Y | Z |
| 0 | 6 | 0 | 7.508914 | -0.372575 | -2.093955 |
| 1 | 6 | 0 | 5.363853 | -0.540823 | -0.583391 |
| 2 | 6 | 0 | 4.71984 | -2.832902 | 0.553898 |
| 3 | 6 | 0 | 6.248602 | -4.941972 | 0.20499 |
| 4 | 6 | 0 | 8.410203 | -4.759929 | -1.288922 |
| 5 | 6 | 0 | 9.059665 | -2.479472 | -2.470435 |
| 6 | 6 | 0 | 11.443034 | -2.253534 | -4.020143 |
| 7 | 6 | 0 | 13.794133 | -1.696694 | -2.408475 |
| 8 | 8 | 0 | 14.338027 | -3.635565 | -0.621467 |
| 9 | 6 | 0 | 0.789558 | -1.144429 | 1.671032 |
| 10 | 6 | 0 | 2.037909 | 1.488792 | 1.661483 |
| 11 | 6 | 0 | 0.041355 | 3.525879 | 1.053743 |
| 12 | 6 | 0 | -1.693355 | 2.823266 | -1.169776 |

| | | | | | |
|---|---|---|---|---|---|
| 13 | 6 | 0 | -2.425938 | 0.044558 | -1.231 |
| 14 | 8 | 0 | -0.373234 | -1.596807 | -0.696075 |
| 15 | 6 | 0 | 1.425167 | 5.912637 | 0.140705 |
| 16 | 6 | 0 | 0.907995 | 6.266014 | -2.661375 |
| 17 | 6 | 0 | -0.312001 | 3.782731 | -3.568689 |
| 18 | 8 | 0 | 2.721584 | 7.241249 | 1.488704 |
| 19 | 6 | 0 | -2.044053 | 4.102418 | -5.853812 |
| 20 | 8 | 0 | -4.376662 | -0.318343 | 0.582041 |
| 21 | 8 | 0 | 2.588919 | -3.045713 | 2.060227 |
| 22 | 8 | 0 | 3.897252 | 1.585429 | -0.284071 |
| 23 | 6 | 0 | 3.158551 | 1.984819 | 4.289287 |
| 24 | 8 | 0 | 5.654216 | 2.273263 | 4.257273 |
| 25 | 8 | 0 | 1.839293 | 2.048146 | 6.168998 |
| 26 | 6 | 0 | 6.827497 | 2.698147 | 6.685399 |
| 27 | 1 | 0 | -3.458562 | 3.882758 | -0.995408 |
| 28 | 1 | 0 | -0.995264 | 3.914205 | 2.795066 |
| 29 | 6 | 0 | -5.66796 | -2.688708 | 0.408222 |
| 30 | 6 | 0 | -7.50247 | -2.68864 | 2.642087 |
| 31 | 6 | 0 | -9.677444 | -0.821371 | 2.151574 |
| 32 | 6 | 0 | -10.988249 | -1.483263 | -0.366747 |
| 33 | 6 | 0 | -9.124947 | -1.741917 | -2.597419 |
| 34 | 8 | 0 | -6.822741 | -3.075027 | -1.935837 |
| 35 | 8 | 0 | -8.418462 | -5.221563 | 2.865583 |
| 36 | 8 | 0 | -12.451211 | -3.722413 | -0.069115 |
| 37 | 8 | 0 | -8.792338 | 1.73604 | 2.174183 |
| 38 | 6 | 0 | -8.657691 | 0.675728 | -4.122816 |
| 39 | 8 | 0 | -7.670105 | 2.791753 | -2.815308 |
| 40 | 1 | 0 | -0.566105 | -1.292136 | 3.21645 |
| 41 | 1 | 0 | 7.952669 | 1.435697 | -2.957939 |
| 42 | 1 | 0 | 5.714573 | -6.706237 | 1.104458 |
| 43 | 1 | 0 | 9.60013 | -6.409951 | -1.54604 |
| 44 | 1 | 0 | 11.772136 | -4.003776 | -5.085142 |
| 45 | 1 | 0 | 11.248395 | -0.732909 | -5.414957 |
| 46 | 1 | 0 | 15.420058 | -1.36027 | -3.6595 |
| 47 | 1 | 0 | 13.48721 | 0.019872 | -1.295235 |
| 48 | 1 | 0 | 14.745189 | -5.151293 | -1.570831 |
| 49 | 1 | 0 | -3.093147 | -0.542344 | -3.088865 |
| 50 | 1 | 0 | 2.630899 | 6.801861 | -3.669107 |
| 51 | 1 | 0 | -0.433526 | 7.843301 | -2.852938 |
| 52 | 1 | 0 | 1.196777 | 2.444591 | -4.032122 |
| 53 | 1 | 0 | -2.842645 | 2.289936 | -6.455932 |
| 54 | 1 | 0 | -3.623008 | 5.365445 | -5.411032 |

| | | | | | |
|---|---|---|---|---|---|
| 55 | 1 | 0 | -1.001086 | 4.891174 | -7.458787 |
| 56 | 1 | 0 | 8.840407 | 2.859505 | 6.29436 |
| 57 | 1 | 0 | 6.100125 | 4.438058 | 7.522261 |
| 58 | 1 | 0 | 6.448381 | 1.106601 | 7.942173 |
| 59 | 1 | 0 | -4.305284 | -4.229572 | 0.586667 |
| 60 | 1 | 0 | -6.454326 | -2.134202 | 4.339467 |
| 61 | 1 | 0 | -11.089707 | -1.003902 | 3.65282 |
| 62 | 1 | 0 | -12.33277 | 0.023826 | -0.81796 |
| 63 | 1 | 0 | -10.043492 | -3.034997 | -3.925224 |
| 64 | 1 | 0 | -9.206183 | -5.394596 | 4.514709 |
| 65 | 1 | 0 | -11.308157 | -4.964828 | 0.681004 |
| 66 | 1 | 0 | -6.964098 | 1.682746 | 1.905154 |
| 67 | 1 | 0 | -10.476502 | 1.145949 | -5.024697 |
| 68 | 1 | 0 | -7.315653 | 0.2372 | -5.63405 |
| 69 | 1 | 0 | -8.57676 | 2.942103 | -1.212694 |

| Compound**1**-DP4+000009_en_ | | Standard Orientation (A.U.) | | | |
|---|---|---|---|---|---|
| Center number | Atomic number | Atomic Type | X | Y | Z |
| 0 | 6 | 0 | -8.173725 | -0.600349 | 0.623426 |
| 1 | 6 | 0 | -5.849043 | 0.078312 | -0.3989 |
| 2 | 6 | 0 | -5.133539 | -0.818847 | -2.776032 |
| 3 | 6 | 0 | -6.771885 | -2.352118 | -4.143192 |
| 4 | 6 | 0 | -9.11136 | -3.006167 | -3.12363 |
| 5 | 6 | 0 | -9.836741 | -2.151506 | -0.724209 |
| 6 | 6 | 0 | -12.312482 | -2.957979 | 0.438974 |
| 7 | 6 | 0 | -12.108802 | -5.453903 | 1.909396 |
| 8 | 8 | 0 | -11.354518 | -7.530816 | 0.371976 |
| 9 | 6 | 0 | -1.033898 | 0.679307 | -2.077436 |
| 10 | 6 | 0 | -2.188324 | 2.678609 | -0.298609 |
| 11 | 6 | 0 | -0.229239 | 3.462806 | 1.714675 |
| 12 | 6 | 0 | 1.164472 | 1.205532 | 2.910093 |
| 13 | 6 | 0 | 1.801463 | -0.885862 | 1.034304 |
| 14 | 8 | 0 | -0.232276 | -1.428331 | -0.640906 |
| 15 | 6 | 0 | -1.646671 | 4.674032 | 3.943382 |
| 16 | 6 | 0 | -1.542223 | 2.890746 | 6.192205 |
| 17 | 6 | 0 | -0.491023 | 0.372325 | 5.177774 |
| 18 | 8 | 0 | -2.670729 | 6.724526 | 3.853622 |
| 19 | 6 | 0 | 0.951579 | -1.160961 | 7.15113 |
| 20 | 8 | 0 | 3.962574 | -0.102068 | -0.330558 |
| 21 | 8 | 0 | -2.826962 | -0.169644 | -3.830719 |

| | | | | | |
|---|---|---|---|---|---|
| 22 | 8 | 0 | -4.279153 | 1.600269 | 1.007878 |
| 23 | 6 | 0 | -3.110582 | 4.96011 | -1.838335 |
| 24 | 8 | 0 | -1.162639 | 6.083603 | -2.999477 |
| 25 | 8 | 0 | -5.296868 | 5.604213 | -2.018908 |
| 26 | 6 | 0 | -1.784633 | 8.235304 | -4.560589 |
| 27 | 1 | 0 | 2.965183 | 1.903202 | 3.656654 |
| 28 | 1 | 0 | 1.055559 | 4.826629 | 0.853663 |
| 29 | 6 | 0 | 5.141098 | -2.072574 | -1.724025 |
| 30 | 6 | 0 | 6.966133 | -0.889804 | -3.642302 |
| 31 | 6 | 0 | 9.417437 | 0.07194 | -2.416591 |
| 32 | 6 | 0 | 10.533165 | -1.978241 | -0.677605 |
| 33 | 6 | 0 | 8.577097 | -3.076034 | 1.153115 |
| 34 | 8 | 0 | 6.313439 | -3.859218 | -0.127368 |
| 35 | 8 | 0 | 7.534686 | -2.702017 | -5.541046 |
| 36 | 8 | 0 | 11.473366 | -3.885313 | -2.368012 |
| 37 | 8 | 0 | 8.886713 | 2.345776 | -1.091666 |
| 38 | 6 | 0 | 8.03374 | -1.353106 | 3.432483 |
| 39 | 8 | 0 | 10.238963 | -1.037123 | 4.944733 |
| 40 | 1 | 0 | 0.521699 | 1.479323 | -3.166472 |
| 41 | 1 | 0 | -8.669109 | 0.120827 | 2.480301 |
| 42 | 1 | 0 | -6.185368 | -3.020351 | -5.991543 |
| 43 | 1 | 0 | -10.379827 | -4.20653 | -4.199021 |
| 44 | 1 | 0 | -13.001635 | -1.501968 | 1.743539 |
| 45 | 1 | 0 | -13.756815 | -3.173238 | -1.035031 |
| 46 | 1 | 0 | -10.655948 | -5.285333 | 3.372734 |
| 47 | 1 | 0 | -13.919082 | -5.844571 | 2.854344 |
| 48 | 1 | 0 | -12.674131 | -7.79317 | -0.874648 |
| 49 | 1 | 0 | 2.169094 | -2.683923 | 1.979734 |
| 50 | 1 | 0 | -3.392837 | 2.746634 | 7.101082 |
| 51 | 1 | 0 | -0.23834 | 3.718545 | 7.583813 |
| 52 | 1 | 0 | -2.066485 | -0.753298 | 4.449364 |
| 53 | 1 | 0 | 1.69443 | -2.921605 | 6.353244 |
| 54 | 1 | 0 | 2.549819 | -0.075141 | 7.902113 |
| 55 | 1 | 0 | -0.278425 | -1.667258 | 8.736981 |
| 56 | 1 | 0 | 0.005121 | 8.873084 | -5.348738 |
| 57 | 1 | 0 | -3.085724 | 7.66828 | -6.05788 |
| 58 | 1 | 0 | -2.646522 | 9.713818 | -3.408393 |
| 59 | 1 | 0 | 3.710817 | -3.155013 | -2.748359 |
| 60 | 1 | 0 | 6.002509 | 0.691727 | -4.558734 |
| 61 | 1 | 0 | 10.774784 | 0.407693 | -3.950208 |
| 62 | 1 | 0 | 12.088259 | -1.186406 | 0.443252 |
| 63 | 1 | 0 | 9.341581 | -4.852276 | 1.907316 |

| | | | | | |
|---|---|---|---|---|---|
| 64 | 1 | 0 | 8.791031 | -3.833232 | -4.791535 |
| 65 | 1 | 0 | 11.957159 | -5.355837 | -1.382843 |
| 66 | 1 | 0 | 10.478547 | 3.006509 | -0.462482 |
| 67 | 1 | 0 | 6.470158 | -2.178792 | 4.519921 |
| 68 | 1 | 0 | 7.485587 | 0.526107 | 2.804223 |
| 69 | 1 | 0 | 10.624033 | -2.654761 | 5.720186 |

**Table S4.** Cartesian coordinates for the low-energy reoptimized random research conformers of compound **2** (Unit A and the phenylethanoid moiety in unit B) (obtained by DP4+ calculation)

| Compound**2**-DP4+000001_en_ | | Standard Orientation (A.U.) | | | |
|---|---|---|---|---|---|
| Center number | Atomic number | Atomic Type | X | Y | Z |
| 0 | 8 | 0 | 10.053568 | -2.474759 | 3.103656 |
| 1 | 6 | 0 | 6.611362 | -4.332768 | -1.218391 |
| 2 | 8 | 0 | 13.286806 | -2.541704 | -3.069626 |
| 3 | 6 | 0 | 10.841935 | 1.168544 | 0.552889 |
| 4 | 6 | 0 | 11.591673 | -1.584859 | 1.090107 |
| 5 | 6 | 0 | 11.332176 | -3.182595 | -1.335813 |
| 6 | 6 | 0 | 8.718127 | -2.872112 | -2.559976 |
| 7 | 8 | 0 | 8.122326 | -0.237922 | -2.944358 |
| 8 | 6 | 0 | 8.34772 | 1.420809 | -0.894177 |
| 9 | 1 | 0 | 2.294509 | 2.48763 | 3.478266 |
| 10 | 8 | 0 | -12.561047 | -4.315925 | -4.972688 |
| 11 | 6 | 0 | -14.457524 | -3.434536 | -3.277768 |
| 12 | 6 | 0 | -13.867846 | -4.026354 | -0.497102 |
| 13 | 6 | 0 | -11.443633 | -2.823827 | 0.404384 |
| 14 | 6 | 0 | -9.13114 | -4.078201 | 0.170109 |
| 15 | 6 | 0 | -6.870029 | -2.927362 | 0.911674 |
| 16 | 6 | 0 | -6.928915 | -0.495057 | 1.898016 |
| 17 | 6 | 0 | -9.23165 | 0.804713 | 2.126724 |
| 18 | 6 | 0 | -11.46642 | -0.36281 | 1.386554 |
| 19 | 8 | 0 | -4.879713 | 0.879446 | 2.749648 |
| 20 | 8 | 0 | -9.269449 | 3.207929 | 3.069773 |
| 21 | 6 | 0 | -2.455567 | 0.099961 | 2.152364 |
| 22 | 6 | 0 | -1.09421 | -1.599725 | 4.01642 |
| 23 | 6 | 0 | 1.695557 | -1.550309 | 3.122032 |
| 24 | 6 | 0 | 1.964574 | 1.111454 | 1.956199 |
| 25 | 6 | 0 | -0.622791 | 1.721646 | 0.83774 |
| 26 | 6 | 0 | 3.950215 | 1.571458 | -0.036625 |

| | | | | | |
|---|---|---|---|---|---|
| 27 | 8 | 0 | 3.802331 | 4.248312 | -0.78514 |
| 28 | 6 | 0 | 1.510592 | 5.261003 | -1.082507 |
| 29 | 6 | 0 | -0.741039 | 4.195751 | -0.446049 |
| 30 | 8 | 0 | -1.849609 | -0.47068 | -0.398163 |
| 31 | 6 | 0 | 3.573166 | -2.110565 | 5.240698 |
| 32 | 6 | 0 | -3.006333 | 5.595572 | -1.286248 |
| 33 | 8 | 0 | -5.1106 | 4.168647 | -1.308106 |
| 34 | 8 | 0 | -2.983498 | 7.797173 | -1.988939 |
| 35 | 8 | 0 | 6.357721 | 1.177473 | 0.915465 |
| 36 | 6 | 0 | -7.349188 | 5.377359 | -2.271496 |
| 37 | 8 | 0 | 12.686814 | 2.310666 | -1.074162 |
| 38 | 8 | 0 | 4.212067 | -4.014156 | -2.393327 |
| 39 | 1 | 0 | 10.624486 | -4.160659 | 3.54753 |
| 40 | 1 | 0 | 6.392216 | -3.688706 | 0.719264 |
| 41 | 1 | 0 | 7.1599 | -6.338648 | -1.172981 |
| 42 | 1 | 0 | 13.297152 | -0.696508 | -3.153962 |
| 43 | 1 | 0 | 10.671081 | 2.208226 | 2.333468 |
| 44 | 1 | 0 | 13.595302 | -1.605651 | 1.640791 |
| 45 | 1 | 0 | 11.607648 | -5.180353 | -0.862363 |
| 46 | 1 | 0 | 8.87761 | -3.595858 | -4.493259 |
| 47 | 1 | 0 | 8.265371 | 3.303179 | -1.737034 |
| 48 | 1 | 0 | -12.493921 | -6.141823 | -4.811307 |
| 49 | 1 | 0 | -14.543722 | -1.38581 | -3.553662 |
| 50 | 1 | 0 | -16.319403 | -4.219878 | -3.769157 |
| 51 | 1 | 0 | -15.467117 | -3.370463 | 0.647819 |
| 52 | 1 | 0 | -13.772566 | -6.086313 | -0.265386 |
| 53 | 1 | 0 | -9.078898 | -5.976684 | -0.605878 |
| 54 | 1 | 0 | -5.089127 | -3.914524 | 0.69661 |
| 55 | 1 | 0 | -13.227089 | 0.675507 | 1.57952 |
| 56 | 1 | 0 | -7.51333 | 3.715566 | 3.296444 |
| 57 | 1 | 0 | -1.884076 | -3.510386 | 4.059461 |
| 58 | 1 | 0 | -1.283413 | -0.785021 | 5.912615 |
| 59 | 1 | 0 | 1.912367 | -2.936625 | 1.604918 |
| 60 | 1 | 0 | 3.624272 | 0.457204 | -1.745805 |
| 61 | 1 | 0 | 1.589311 | 7.115645 | -1.951737 |
| 62 | 1 | 0 | 5.523543 | -2.017532 | 4.568498 |
| 63 | 1 | 0 | 3.238046 | -3.999768 | 6.021563 |
| 64 | 1 | 0 | 3.359542 | -0.74222 | 6.783913 |
| 65 | 1 | 0 | -7.915687 | 6.9371 | -1.044423 |
| 66 | 1 | 0 | -7.01205 | 6.087588 | -4.179528 |
| 67 | 1 | 0 | -8.792669 | 3.912034 | -2.278465 |
| 68 | 1 | 0 | 14.248635 | 2.482764 | -0.125215 |

| | | | | | |
|---|---|---|---|---|---|
| 69 | 1 | 0 | 4.345514 | -4.588157 | -4.131336 |

| Compound**2**-DP4+000002_en_ | | Standard Orientation (A.U.) | | | |
|---|---|---|---|---|---|
| Center number | Atomic number | Atomic Type | X | Y | Z |
| 0 | 8 | 0 | 9.757489 | -3.172871 | 3.0456 |
| 1 | 6 | 0 | 6.53828 | -4.247275 | -1.720443 |
| 2 | 8 | 0 | 13.2649 | -2.435715 | -2.948668 |
| 3 | 6 | 0 | 10.907301 | 0.749339 | 1.05686 |
| 4 | 6 | 0 | 11.464162 | -2.091521 | 1.276225 |
| 5 | 6 | 0 | 11.264236 | -3.348016 | -1.348533 |
| 6 | 6 | 0 | 8.787964 | -2.705406 | -2.6948 |
| 7 | 8 | 0 | 8.32143 | -0.026263 | -2.725053 |
| 8 | 6 | 0 | 8.486422 | 1.311871 | -0.438371 |
| 9 | 1 | 0 | 2.188641 | 1.97706 | 3.703502 |
| 10 | 8 | 0 | -13.611374 | -7.042624 | -0.087468 |
| 11 | 6 | 0 | -15.140072 | -4.903342 | -0.661004 |
| 12 | 6 | 0 | -13.837249 | -3.06016 | -2.491375 |
| 13 | 6 | 0 | -11.424186 | -2.012492 | -1.383879 |
| 14 | 6 | 0 | -9.134622 | -3.30803 | -1.662466 |
| 15 | 6 | 0 | -6.914508 | -2.422933 | -0.526882 |
| 16 | 6 | 0 | -6.9934 | -0.228797 | 0.908624 |
| 17 | 6 | 0 | -9.272442 | 1.107206 | 1.196474 |
| 18 | 6 | 0 | -11.465585 | 0.207653 | 0.06485 |
| 19 | 8 | 0 | -4.989939 | 0.872719 | 2.161764 |
| 20 | 8 | 0 | -9.320526 | 3.28742 | 2.572237 |
| 21 | 6 | 0 | -2.545695 | 0.10111 | 1.629101 |
| 22 | 6 | 0 | -1.41096 | -1.965147 | 3.258631 |
| 23 | 6 | 0 | 1.444286 | -1.902087 | 2.605379 |
| 24 | 6 | 0 | 1.91222 | 0.902796 | 1.946374 |
| 25 | 6 | 0 | -0.553036 | 1.839611 | 0.778168 |
| 26 | 6 | 0 | 4.056794 | 1.582527 | 0.194209 |
| 27 | 8 | 0 | 4.084282 | 4.355494 | -0.076114 |
| 28 | 6 | 0 | 1.870094 | 5.534632 | -0.328812 |
| 29 | 6 | 0 | -0.469085 | 4.505523 | -0.040916 |
| 30 | 8 | 0 | -1.747328 | -0.034949 | -0.927398 |
| 31 | 6 | 0 | 3.114435 | -2.907378 | 4.733804 |
| 32 | 6 | 0 | -2.596913 | 6.169604 | -0.746107 |
| 33 | 8 | 0 | -4.768686 | 4.90853 | -1.149675 |
| 34 | 8 | 0 | -2.411549 | 8.454918 | -1.036203 |
| 35 | 8 | 0 | 6.372147 | 0.891529 | 1.196711 |

| | | | | | |
|---|---|---|---|---|---|
| 36 | 6 | 0 | -6.862843 | 6.417092 | -2.011504 |
| 37 | 8 | 0 | 12.969899 | 1.995976 | -0.129471 |
| 38 | 8 | 0 | 4.246432 | -3.625527 | -2.977778 |
| 39 | 1 | 0 | 10.260716 | -4.914269 | 3.328923 |
| 40 | 1 | 0 | 6.219612 | -3.886632 | 0.275866 |
| 41 | 1 | 0 | 6.996053 | -6.262738 | -1.949394 |
| 42 | 1 | 0 | 14.850046 | -3.101989 | -2.303954 |
| 43 | 1 | 0 | 10.699677 | 1.550637 | 2.94898 |
| 44 | 1 | 0 | 13.421969 | -2.276287 | 1.945628 |
| 45 | 1 | 0 | 11.404219 | -5.409292 | -1.152461 |
| 46 | 1 | 0 | 9.059787 | -3.155739 | -4.697653 |
| 47 | 1 | 0 | 8.513957 | 3.296557 | -1.00274 |
| 48 | 1 | 0 | -12.039462 | -6.393389 | 0.60893 |
| 49 | 1 | 0 | -16.86597 | -5.649548 | -1.522214 |
| 50 | 1 | 0 | -15.679007 | -3.888539 | 1.073635 |
| 51 | 1 | 0 | -13.435577 | -4.064143 | -4.257855 |
| 52 | 1 | 0 | -15.158852 | -1.52565 | -2.929848 |
| 53 | 1 | 0 | -9.062393 | -5.00966 | -2.807436 |
| 54 | 1 | 0 | -5.149033 | -3.423651 | -0.795588 |
| 55 | 1 | 0 | -13.205659 | 1.268048 | 0.311776 |
| 56 | 1 | 0 | -7.576581 | 3.6693 | 3.027192 |
| 57 | 1 | 0 | -2.27116 | -3.810996 | 2.898456 |
| 58 | 1 | 0 | -1.73245 | -1.485463 | 5.248909 |
| 59 | 1 | 0 | 1.739015 | -3.019384 | 0.892457 |
| 60 | 1 | 0 | 3.794803 | 0.806805 | -1.70322 |
| 61 | 1 | 0 | 2.095039 | 7.507315 | -0.838454 |
| 62 | 1 | 0 | 2.809783 | -1.821231 | 6.473588 |
| 63 | 1 | 0 | 5.117564 | -2.784538 | 4.246175 |
| 64 | 1 | 0 | 2.657985 | -4.887181 | 5.137627 |
| 65 | 1 | 0 | -8.382442 | 5.078219 | -2.369794 |
| 66 | 1 | 0 | -7.425945 | 7.771682 | -0.559646 |
| 67 | 1 | 0 | -6.351902 | 7.428479 | -3.736225 |
| 68 | 1 | 0 | 13.354111 | 0.996322 | -1.636856 |
| 69 | 1 | 0 | 4.462467 | -3.967535 | -4.767768 |

| Compound**2**-DP4+000003_en_ | | Standard Orientation (A.U.) | | | |
|---|---|---|---|---|---|
| Center number | Atomic number | Atomic Type | X | Y | Z |
| 0 | 8 | 0 | 14.79347 | -0.102612 | 0.153812 |
| 1 | 6 | 0 | 6.932717 | -4.824471 | -0.017773 |
| 2 | 8 | 0 | 13.179519 | -5.037318 | -1.295455 |

| | | | | | |
|---|---|---|---|---|---|
| 3 | 6 | 0 | 10.580596 | 1.411593 | 0.020666 |
| 4 | 6 | 0 | 12.435177 | -0.497709 | -1.058254 |
| 5 | 6 | 0 | 11.423234 | -3.143104 | -0.534401 |
| 6 | 6 | 0 | 8.79903 | -3.484623 | -1.753088 |
| 7 | 8 | 0 | 7.72384 | -1.135984 | -2.665718 |
| 8 | 6 | 0 | 7.987094 | 1.091005 | -1.23151 |
| 9 | 1 | 0 | 2.122588 | 2.814577 | 3.397323 |
| 10 | 8 | 0 | -12.532431 | -4.491123 | -4.984474 |
| 11 | 6 | 0 | -14.475026 | -3.682817 | -3.305757 |
| 12 | 6 | 0 | -13.887229 | -4.25482 | -0.520593 |
| 13 | 6 | 0 | -11.50895 | -2.974471 | 0.395996 |
| 14 | 6 | 0 | -9.162594 | -4.17515 | 0.227714 |
| 15 | 6 | 0 | -6.943239 | -2.951633 | 0.97939 |
| 16 | 6 | 0 | -7.079479 | -0.499508 | 1.90621 |
| 17 | 6 | 0 | -9.417325 | 0.74578 | 2.072478 |
| 18 | 6 | 0 | -11.609648 | -0.494453 | 1.323938 |
| 19 | 8 | 0 | -5.078269 | 0.948248 | 2.754769 |
| 20 | 8 | 0 | -9.532304 | 3.166826 | 2.961343 |
| 21 | 6 | 0 | -2.626322 | 0.223199 | 2.217801 |
| 22 | 6 | 0 | -1.238644 | -1.351145 | 4.170102 |
| 23 | 6 | 0 | 1.564818 | -1.251335 | 3.321254 |
| 24 | 6 | 0 | 1.772334 | 1.334494 | 1.983417 |
| 25 | 6 | 0 | -0.828154 | 1.839197 | 0.847839 |
| 26 | 6 | 0 | 3.693387 | 1.669749 | -0.093808 |
| 27 | 8 | 0 | 3.556083 | 4.289786 | -0.975601 |
| 28 | 6 | 0 | 1.250152 | 5.282404 | -1.294226 |
| 29 | 6 | 0 | -0.983058 | 4.241218 | -0.566321 |
| 30 | 8 | 0 | -1.971616 | -0.443468 | -0.298439 |
| 31 | 6 | 0 | 3.416022 | -1.569837 | 5.513312 |
| 32 | 6 | 0 | -3.271721 | 5.58089 | -1.449002 |
| 33 | 8 | 0 | -5.357392 | 4.130949 | -1.39164 |
| 34 | 8 | 0 | -3.273317 | 7.748285 | -2.247682 |
| 35 | 8 | 0 | 6.139815 | 1.261594 | 0.739142 |
| 36 | 6 | 0 | -7.616335 | 5.267848 | -2.397794 |
| 37 | 8 | 0 | 11.377177 | 3.937163 | -0.40182 |
| 38 | 8 | 0 | 4.559437 | -5.197788 | -1.221419 |
| 39 | 1 | 0 | 15.813487 | -1.587882 | -0.213571 |
| 40 | 1 | 0 | 6.718209 | -3.746044 | 1.740132 |
| 41 | 1 | 0 | 7.680798 | -6.69214 | 0.462684 |
| 42 | 1 | 0 | 13.337305 | -4.966452 | -3.125834 |
| 43 | 1 | 0 | 10.385704 | 1.039754 | 2.056073 |
| 44 | 1 | 0 | 12.606382 | -0.204994 | -3.11253 |

| | | | | | |
|---|---|---|---|---|---|
| 45 | 1 | 0 | 11.291429 | -3.368418 | 1.520528 |
| 46 | 1 | 0 | 9.009396 | -4.598318 | -3.483022 |
| 47 | 1 | 0 | 7.687967 | 2.623866 | -2.578987 |
| 48 | 1 | 0 | -12.394935 | -6.312762 | -4.820096 |
| 49 | 1 | 0 | -14.63488 | -1.638427 | -3.581182 |
| 50 | 1 | 0 | -16.30232 | -4.536059 | -3.813528 |
| 51 | 1 | 0 | -15.515113 | -3.649981 | 0.61219 |
| 52 | 1 | 0 | -13.726787 | -6.310249 | -0.286097 |
| 53 | 1 | 0 | -9.049808 | -6.088977 | -0.50321 |
| 54 | 1 | 0 | -5.135244 | -3.898709 | 0.816794 |
| 55 | 1 | 0 | -13.39864 | 0.502716 | 1.466851 |
| 56 | 1 | 0 | -7.794744 | 3.72698 | 3.205268 |
| 57 | 1 | 0 | -1.970247 | -3.281984 | 4.27875 |
| 58 | 1 | 0 | -1.48606 | -0.465993 | 6.026966 |
| 59 | 1 | 0 | 1.882938 | -2.744428 | 1.925381 |
| 60 | 1 | 0 | 3.242301 | 0.491733 | -1.739054 |
| 61 | 1 | 0 | 1.312893 | 7.086159 | -2.263998 |
| 62 | 1 | 0 | 5.370133 | -1.355648 | 4.877952 |
| 63 | 1 | 0 | 3.212172 | -3.439485 | 6.378105 |
| 64 | 1 | 0 | 3.067029 | -0.146165 | 6.978349 |
| 65 | 1 | 0 | -8.202708 | 6.865745 | -1.230748 |
| 66 | 1 | 0 | -7.290996 | 5.907528 | -4.332462 |
| 67 | 1 | 0 | -9.037388 | 3.782136 | -2.344782 |
| 68 | 1 | 0 | 13.095063 | 4.038806 | 0.24221 |
| 69 | 1 | 0 | 4.190985 | -3.637158 | -2.116593 |

| Compound**2**-DP4+000004_en_ | | Standard Orientation (A.U.) | | | |
|---|---|---|---|---|---|
| Center number | Atomic number | Atomic Type | X | Y | Z |
| 0 | 8 | 0 | 10.142463 | -2.062959 | 3.06171 |
| 1 | 6 | 0 | 6.761967 | -4.26843 | -1.277615 |
| 2 | 8 | 0 | 13.280646 | -2.241021 | -3.174997 |
| 3 | 6 | 0 | 10.863828 | 1.47341 | 0.304087 |
| 4 | 6 | 0 | 11.660604 | -1.226514 | 1.00946 |
| 5 | 6 | 0 | 11.425615 | -2.963427 | -1.318218 |
| 6 | 6 | 0 | 8.854319 | -2.762218 | -2.612798 |
| 7 | 8 | 0 | 8.112505 | -0.182731 | -3.067078 |
| 8 | 6 | 0 | 8.314971 | 1.564092 | -1.076972 |
| 9 | 1 | 0 | 2.324518 | 2.546291 | 3.453046 |
| 10 | 8 | 0 | -16.579491 | -4.52724 | -4.333248 |
| 11 | 6 | 0 | -14.36999 | -3.326874 | -3.371162 |

| | | | | | |
|---|---|---|---|---|---|
| 12 | 6 | 0 | -13.767401 | -4.11187 | -0.641779 |
| 13 | 6 | 0 | -11.365692 | -2.891147 | 0.298182 |
| 14 | 6 | 0 | -9.03311 | -4.100963 | 0.023068 |
| 15 | 6 | 0 | -6.788767 | -2.933388 | 0.789252 |
| 16 | 6 | 0 | -6.885701 | -0.529439 | 1.839289 |
| 17 | 6 | 0 | -9.208686 | 0.726883 | 2.108713 |
| 18 | 6 | 0 | -11.426486 | -0.455709 | 1.342084 |
| 19 | 8 | 0 | -4.857161 | 0.855436 | 2.721982 |
| 20 | 8 | 0 | -9.283248 | 3.102053 | 3.114694 |
| 21 | 6 | 0 | -2.422967 | 0.129042 | 2.104639 |
| 22 | 6 | 0 | -1.0328 | -1.59402 | 3.925526 |
| 23 | 6 | 0 | 1.753568 | -1.485903 | 3.025828 |
| 24 | 6 | 0 | 1.983332 | 1.199642 | 1.908446 |
| 25 | 6 | 0 | -0.618099 | 1.809286 | 0.82555 |
| 26 | 6 | 0 | 3.937267 | 1.723158 | -0.100658 |
| 27 | 8 | 0 | 3.772657 | 4.418565 | -0.757637 |
| 28 | 6 | 0 | 1.467448 | 5.418478 | -1.016659 |
| 29 | 6 | 0 | -0.768571 | 4.312835 | -0.397047 |
| 30 | 8 | 0 | -1.813273 | -0.370651 | -0.461314 |
| 31 | 6 | 0 | 3.650446 | -2.058709 | 5.123427 |
| 32 | 6 | 0 | -3.05471 | 5.709921 | -1.18771 |
| 33 | 8 | 0 | -5.14207 | 4.260034 | -1.235762 |
| 34 | 8 | 0 | -3.058376 | 7.928803 | -1.831513 |
| 35 | 8 | 0 | 6.358528 | 1.281329 | 0.773314 |
| 36 | 6 | 0 | -7.399948 | 5.469739 | -2.15374 |
| 37 | 8 | 0 | 12.736148 | 2.609583 | -1.248701 |
| 38 | 8 | 0 | 7.339974 | -6.896025 | -1.220045 |
| 39 | 1 | 0 | 10.706633 | -3.743549 | 3.534765 |
| 40 | 1 | 0 | 4.970371 | -3.899605 | -2.259124 |
| 41 | 1 | 0 | 6.578677 | -3.673913 | 0.681055 |
| 42 | 1 | 0 | 14.938911 | -2.675244 | -2.516246 |
| 43 | 1 | 0 | 10.701035 | 2.610169 | 2.020898 |
| 44 | 1 | 0 | 13.660086 | -1.145437 | 1.570602 |
| 45 | 1 | 0 | 11.699886 | -4.942299 | -0.76766 |
| 46 | 1 | 0 | 9.083968 | -3.540415 | -4.517135 |
| 47 | 1 | 0 | 8.122861 | 3.407429 | -1.982899 |
| 48 | 1 | 0 | -17.99668 | -3.940924 | -3.327082 |
| 49 | 1 | 0 | -12.821329 | -3.900725 | -4.617213 |
| 50 | 1 | 0 | -14.52603 | -1.256031 | -3.484338 |
| 51 | 1 | 0 | -15.363647 | -3.578007 | 0.571236 |
| 52 | 1 | 0 | -13.607194 | -6.175255 | -0.5771 |
| 53 | 1 | 0 | -8.953812 | -5.981524 | -0.796189 |

| | | | | | |
|---|---|---|---|---|---|
| 54 | 1 | 0 | -4.993458 | -3.887558 | 0.547754 |
| 55 | 1 | 0 | -13.203681 | 0.546462 | 1.568902 |
| 56 | 1 | 0 | -7.536039 | 3.63228 | 3.357796 |
| 57 | 1 | 0 | -1.795374 | -3.515792 | 3.928308 |
| 58 | 1 | 0 | -1.225355 | -0.824505 | 5.839694 |
| 59 | 1 | 0 | 1.981382 | -2.852262 | 1.489694 |
| 60 | 1 | 0 | 3.556047 | 0.688437 | -1.854468 |
| 61 | 1 | 0 | 1.525219 | 7.296214 | -1.835708 |
| 62 | 1 | 0 | 5.595538 | -1.903876 | 4.447251 |
| 63 | 1 | 0 | 3.358442 | -3.972529 | 5.859054 |
| 64 | 1 | 0 | 3.411494 | -0.731594 | 6.697987 |
| 65 | 1 | 0 | -7.081701 | 6.233307 | -4.044263 |
| 66 | 1 | 0 | -8.827969 | 3.989854 | -2.190688 |
| 67 | 1 | 0 | -7.974349 | 6.990607 | -0.88247 |
| 68 | 1 | 0 | 13.144772 | 1.34995 | -2.540351 |
| 69 | 1 | 0 | 7.289768 | -7.518651 | -2.945524 |

| Compound**2**-DP4+000005_en_ | | Standard Orientation (A.U.) | | | |
|---|---|---|---|---|---|
| Center number | Atomic number | Atomic Type | X | Y | Z |
| 0 | 8 | 0 | 9.918961 | -3.193864 | 3.00574 |
| 1 | 6 | 0 | 6.766968 | -4.178823 | -1.78032 |
| 2 | 8 | 0 | 13.59413 | -2.12549 | -2.695666 |
| 3 | 6 | 0 | 10.971249 | 0.833025 | 1.179121 |
| 4 | 6 | 0 | 11.612341 | -1.996872 | 1.301504 |
| 5 | 6 | 0 | 11.477928 | -3.169528 | -1.35922 |
| 6 | 6 | 0 | 8.983214 | -2.556378 | -2.703232 |
| 7 | 8 | 0 | 8.459421 | 0.108432 | -2.67383 |
| 8 | 6 | 0 | 8.566257 | 1.385666 | -0.34712 |
| 9 | 1 | 0 | 2.203481 | 1.81738 | 3.714187 |
| 10 | 8 | 0 | -16.188728 | -3.571112 | 1.184781 |
| 11 | 6 | 0 | -15.256175 | -5.003537 | -0.896418 |
| 12 | 6 | 0 | -13.64689 | -3.438982 | -2.738784 |
| 13 | 6 | 0 | -11.27681 | -2.385655 | -1.551305 |
| 14 | 6 | 0 | -8.959961 | -3.624162 | -1.830874 |
| 15 | 6 | 0 | -6.767372 | -2.709571 | -0.670223 |
| 16 | 6 | 0 | -6.902472 | -0.534008 | 0.792081 |
| 17 | 6 | 0 | -9.209473 | 0.748058 | 1.078841 |
| 18 | 6 | 0 | -11.374099 | -0.180475 | -0.084384 |
| 19 | 8 | 0 | -4.928561 | 0.59419 | 2.075038 |
| 20 | 8 | 0 | -9.316116 | 2.910333 | 2.486649 |

| | | | | | |
|---|---|---|---|---|---|
| 21 | 6 | 0 | -2.466648 | -0.116212 | 1.550522 |
| 22 | 6 | 0 | -1.302448 | -2.192691 | 3.147016 |
| 23 | 6 | 0 | 1.557815 | -2.053131 | 2.528603 |
| 24 | 6 | 0 | 1.9701 | 0.774153 | 1.932232 |
| 25 | 6 | 0 | -0.50242 | 1.681013 | 0.756144 |
| 26 | 6 | 0 | 4.119161 | 1.53607 | 0.220185 |
| 27 | 8 | 0 | 4.089097 | 4.312924 | 0.003604 |
| 28 | 6 | 0 | 1.852213 | 5.449842 | -0.246796 |
| 29 | 6 | 0 | -0.466959 | 4.365596 | -0.004796 |
| 30 | 8 | 0 | -1.637852 | -0.180805 | -1.000935 |
| 31 | 6 | 0 | 3.226148 | -3.064491 | 4.655839 |
| 32 | 6 | 0 | -2.622734 | 5.999926 | -0.695238 |
| 33 | 8 | 0 | -4.761772 | 4.702052 | -1.150981 |
| 34 | 8 | 0 | -2.48385 | 8.294915 | -0.931155 |
| 35 | 8 | 0 | 6.436182 | 0.875811 | 1.240386 |
| 36 | 6 | 0 | -6.880546 | 6.182795 | -1.999926 |
| 37 | 8 | 0 | 13.020243 | 2.195924 | 0.104804 |
| 38 | 8 | 0 | 4.471608 | -3.604991 | -3.05448 |
| 39 | 1 | 0 | 10.484046 | -4.922825 | 3.244552 |
| 40 | 1 | 0 | 6.417115 | -3.856967 | 0.216852 |
| 41 | 1 | 0 | 7.285643 | -6.176421 | -2.032586 |
| 42 | 1 | 0 | 13.453455 | -2.572745 | -4.469782 |
| 43 | 1 | 0 | 10.688954 | 1.543474 | 3.097832 |
| 44 | 1 | 0 | 13.569698 | -2.15182 | 1.969696 |
| 45 | 1 | 0 | 11.681198 | -5.232015 | -1.219508 |
| 46 | 1 | 0 | 9.239455 | -2.956597 | -4.723697 |
| 47 | 1 | 0 | 8.56175 | 3.385136 | -0.85655 |
| 48 | 1 | 0 | -17.313491 | -2.292854 | 0.502476 |
| 49 | 1 | 0 | -14.104217 | -6.508823 | -0.067594 |
| 50 | 1 | 0 | -16.816983 | -5.89882 | -1.938398 |
| 51 | 1 | 0 | -13.154321 | -4.654074 | -4.344176 |
| 52 | 1 | 0 | -14.821521 | -1.902427 | -3.492981 |
| 53 | 1 | 0 | -8.84756 | -5.324368 | -2.976167 |
| 54 | 1 | 0 | -4.978852 | -3.669472 | -0.937816 |
| 55 | 1 | 0 | -13.137697 | 0.83452 | 0.167428 |
| 56 | 1 | 0 | -7.583824 | 3.321082 | 2.959239 |
| 57 | 1 | 0 | -2.117476 | -4.048666 | 2.737612 |
| 58 | 1 | 0 | -1.65616 | -1.764207 | 5.143513 |
| 59 | 1 | 0 | 1.897143 | -3.128455 | 0.797175 |
| 60 | 1 | 0 | 3.898397 | 0.791557 | -1.695047 |
| 61 | 1 | 0 | 2.040239 | 7.437204 | -0.712374 |
| 62 | 1 | 0 | 5.231709 | -2.88752 | 4.195145 |

| | | | | | |
|---|---|---|---|---|---|
| 63 | 1 | 0 | 2.80977 | -5.061766 | 5.014383 |
| 64 | 1 | 0 | 2.877516 | -2.021051 | 6.413402 |
| 65 | 1 | 0 | -6.376735 | 7.241646 | -3.698168 |
| 66 | 1 | 0 | -8.367292 | 4.818985 | -2.399381 |
| 67 | 1 | 0 | -7.486008 | 7.493605 | -0.525134 |
| 68 | 1 | 0 | 13.526346 | 1.239562 | -1.394327 |
| 69 | 1 | 0 | 4.703766 | -3.948553 | -4.842142 |

| Compound**2**-DP4+000006_en_ | | Standard Orientation (A.U.) | | | |
|---|---|---|---|---|---|
| Center number | Atomic number | Atomic Type | X | Y | Z |
| 0 | 8 | 0 | 9.895375 | -3.193197 | 3.021655 |
| 1 | 6 | 0 | 6.734243 | -4.201128 | -1.755427 |
| 2 | 8 | 0 | 13.569364 | -2.184763 | -2.691458 |
| 3 | 6 | 0 | 10.96363 | 0.81481 | 1.163463 |
| 4 | 6 | 0 | 11.592236 | -2.016918 | 1.30653 |
| 5 | 6 | 0 | 11.449641 | -3.208933 | -1.345249 |
| 6 | 6 | 0 | 8.95637 | -2.594492 | -2.691488 |
| 7 | 8 | 0 | 8.44323 | 0.072588 | -2.680274 |
| 8 | 6 | 0 | 8.558462 | 1.366026 | -0.362918 |
| 9 | 1 | 0 | 2.204415 | 1.84425 | 3.708701 |
| 10 | 8 | 0 | -15.968023 | -3.960462 | 1.26821 |
| 11 | 6 | 0 | -15.123346 | -5.147637 | -0.995765 |
| 12 | 6 | 0 | -13.663907 | -3.356547 | -2.754342 |
| 13 | 6 | 0 | -11.291582 | -2.309748 | -1.56592 |
| 14 | 6 | 0 | -8.973939 | -3.542369 | -1.866808 |
| 15 | 6 | 0 | -6.780275 | -2.639906 | -0.699926 |
| 16 | 6 | 0 | -6.913813 | -0.48052 | 0.787322 |
| 17 | 6 | 0 | -9.221034 | 0.795344 | 1.095268 |
| 18 | 6 | 0 | -11.388559 | -0.125102 | -0.070294 |
| 19 | 8 | 0 | -4.937221 | 0.637202 | 2.07497 |
| 20 | 8 | 0 | -9.326171 | 2.943422 | 2.524655 |
| 21 | 6 | 0 | -2.476904 | -0.079399 | 1.551711 |
| 22 | 6 | 0 | -1.32052 | -2.156673 | 3.152392 |
| 23 | 6 | 0 | 1.540556 | -2.02789 | 2.535982 |
| 24 | 6 | 0 | 1.963499 | 0.796151 | 1.930678 |
| 25 | 6 | 0 | -0.506286 | 1.71 | 0.754496 |
| 26 | 6 | 0 | 4.11328 | 1.542054 | 0.21232 |
| 27 | 8 | 0 | 4.09525 | 4.318119 | -0.015915 |
| 28 | 6 | 0 | 1.863141 | 5.46475 | -0.265121 |
| 29 | 6 | 0 | -0.46047 | 4.392298 | -0.013906 |

| | | | | | |
|---|---|---|---|---|---|
| 30 | 8 | 0 | -1.647505 | -0.151233 | -0.999249 |
| 31 | 6 | 0 | 3.20384 | -3.037225 | 4.668183 |
| 32 | 6 | 0 | -2.611278 | 6.034173 | -0.70154 |
| 33 | 8 | 0 | -4.758293 | 4.744229 | -1.140905 |
| 34 | 8 | 0 | -2.462899 | 8.327583 | -0.947101 |
| 35 | 8 | 0 | 6.428937 | 0.875758 | 1.231595 |
| 36 | 6 | 0 | -6.877619 | 6.23123 | -1.976646 |
| 37 | 8 | 0 | 13.016653 | 2.160437 | 0.075234 |
| 38 | 8 | 0 | 4.440208 | -3.626027 | -3.031455 |
| 39 | 1 | 0 | 10.455033 | -4.921665 | 3.276118 |
| 40 | 1 | 0 | 6.38782 | -3.864106 | 0.239818 |
| 41 | 1 | 0 | 7.244084 | -6.202627 | -1.994504 |
| 42 | 1 | 0 | 13.42599 | -2.646338 | -4.461668 |
| 43 | 1 | 0 | 10.687669 | 1.541187 | 3.077126 |
| 44 | 1 | 0 | 13.549635 | -2.1756 | 1.973687 |
| 45 | 1 | 0 | 11.64344 | -5.271291 | -1.190458 |
| 46 | 1 | 0 | 9.209242 | -3.010005 | -4.70933 |
| 47 | 1 | 0 | 8.560905 | 3.361794 | -0.886667 |
| 48 | 1 | 0 | -17.149667 | -2.64415 | 0.783284 |
| 49 | 1 | 0 | -13.885549 | -6.690326 | -0.388832 |
| 50 | 1 | 0 | -16.717595 | -5.984628 | -2.035981 |
| 51 | 1 | 0 | -13.193599 | -4.404296 | -4.480248 |
| 52 | 1 | 0 | -14.928039 | -1.810615 | -3.320107 |
| 53 | 1 | 0 | -8.862509 | -5.228188 | -3.033504 |
| 54 | 1 | 0 | -4.991181 | -3.59424 | -0.982739 |
| 55 | 1 | 0 | -13.152501 | 0.887128 | 0.196106 |
| 56 | 1 | 0 | -7.593594 | 3.34933 | 3.000313 |
| 57 | 1 | 0 | -2.142103 | -4.01028 | 2.744971 |
| 58 | 1 | 0 | -1.674556 | -1.72368 | 5.14786 |
| 59 | 1 | 0 | 1.877998 | -3.110013 | 0.808497 |
| 60 | 1 | 0 | 3.886258 | 0.790493 | -1.699453 |
| 61 | 1 | 0 | 2.059488 | 7.449642 | -0.737813 |
| 62 | 1 | 0 | 2.856761 | -1.986709 | 6.421851 |
| 63 | 1 | 0 | 5.210368 | -2.867937 | 4.208766 |
| 64 | 1 | 0 | 2.781072 | -5.031978 | 5.033195 |
| 65 | 1 | 0 | -7.466266 | 7.54767 | -0.499955 |
| 66 | 1 | 0 | -6.38327 | 7.284633 | -3.681071 |
| 67 | 1 | 0 | -8.372702 | 4.87237 | -2.361785 |
| 68 | 1 | 0 | 13.516495 | 1.19008 | -1.417 |
| 69 | 1 | 0 | 4.669018 | -3.983293 | -4.816848 |

| Compound**2**-DP4+000007_en_ | | Standard Orientation (A.U.) | | | |
|---|---|---|---|---|---|
| Center number | Atomic number | Atomic Type | X | Y | Z |
| 0 | 8 | 0 | 9.918194 | -3.193303 | 3.006804 |
| 1 | 6 | 0 | 6.766735 | -4.179555 | -1.779689 |
| 2 | 8 | 0 | 13.594197 | -2.127274 | -2.694591 |
| 3 | 6 | 0 | 10.97124 | 0.832952 | 1.178974 |
| 4 | 6 | 0 | 11.611915 | -1.996991 | 1.302375 |
| 5 | 6 | 0 | 11.477684 | -3.17052 | -1.357974 |
| 6 | 6 | 0 | 8.98325 | -2.557474 | -2.702522 |
| 7 | 8 | 0 | 8.459692 | 0.10744 | -2.673911 |
| 8 | 6 | 0 | 8.56645 | 1.385317 | -0.347625 |
| 9 | 1 | 0 | 2.203358 | 1.817849 | 3.713765 |
| 10 | 8 | 0 | -16.189881 | -3.569776 | 1.186003 |
| 11 | 6 | 0 | -15.256345 | -5.004346 | -0.893355 |
| 12 | 6 | 0 | -13.64695 | -3.441637 | -2.737155 |
| 13 | 6 | 0 | -11.276854 | -2.387074 | -1.550764 |
| 14 | 6 | 0 | -8.959537 | -3.624481 | -1.831383 |
| 15 | 6 | 0 | -6.766845 | -2.708945 | -0.671626 |
| 16 | 6 | 0 | -6.902314 | -0.533421 | 0.790703 |
| 17 | 6 | 0 | -9.209741 | 0.747714 | 1.078331 |
| 18 | 6 | 0 | -11.37449 | -0.181863 | -0.083868 |
| 19 | 8 | 0 | -4.928404 | 0.595763 | 2.072822 |
| 20 | 8 | 0 | -9.316675 | 2.910123 | 2.485884 |
| 21 | 6 | 0 | -2.466518 | -0.11537 | 1.54916 |
| 22 | 6 | 0 | -1.302911 | -2.191899 | 3.14602 |
| 23 | 6 | 0 | 1.557544 | -2.052611 | 2.528176 |
| 24 | 6 | 0 | 1.970189 | 0.774608 | 1.931782 |
| 25 | 6 | 0 | -0.501993 | 1.681554 | 0.755208 |
| 26 | 6 | 0 | 4.119422 | 1.536663 | 0.220023 |
| 27 | 8 | 0 | 4.089468 | 4.313712 | 0.004188 |
| 28 | 6 | 0 | 1.852538 | 5.450694 | -0.246488 |
| 29 | 6 | 0 | -0.466558 | 4.366207 | -0.005284 |
| 30 | 8 | 0 | -1.637038 | -0.180168 | -1.002167 |
| 31 | 6 | 0 | 3.225485 | -3.063839 | 4.655779 |
| 32 | 6 | 0 | -2.622673 | 6.000459 | -0.695032 |
| 33 | 8 | 0 | -4.760218 | 4.702064 | -1.155671 |
| 34 | 8 | 0 | -2.484999 | 8.295953 | -0.926448 |
| 35 | 8 | 0 | 6.436284 | 0.875926 | 1.240057 |
| 36 | 6 | 0 | -6.879682 | 6.183499 | -2.001634 |
| 37 | 8 | 0 | 13.020508 | 2.195206 | 0.104281 |

| | | | | | |
|---|---|---|---|---|---|
| 38 | 8 | 0 | 4.471449 | -3.605267 | -3.053748 |
| 39 | 1 | 0 | 10.482948 | -4.922325 | 3.245962 |
| 40 | 1 | 0 | 6.416945 | -3.857666 | 0.217481 |
| 41 | 1 | 0 | 7.285079 | -6.177245 | -2.032066 |
| 42 | 1 | 0 | 13.453329 | -2.574637 | -4.468669 |
| 43 | 1 | 0 | 10.688888 | 1.544186 | 3.097382 |
| 44 | 1 | 0 | 13.569169 | -2.151977 | 1.97085 |
| 45 | 1 | 0 | 11.680618 | -5.23299 | -1.217522 |
| 46 | 1 | 0 | 9.239822 | -2.958179 | -4.722851 |
| 47 | 1 | 0 | 8.561907 | 3.384647 | -0.857571 |
| 48 | 1 | 0 | -17.31386 | -2.291813 | 0.501841 |
| 49 | 1 | 0 | -14.104287 | -6.508387 | -0.062406 |
| 50 | 1 | 0 | -16.816677 | -5.901239 | -1.934664 |
| 51 | 1 | 0 | -13.154231 | -4.658368 | -4.341249 |
| 52 | 1 | 0 | -14.821615 | -1.905931 | -3.493106 |
| 53 | 1 | 0 | -8.846849 | -5.32464 | -2.976716 |
| 54 | 1 | 0 | -4.978005 | -3.668028 | -0.940003 |
| 55 | 1 | 0 | -13.138489 | 0.832126 | 0.169051 |
| 56 | 1 | 0 | -7.584697 | 3.320279 | 2.960084 |
| 57 | 1 | 0 | -2.118035 | -4.04782 | 2.736597 |
| 58 | 1 | 0 | -1.656946 | -1.763218 | 5.142427 |
| 59 | 1 | 0 | 1.897166 | -3.128032 | 0.796886 |
| 60 | 1 | 0 | 3.898535 | 0.7927 | -1.695421 |
| 61 | 1 | 0 | 2.040672 | 7.438187 | -0.711478 |
| 62 | 1 | 0 | 5.23113 | -2.886396 | 4.195561 |
| 63 | 1 | 0 | 2.809496 | -5.061233 | 5.014082 |
| 64 | 1 | 0 | 2.87625 | -2.020584 | 6.413332 |
| 65 | 1 | 0 | -6.377 | 7.244201 | -3.699095 |
| 66 | 1 | 0 | -8.366553 | 4.820022 | -2.401723 |
| 67 | 1 | 0 | -7.484206 | 7.492857 | -0.525166 |
| 68 | 1 | 0 | 13.526714 | 1.238213 | -1.394425 |
| 69 | 1 | 0 | 4.704549 | -3.945605 | -4.841915 |

| Compound**2**-DP4+000008_en_ | | Standard Orientation (A.U.) | | | |
|---|---|---|---|---|---|
| Center number | Atomic number | Atomic Type | X | Y | Z |
| 0 | 8 | 0 | 9.866219 | -3.151099 | 3.051993 |
| 1 | 6 | 0 | 6.725278 | -4.202728 | -1.770662 |
| 2 | 8 | 0 | 13.431194 | -2.246297 | -2.884397 |
| 3 | 6 | 0 | 10.967058 | 0.825899 | 1.144743 |
| 4 | 6 | 0 | 11.573762 | -2.007588 | 1.323086 |

| | | | | | |
|---|---|---|---|---|---|
| 5 | 6 | 0 | 11.428977 | -3.222461 | -1.324422 |
| 6 | 6 | 0 | 8.957514 | -2.602839 | -2.69029 |
| 7 | 8 | 0 | 8.441803 | 0.067278 | -2.681682 |
| 8 | 6 | 0 | 8.554745 | 1.369959 | -0.37121 |
| 9 | 1 | 0 | 2.202916 | 1.850907 | 3.708467 |
| 10 | 8 | 0 | -15.956343 | -3.986545 | 1.275025 |
| 11 | 6 | 0 | -15.110836 | -5.170357 | -0.990398 |
| 12 | 6 | 0 | -13.659999 | -3.373902 | -2.750661 |
| 13 | 6 | 0 | -11.288999 | -2.320126 | -1.565761 |
| 14 | 6 | 0 | -8.968302 | -3.54616 | -1.86974 |
| 15 | 6 | 0 | -6.775694 | -2.637646 | -0.705586 |
| 16 | 6 | 0 | -6.913401 | -0.47884 | 0.782163 |
| 17 | 6 | 0 | -9.223715 | 0.790816 | 1.092685 |
| 18 | 6 | 0 | -11.390099 | -0.135607 | -0.070211 |
| 19 | 8 | 0 | -4.938452 | 0.644153 | 2.067757 |
| 20 | 8 | 0 | -9.332942 | 2.938643 | 2.522242 |
| 21 | 6 | 0 | -2.47745 | -0.07122 | 1.546244 |
| 22 | 6 | 0 | -1.321998 | -2.149955 | 3.145642 |
| 23 | 6 | 0 | 1.539411 | -2.020061 | 2.531477 |
| 24 | 6 | 0 | 1.962749 | 0.804583 | 1.929291 |
| 25 | 6 | 0 | -0.506579 | 1.719452 | 0.752633 |
| 26 | 6 | 0 | 4.113091 | 1.551733 | 0.212082 |
| 27 | 8 | 0 | 4.094868 | 4.328495 | -0.013687 |
| 28 | 6 | 0 | 1.8629 | 5.475283 | -0.262447 |
| 29 | 6 | 0 | -0.460781 | 4.402555 | -0.01288 |
| 30 | 8 | 0 | -1.645925 | -0.140057 | -1.004197 |
| 31 | 6 | 0 | 3.201612 | -3.03093 | 4.663632 |
| 32 | 6 | 0 | -2.611768 | 6.04492 | -0.698496 |
| 33 | 8 | 0 | -4.758465 | 4.754999 | -1.139633 |
| 34 | 8 | 0 | -2.464052 | 8.338747 | -0.940655 |
| 35 | 8 | 0 | 6.429238 | 0.884872 | 1.229996 |
| 36 | 6 | 0 | -6.878728 | 6.242504 | -1.971842 |
| 37 | 8 | 0 | 13.020846 | 2.130145 | 0.005922 |
| 38 | 8 | 0 | 4.438213 | -3.604356 | -3.047764 |
| 39 | 1 | 0 | 10.395593 | -4.88922 | 3.306488 |
| 40 | 1 | 0 | 6.373944 | -3.880838 | 0.226841 |
| 41 | 1 | 0 | 7.224466 | -6.205184 | -2.025896 |
| 42 | 1 | 0 | 15.020743 | -2.892061 | -2.229926 |
| 43 | 1 | 0 | 10.721473 | 1.5907 | 3.04737 |
| 44 | 1 | 0 | 13.526531 | -2.168192 | 2.01355 |
| 45 | 1 | 0 | 11.605239 | -5.283797 | -1.160753 |
| 46 | 1 | 0 | 9.262487 | -3.013767 | -4.696697 |

| | | | | | |
|---|---|---|---|---|---|
| 47 | 1 | 0 | 8.553692 | 3.363911 | -0.902709 |
| 48 | 1 | 0 | -17.14397 | -2.674881 | 0.792092 |
| 49 | 1 | 0 | -13.866999 | -6.709025 | -0.385602 |
| 50 | 1 | 0 | -16.703735 | -6.012466 | -2.028551 |
| 51 | 1 | 0 | -13.189276 | -4.419472 | -4.477776 |
| 52 | 1 | 0 | -14.930006 | -1.831778 | -3.313593 |
| 53 | 1 | 0 | -8.853584 | -5.231578 | -3.03669 |
| 54 | 1 | 0 | -4.98433 | -3.586893 | -0.99095 |
| 55 | 1 | 0 | -13.156589 | 0.871534 | 0.19852 |
| 56 | 1 | 0 | -7.601098 | 3.348226 | 2.997389 |
| 57 | 1 | 0 | -2.142946 | -4.003292 | 2.735836 |
| 58 | 1 | 0 | -1.677542 | -1.718989 | 5.14128 |
| 59 | 1 | 0 | 1.878136 | -3.100228 | 0.803024 |
| 60 | 1 | 0 | 3.88608 | 0.801707 | -1.700262 |
| 61 | 1 | 0 | 2.059284 | 7.460654 | -0.733195 |
| 62 | 1 | 0 | 5.208138 | -2.857908 | 4.205914 |
| 63 | 1 | 0 | 2.780851 | -5.026774 | 5.025143 |
| 64 | 1 | 0 | 2.851313 | -1.983613 | 6.418606 |
| 65 | 1 | 0 | -6.386373 | 7.297319 | -3.675995 |
| 66 | 1 | 0 | -8.374172 | 4.883903 | -2.35653 |
| 67 | 1 | 0 | -7.465817 | 7.557786 | -0.493491 |
| 68 | 1 | 0 | 13.440936 | 1.164729 | -1.514147 |
| 69 | 1 | 0 | 4.682475 | -3.916954 | -4.839547 |

| Compound**2**-DP4+000009_en_ | | Standard Orientation (A.U.) | | | |
|---|---|---|---|---|---|
| Center number | Atomic number | Atomic Type | X | Y | Z |
| 0 | 8 | 0 | -9.363468 | -3.715986 | -2.988507 |
| 1 | 6 | 0 | -9.392759 | -3.993192 | 4.690886 |
| 2 | 8 | 0 | -14.618825 | -2.565889 | 1.392271 |
| 3 | 6 | 0 | -10.642322 | 0.551055 | -1.826384 |
| 4 | 6 | 0 | -11.361549 | -2.259135 | -1.934486 |
| 5 | 6 | 0 | -12.090464 | -3.185906 | 0.74009 |
| 6 | 6 | 0 | -10.207983 | -2.100153 | 2.69679 |
| 7 | 8 | 0 | -7.916163 | -1.296921 | 1.44528 |
| 8 | 6 | 0 | -8.309099 | 0.875969 | -0.094101 |
| 9 | 1 | 0 | -1.908072 | 2.389555 | -3.808133 |
| 10 | 8 | 0 | 17.56478 | -6.175714 | 1.03964 |
| 11 | 6 | 0 | 15.356506 | -4.97021 | 0.081073 |
| 12 | 6 | 0 | 13.899191 | -3.511971 | 2.125379 |
| 13 | 6 | 0 | 11.519379 | -2.33247 | 1.082637 |

| | | | | | |
|---|---|---|---|---|---|
| 14 | 6 | 0 | 9.221002 | -3.634289 | 1.144072 |
| 15 | 6 | 0 | 7.033311 | -2.596225 | 0.083904 |
| 16 | 6 | 0 | 7.155658 | -0.236401 | -1.059314 |
| 17 | 6 | 0 | 9.443415 | 1.10844 | -1.125468 |
| 18 | 6 | 0 | 11.604799 | 0.054883 | -0.06523 |
| 19 | 8 | 0 | 5.183122 | 1.028655 | -2.210091 |
| 20 | 8 | 0 | 9.536802 | 3.447512 | -2.211252 |
| 21 | 6 | 0 | 2.726126 | 0.223893 | -1.833348 |
| 22 | 6 | 0 | 1.609298 | -1.623018 | -3.722755 |
| 23 | 6 | 0 | -1.259113 | -1.603574 | -3.126781 |
| 24 | 6 | 0 | -1.705775 | 1.118412 | -2.177388 |
| 25 | 6 | 0 | 0.726679 | 1.870761 | -0.826625 |
| 26 | 6 | 0 | -3.906483 | 1.609181 | -0.442578 |
| 27 | 8 | 0 | -3.931033 | 4.28797 | 0.236116 |
| 28 | 6 | 0 | -1.709505 | 5.407534 | 0.696158 |
| 29 | 6 | 0 | 0.629014 | 4.410849 | 0.323689 |
| 30 | 8 | 0 | 1.85241 | -0.210472 | 0.667468 |
| 31 | 6 | 0 | -2.901691 | -2.377071 | -5.369436 |
| 32 | 6 | 0 | 2.750678 | 5.946949 | 1.297314 |
| 33 | 8 | 0 | 4.895868 | 4.615184 | 1.583978 |
| 34 | 8 | 0 | 2.572346 | 8.17487 | 1.880977 |
| 35 | 8 | 0 | -6.178994 | 1.117977 | -1.66513 |
| 36 | 6 | 0 | 6.983672 | 5.963979 | 2.692848 |
| 37 | 8 | 0 | -12.792546 | 1.833834 | -0.824731 |
| 38 | 8 | 0 | -7.773557 | -2.888501 | 6.520286 |
| 39 | 1 | 0 | -7.962045 | -3.59835 | -1.801897 |
| 40 | 1 | 0 | -8.486851 | -5.61391 | 3.750143 |
| 41 | 1 | 0 | -11.068028 | -4.683106 | 5.686445 |
| 42 | 1 | 0 | -14.761979 | -0.748621 | 1.116683 |
| 43 | 1 | 0 | -10.157163 | 1.248848 | -3.713142 |
| 44 | 1 | 0 | -12.996291 | -2.500882 | -3.172397 |
| 45 | 1 | 0 | -11.986529 | -5.252766 | 0.719504 |
| 46 | 1 | 0 | -11.067776 | -0.454379 | 3.621583 |
| 47 | 1 | 0 | -8.52322 | 2.542092 | 1.126492 |
| 48 | 1 | 0 | 18.70611 | -4.85293 | 1.59848 |
| 49 | 1 | 0 | 15.817531 | -3.692625 | -1.493846 |
| 50 | 1 | 0 | 14.16516 | -6.482749 | -0.676095 |
| 51 | 1 | 0 | 13.447172 | -4.822014 | 3.663899 |
| 52 | 1 | 0 | 15.139938 | -2.044748 | 2.908096 |
| 53 | 1 | 0 | 9.120731 | -5.478972 | 2.038999 |
| 54 | 1 | 0 | 5.257235 | -3.610078 | 0.181212 |
| 55 | 1 | 0 | 13.353712 | 1.127902 | -0.128919 |

| | | | | | |
|---|---|---|---|---|---|
| 56 | 1 | 0 | 7.807611 | 3.897496 | -2.658847 |
| 57 | 1 | 0 | 2.443 | -3.508399 | -3.566157 |
| 58 | 1 | 0 | 1.9749 | -0.915474 | -5.635524 |
| 59 | 1 | 0 | -1.595658 | -2.899567 | -1.547886 |
| 60 | 1 | 0 | -3.75283 | 0.549744 | 1.327336 |
| 61 | 1 | 0 | -1.944024 | 7.291786 | 1.465974 |
| 62 | 1 | 0 | -2.624979 | -1.080825 | -6.962671 |
| 63 | 1 | 0 | -4.907305 | -2.365926 | -4.876862 |
| 64 | 1 | 0 | -2.399565 | -4.282121 | -6.006561 |
| 65 | 1 | 0 | 8.474577 | 4.564518 | 2.912561 |
| 66 | 1 | 0 | 7.604713 | 7.486762 | 1.445945 |
| 67 | 1 | 0 | 6.435083 | 6.748433 | 4.52086 |
| 68 | 1 | 0 | -12.491305 | 3.642411 | -0.848886 |
| 69 | 1 | 0 | -6.391681 | -2.131014 | 5.57751 |

**Table S5.** Experimental and calculated $^{13}$C chemical shift values of the part structure of **1** and **2** including unit A and the phenylethanoid moiety in unit B

| No. | Exp. chemical shift of (**1**) | Cal. chemical shift of (4a*S*-1) | Exp. chemical shift of (**2**) | Cal. Chemical shift of (5a*S*6a*R*-2) |
|---|---|---|---|---|
| 1a | 97.0 | 98.70 | 101.3 | 103.08 |
| 3a | 88.4 | 91.68 | 160.7 | 173.54 |
| 4a | 76.9 | 80.59 | 109.0 | 108.33 |
| 5a | 51.8 | 56.63 | 79.0 | 73.34 |
| 6a | 216.3 | 230.83 | 103.8 | 99.78 |
| 7a | 48.0 | 52.26 | 46.7 | 43.34 |
| 8a | 33.8 | 37.92 | 30.1 | 33.14 |
| 9a | 46.2 | 48.56 | 53.5 | 52.93 |
| 10a | 19.1 | 20.38 | 21.1 | 20.59 |
| 11a | 170.9 | 180.16 | 168.1 | 175.55 |
| 12a | 53.6 | 56.84 | 52.0 | 55.60 |
| 1'a | 99.2 | 101.74 | 100.8 | 101.14 |
| 2'a | 74.8 | 76.79 | 75.0 | 74.26 |
| 3'a | 78.2 | 77.02 | 78.1 | 73.09 |
| 4'a | 71.6 | 75.06 | 71.6 | 74.46 |
| 5'a | 78.4 | 84.21 | 78.5 | 84.30 |

| | | | | |
|---|---|---|---|---|
| 6'a | 62.7 | 69.09 | 62.8 | 67.08 |
| 1b | 135.1 | 143.88 | 134.1 | 142.04 |
| 2b | 118.0 | 123.00 | 118.0 | 122.46 |
| 3b | 142.4 | 148.29 | 143.7 | 152.54 |
| 4b | 139.2 | 145.00 | 140.3 | 148.00 |
| 5b | 118.1 | 122.97 | 117.8 | 120.03 |
| 6b | 123.7 | 130.97 | 122.6 | 126.38 |
| 7b | 36.5 | 43.33 | 36.5 | 44.43 |
| 8b | 71.7 | 69.42 | 71.9 | 69.66 |

**Table S6.** Cartesian coordinates for the low-energy reoptimized random research conformers of compound **1** (unit A and the phenylethanoid moiety in unit B) (obtained by ECD calculation)

| Compound**1**-ECD1000001_en_ | | Standard Orientation (A.U.) | | | |
|---|---|---|---|---|---|
| Center number | Atomic number | Atomic Type | X | Y | Z |
| 0 | 6 | C | -0.137814 | 8.434154 | 1.991146 |
| 1 | 6 | C | -0.359981 | 5.818508 | 2.186835 |
| 2 | 6 | C | -1.927007 | 4.759151 | 4.026632 |
| 3 | 6 | C | -3.246738 | 6.309072 | 5.69123 |
| 4 | 6 | C | -2.998056 | 8.927263 | 5.508744 |
| 5 | 6 | C | -1.443942 | 10.021346 | 3.657792 |
| 6 | 6 | C | -1.277005 | 12.852562 | 3.382166 |
| 7 | 6 | C | -3.271569 | 13.912128 | 1.560525 |
| 8 | 8 | O | -3.220759 | 16.570477 | 1.400758 |
| 9 | 6 | C | -1.786725 | 0.876064 | 1.921305 |
| 10 | 6 | C | 0.749394 | 1.650241 | 0.66943 |
| 11 | 6 | C | 2.941328 | 0.422804 | 2.095408 |
| 12 | 6 | C | 2.667469 | -2.48258 | 2.243167 |
| 13 | 6 | C | -0.046824 | -3.367515 | 1.747766 |
| 14 | 8 | O | -1.918049 | -1.674456 | 2.589407 |
| 15 | 6 | C | 3.191411 | 1.329154 | 4.861167 |
| 16 | 6 | C | 2.838826 | -0.936248 | 6.586907 |
| 17 | 6 | C | 3.59507 | -3.202812 | 4.941774 |
| 18 | 8 | O | 3.702438 | 3.456479 | 5.483481 |
| 19 | 6 | C | 6.455701 | -3.630051 | 4.966915 |
| 20 | 8 | O | -0.374228 | -3.848879 | -0.860891 |
| 21 | 6 | C | -2.418271 | -5.522022 | -1.429523 |
| 22 | 6 | C | -3.186978 | -4.996823 | -4.172776 |
| 23 | 6 | C | -1.186448 | -6.067228 | -5.99156 |

| | | | | | |
|---|---|---|---|---|---|
| 24 | 6 | C | -0.797056 | -8.896763 | -5.416172 |
| 25 | 6 | C | -0.113629 | -9.337012 | -2.646822 |
| 26 | 8 | O | -1.791281 | -8.04 | -0.946227 |
| 27 | 6 | C | 2.716236 | -8.770499 | -2.038839 |
| 28 | 8 | O | 4.320547 | -8.772489 | -4.154038 |
| 29 | 8 | O | -5.593659 | -5.980161 | -4.684778 |
| 30 | 8 | O | 1.162 | -4.765578 | -5.8919 |
| 31 | 8 | O | -3.064198 | -10.280216 | -5.919665 |
| 32 | 8 | O | -2.06723 | 2.17938 | 4.23293 |
| 33 | 8 | O | 1.005735 | 4.333352 | 0.581975 |
| 34 | 6 | C | 0.91853 | 0.97878 | -2.164447 |
| 35 | 8 | O | -1.161838 | 1.670909 | -3.400515 |
| 36 | 8 | O | 2.773404 | 0.096106 | -3.152169 |
| 37 | 6 | C | -1.052681 | 1.551951 | -6.108844 |
| 38 | 1 | H | 4.683265 | 0.965958 | 1.106537 |
| 39 | 1 | H | 3.852224 | -3.398187 | 0.80939 |
| 40 | 1 | H | -3.375026 | 1.364964 | 0.662045 |
| 41 | 1 | H | 1.1148 | 9.200997 | 0.541887 |
| 42 | 1 | H | -4.438532 | 5.427962 | 7.12526 |
| 43 | 1 | H | -4.021521 | 10.142088 | 6.827354 |
| 44 | 1 | H | -1.528658 | 13.785012 | 5.225973 |
| 45 | 1 | H | 0.616489 | 13.385263 | 2.684294 |
| 46 | 1 | H | -3.044613 | 13.003789 | -0.315371 |
| 47 | 1 | H | -5.169593 | 13.39649 | 2.243625 |
| 48 | 1 | H | -1.595158 | 17.067521 | 0.734703 |
| 49 | 1 | H | -0.435011 | -5.119041 | 2.796721 |
| 50 | 1 | H | 0.81047 | -0.998766 | 7.062215 |
| 51 | 1 | H | 3.917822 | -0.722062 | 8.347576 |
| 52 | 1 | H | 2.648801 | -4.944733 | 5.574318 |
| 53 | 1 | H | 7.124698 | -4.074782 | 6.885903 |
| 54 | 1 | H | 6.994377 | -5.200708 | 3.712858 |
| 55 | 1 | H | 7.479768 | -1.93102 | 4.328121 |
| 56 | 1 | H | -4.02695 | -5.0976 | -0.18395 |
| 57 | 1 | H | -3.301507 | -2.932426 | -4.385988 |
| 58 | 1 | H | -1.933024 | -5.878033 | -7.930532 |
| 59 | 1 | H | 0.774015 | -9.632435 | -6.562771 |
| 60 | 1 | H | -0.508841 | -11.343629 | -2.28689 |
| 61 | 1 | H | 3.412609 | -10.243038 | -0.745471 |
| 62 | 1 | H | 2.802309 | -6.968405 | -1.007679 |
| 63 | 1 | H | 3.897173 | -7.270101 | -5.129199 |
| 64 | 1 | H | -5.402079 | -7.807878 | -4.801401 |
| 65 | 1 | H | 1.296301 | -3.78004 | -4.344596 |

| | | | | | |
|---|---|---|---|---|---|
| 66 | 1 | H | -3.301962 | -10.359501 | -7.72926 |
| 67 | 1 | H | -2.98891 | 1.86766 | -6.772406 |
| 68 | 1 | H | 0.205761 | 3.04528 | -6.821821 |
| 69 | 1 | H | -0.335767 | -0.291664 | -6.734761 |

**Table S7.** Cartesian coordinates for the low-energy reoptimized random research conformers of compound **2** (unit A and the phenylethanoid moiety in unit B) (obtained by ECD calculation)

| Compound**2**-ECD2000001_en_ | | Standard Orientation (A.U.) | | | |
|---|---|---|---|---|---|
| Center number | Atomic number | Atomic Type | X | Y | Z |
| 0 | 6 | C | 8.533487 | -4.853251 | -1.231623 |
| 1 | 6 | C | 6.161358 | -4.489328 | -0.112397 |
| 2 | 6 | C | 5.975501 | -2.778221 | 1.918839 |
| 3 | 6 | C | 8.101139 | -1.492464 | 2.784695 |
| 4 | 6 | C | 10.447351 | -1.877636 | 1.646048 |
| 5 | 6 | C | 10.681622 | -3.565428 | -0.38547 |
| 6 | 6 | C | 13.224921 | -4.084222 | -1.563486 |
| 7 | 6 | C | 14.580114 | -6.293833 | -0.295844 |
| 8 | 8 | O | 16.932322 | -6.633681 | -1.507372 |
| 9 | 8 | O | 3.664251 | -2.46733 | 3.130904 |
| 10 | 8 | O | 4.17436 | -5.811091 | -0.992783 |
| 11 | 6 | C | 1.970011 | -0.884842 | 1.984031 |
| 12 | 6 | C | 2.561471 | 1.907257 | 1.718997 |
| 13 | 6 | C | 0.027603 | 3.098595 | 0.867265 |
| 14 | 6 | C | -1.952301 | 1.384415 | 2.132454 |
| 15 | 6 | C | -0.778546 | -1.245955 | 2.142966 |
| 16 | 8 | O | 0.627419 | -1.814721 | -0.174505 |
| 17 | 6 | C | -4.586535 | 1.176381 | 1.061769 |
| 18 | 8 | O | -6.032868 | -0.474408 | 2.728067 |
| 19 | 6 | C | -4.924836 | -2.600144 | 3.506978 |
| 20 | 6 | C | -2.441985 | -3.206565 | 3.23085 |
| 21 | 6 | C | -1.72261 | -5.810401 | 3.96814 |
| 22 | 8 | O | 0.383569 | -6.601031 | 2.781641 |
| 23 | 8 | O | -2.921405 | -7.141145 | 5.390774 |
| 24 | 8 | O | -5.780532 | 3.482954 | 0.985832 |
| 25 | 6 | C | -8.314166 | 3.500009 | 0.102092 |
| 26 | 6 | C | -9.178663 | 6.259637 | 0.178107 |
| 27 | 6 | C | -8.184803 | 7.787966 | -2.088858 |
| 28 | 6 | C | -8.88588 | 6.408078 | -4.564246 |

| | | | | | |
|---|---|---|---|---|---|
| 29 | 6 | C | -8.113962 | 3.58839 | -4.567381 |
| 30 | 8 | O | -8.637175 | 2.315224 | -2.229656 |
| 31 | 8 | O | -5.613825 | 8.366562 | -1.868314 |
| 32 | 8 | O | -11.491086 | 6.643198 | -5.06834 |
| 33 | 6 | C | -5.484108 | 2.984282 | -5.562443 |
| 34 | 1 | H | -2.167694 | 1.960916 | 4.128934 |
| 35 | 6 | C | -0.236038 | 5.895496 | 1.502174 |
| 36 | 6 | C | 1.298357 | -9.056216 | 3.427778 |
| 37 | 8 | O | -3.56987 | 4.202847 | -4.125444 |
| 38 | 8 | O | -11.865048 | 6.372351 | 0.063657 |
| 39 | 1 | H | 8.650892 | -6.181759 | -2.807336 |
| 40 | 1 | H | 7.880999 | -0.198741 | 4.376708 |
| 41 | 1 | H | 12.106007 | -0.858882 | 2.333547 |
| 42 | 1 | H | 13.02315 | -4.52647 | -3.587605 |
| 43 | 1 | H | 14.452888 | -2.408945 | -1.42461 |
| 44 | 1 | H | 14.804237 | -5.875691 | 1.74612 |
| 45 | 1 | H | 13.374382 | -8.002905 | -0.433395 |
| 46 | 1 | H | 17.791161 | -8.035773 | -0.7196 |
| 47 | 1 | H | 2.609292 | -5.222946 | -0.211647 |
| 48 | 1 | H | 4.110022 | 2.249254 | 0.375266 |
| 49 | 1 | H | 3.147108 | 2.642527 | 3.57892 |
| 50 | 1 | H | -0.146174 | 2.826879 | -1.182696 |
| 51 | 1 | H | -4.583476 | 0.24945 | -0.806025 |
| 52 | 1 | H | -6.236718 | -3.911246 | 4.414568 |
| 53 | 1 | H | -9.463692 | 2.358002 | 1.415331 |
| 54 | 1 | H | -8.446033 | 7.111096 | 1.933335 |
| 55 | 1 | H | -9.235538 | 9.584476 | -2.103698 |
| 56 | 1 | H | -7.874871 | 7.351333 | -6.122682 |
| 57 | 1 | H | -9.417894 | 2.699532 | -5.923142 |
| 58 | 1 | H | -4.619829 | 6.933571 | -2.467691 |
| 59 | 1 | H | -12.363654 | 6.353116 | -3.476378 |
| 60 | 1 | H | -5.41436 | 3.595142 | -7.558423 |
| 61 | 1 | H | -5.224518 | 0.913263 | -5.510082 |
| 62 | 1 | H | -0.066647 | 6.203434 | 3.556402 |
| 63 | 1 | H | -2.077346 | 6.639218 | 0.901084 |
| 64 | 1 | H | 1.259221 | 7.009563 | 0.575588 |
| 65 | 1 | H | 2.953266 | -9.362336 | 2.221908 |
| 66 | 1 | H | 1.826426 | -9.126143 | 5.437056 |
| 67 | 1 | H | -0.155262 | -10.497616 | 3.07095 |
| 68 | 1 | H | -1.970848 | 4.088923 | -4.996532 |
| 69 | 1 | H | -12.579943 | 5.329426 | 1.381301 |

| Compound**2**-ECD2000002_en_ | | Standard Orientation (A.U.) | | | |
|---|---|---|---|---|---|
| Center number | Atomic number | Atomic Type | X | Y | Z |
| 0 | 6 | C | 8.517122 | -4.892633 | -1.092914 |
| 1 | 6 | C | 6.147731 | -4.50653 | 0.025596 |
| 2 | 6 | C | 5.974872 | -2.781533 | 2.04629 |
| 3 | 6 | C | 8.110852 | -1.50528 | 2.901913 |
| 4 | 6 | C | 10.453806 | -1.91258 | 1.765163 |
| 5 | 6 | C | 10.674339 | -3.613525 | -0.257415 |
| 6 | 6 | C | 13.218277 | -4.167226 | -1.416359 |
| 7 | 6 | C | 14.595572 | -6.337858 | -0.069117 |
| 8 | 8 | O | 17.031535 | -6.785011 | -1.050314 |
| 9 | 8 | O | 3.669629 | -2.448866 | 3.261127 |
| 10 | 8 | O | 4.153716 | -5.822002 | -0.845649 |
| 11 | 6 | C | 1.977585 | -0.868779 | 2.106636 |
| 12 | 6 | C | 2.577507 | 1.92024 | 1.829933 |
| 13 | 6 | C | 0.048422 | 3.114639 | 0.968428 |
| 14 | 6 | C | -1.939881 | 1.410924 | 2.234427 |
| 15 | 6 | C | -0.771696 | -1.221806 | 2.264963 |
| 16 | 8 | O | 0.636639 | -1.805545 | -0.050497 |
| 17 | 6 | C | -4.571181 | 1.197831 | 1.153852 |
| 18 | 8 | O | -6.024058 | -0.431228 | 2.833758 |
| 19 | 6 | C | -4.92512 | -2.550934 | 3.632261 |
| 20 | 6 | C | -2.443176 | -3.169645 | 3.362431 |
| 21 | 6 | C | -1.732191 | -5.768603 | 4.120153 |
| 22 | 8 | O | 0.377412 | -6.572324 | 2.944987 |
| 23 | 8 | O | -2.936181 | -7.088631 | 5.547971 |
| 24 | 8 | O | -5.756206 | 3.504516 | 1.033317 |
| 25 | 6 | C | -8.304995 | 3.516894 | 0.184607 |
| 26 | 6 | C | -9.200736 | 6.266033 | 0.211285 |
| 27 | 6 | C | -8.135844 | 7.763526 | -2.05211 |
| 28 | 6 | C | -8.779273 | 6.357226 | -4.527474 |
| 29 | 6 | C | -8.0798 | 3.53537 | -4.491225 |
| 30 | 8 | O | -8.63123 | 2.305931 | -2.15539 |
| 31 | 8 | O | -5.566047 | 8.356055 | -1.855464 |
| 32 | 8 | O | -11.441122 | 6.425046 | -4.981407 |
| 33 | 6 | C | -5.451389 | 2.902772 | -5.478227 |
| 34 | 1 | H | -2.163391 | 2.000319 | 4.225987 |
| 35 | 6 | C | -0.207137 | 5.914569 | 1.59382 |

| | | | | | |
|---|---|---|---|---|---|
| 36 | 6 | C | 1.286701 | -9.022033 | 3.618056 |
| 37 | 8 | O | -3.539948 | 4.147656 | -4.070561 |
| 38 | 8 | O | -11.861614 | 6.351256 | 0.259601 |
| 39 | 1 | H | 8.622072 | -6.234397 | -2.658442 |
| 40 | 1 | H | 7.900756 | -0.201403 | 4.486941 |
| 41 | 1 | H | 12.119734 | -0.902454 | 2.447732 |
| 42 | 1 | H | 12.988471 | -4.668149 | -3.429205 |
| 43 | 1 | H | 14.443258 | -2.48564 | -1.343408 |
| 44 | 1 | H | 14.846271 | -5.850496 | 1.939345 |
| 45 | 1 | H | 13.394764 | -8.05471 | -0.121993 |
| 46 | 1 | H | 16.852704 | -7.327715 | -2.784215 |
| 47 | 1 | H | 2.592574 | -5.225697 | -0.061649 |
| 48 | 1 | H | 4.130321 | 2.252036 | 0.488311 |
| 49 | 1 | H | 3.160951 | 2.662276 | 3.687789 |
| 50 | 1 | H | -0.118297 | 2.834941 | -1.081286 |
| 51 | 1 | H | -4.557623 | 0.239566 | -0.699386 |
| 52 | 1 | H | -6.242145 | -3.84859 | 4.55191 |
| 53 | 1 | H | -9.466553 | 2.407547 | 1.499944 |
| 54 | 1 | H | -8.497429 | 7.131647 | 1.963877 |
| 55 | 1 | H | -9.171215 | 9.576229 | -2.083176 |
| 56 | 1 | H | -7.747082 | 7.276872 | -6.093841 |
| 57 | 1 | H | -9.384875 | 2.649659 | -5.85063 |
| 58 | 1 | H | -4.568386 | 6.887328 | -2.350931 |
| 59 | 1 | H | -11.968021 | 8.174496 | -5.043532 |
| 60 | 1 | H | -5.377648 | 3.462182 | -7.490243 |
| 61 | 1 | H | -5.213412 | 0.831634 | -5.373688 |
| 62 | 1 | H | -0.032433 | 6.228154 | 3.646533 |
| 63 | 1 | H | -2.049089 | 6.661251 | 0.998768 |
| 64 | 1 | H | 1.288145 | 7.022804 | 0.660138 |
| 65 | 1 | H | 1.814279 | -9.071791 | 5.628079 |
| 66 | 1 | H | -0.169045 | -10.464786 | 3.275791 |
| 67 | 1 | H | 2.94223 | -9.345161 | 2.417343 |
| 68 | 1 | H | -1.918057 | 3.916966 | -4.87347 |
| 69 | 1 | H | -12.438051 | 5.830504 | -1.404288 |

| Compound**2**-ECD2000003_en_ | | Standard Orientation (A.U.) | | | |
|---|---|---|---|---|---|
| Center number | Atomic number | Atomic Type | X | Y | Z |
| 0 | 6 | C | 9.484503 | -5.058022 | -0.031798 |
| 1 | 6 | C | 6.8848 | -4.688271 | 0.306854 |
| 2 | 6 | C | 6.091009 | -2.884932 | 2.096825 |
| 3 | 6 | C | 7.85075 | -1.513979 | 3.490211 |
| 4 | 6 | C | 10.432148 | -1.90454 | 3.125138 |
| 5 | 6 | C | 11.27295 | -3.68328 | 1.346968 |
| 6 | 6 | C | 14.052285 | -4.22182 | 1.020248 |
| 7 | 6 | C | 14.973657 | -6.356908 | 2.757201 |
| 8 | 8 | O | 17.538064 | -6.967014 | 2.376952 |
| 9 | 8 | O | 3.518271 | -2.56748 | 2.540814 |
| 10 | 8 | O | 5.258102 | -6.089234 | -1.054953 |
| 11 | 6 | C | 2.243872 | -1.050263 | 0.881967 |
| 12 | 6 | C | 2.876175 | 1.737359 | 0.705525 |
| 13 | 6 | C | 0.709205 | 2.85753 | -0.907705 |
| 14 | 6 | C | -1.56358 | 1.184783 | -0.170843 |
| 15 | 6 | C | -0.425587 | -1.446257 | 0.223301 |
| 16 | 8 | O | 1.604379 | -2.062031 | -1.543757 |
| 17 | 6 | C | -3.786231 | 0.974828 | -1.964933 |
| 18 | 8 | O | -5.59279 | -0.814969 | -0.955172 |
| 19 | 6 | C | -4.756048 | -2.874332 | 0.23898 |
| 20 | 6 | C | -2.316164 | -3.407208 | 0.825533 |
| 21 | 6 | C | -1.841502 | -5.961589 | 1.877269 |
| 22 | 8 | O | 0.525361 | -6.768712 | 1.425451 |
| 23 | 8 | O | -3.415697 | -7.242445 | 2.930321 |
| 24 | 8 | O | -5.123023 | 3.167986 | -2.538605 |
| 25 | 6 | C | -6.003622 | 4.756172 | -0.503533 |
| 26 | 6 | C | -6.349274 | 7.475552 | -1.485287 |
| 27 | 6 | C | -8.904365 | 7.951282 | -2.826543 |
| 28 | 6 | C | -11.049319 | 6.792636 | -1.233768 |
| 29 | 6 | C | -10.555918 | 4.035542 | -0.461067 |
| 30 | 8 | O | -8.127818 | 3.763577 | 0.697044 |
| 31 | 8 | O | -8.964523 | 7.236153 | -5.362631 |
| 32 | 8 | O | -11.208616 | 8.341947 | 0.970677 |
| 33 | 6 | C | -11.068081 | 2.02246 | -2.478136 |
| 34 | 1 | H | -2.27132 | 1.756688 | 1.706712 |
| 35 | 6 | C | 0.373578 | 5.697466 | -0.567235 |
| 36 | 6 | C | 1.205184 | -9.181217 | 2.432561 |
| 37 | 8 | O | -9.65517 | 2.270922 | -4.728029 |

| | | | | | |
|---|---|---|---|---|---|
| 38 | 8 | O | -6.045795 | 9.123832 | 0.58745 |
| 39 | 1 | H | 10.074585 | -6.460841 | -1.426397 |
| 40 | 1 | H | 7.157941 | -0.15094 | 4.875397 |
| 41 | 1 | H | 11.801009 | -0.818146 | 4.224369 |
| 42 | 1 | H | 14.473558 | -4.764361 | -0.944457 |
| 43 | 1 | H | 15.159135 | -2.50058 | 1.429725 |
| 44 | 1 | H | 14.568883 | -5.848597 | 4.750084 |
| 45 | 1 | H | 13.887013 | -8.086172 | 2.353486 |
| 46 | 1 | H | 18.540929 | -5.508858 | 2.825209 |
| 47 | 1 | H | 3.525808 | -5.517802 | -0.78242 |
| 48 | 1 | H | 4.750501 | 2.060404 | -0.131567 |
| 49 | 1 | H | 2.873827 | 2.549581 | 2.624867 |
| 50 | 1 | H | 1.147457 | 2.457232 | -2.901867 |
| 51 | 1 | H | -3.134099 | 0.227349 | -3.799591 |
| 52 | 1 | H | -6.272969 | -4.195152 | 0.701359 |
| 53 | 1 | H | -4.545876 | 4.827498 | 0.962194 |
| 54 | 1 | H | -4.838425 | 7.860777 | -2.858131 |
| 55 | 1 | H | -9.176126 | 10.014107 | -2.760952 |
| 56 | 1 | H | -12.820739 | 6.885334 | -2.337688 |
| 57 | 1 | H | -11.889677 | 3.588061 | 1.083756 |
| 58 | 1 | H | -9.300733 | 5.418962 | -5.484611 |
| 59 | 1 | H | -12.300074 | 7.544777 | 2.198643 |
| 60 | 1 | H | -13.079014 | 2.12485 | -3.00763 |
| 61 | 1 | H | -10.727129 | 0.160959 | -1.601715 |
| 62 | 1 | H | 2.164695 | 6.68327 | -0.95365 |
| 63 | 1 | H | -0.204077 | 6.182135 | 1.373961 |
| 64 | 1 | H | -1.049662 | 6.464286 | -1.867168 |
| 65 | 1 | H | 3.143384 | -9.520276 | 1.788837 |
| 66 | 1 | H | 1.111994 | -9.148622 | 4.508643 |
| 67 | 1 | H | -0.078403 | -10.655189 | 1.728515 |
| 68 | 1 | H | -7.867829 | 2.179684 | -4.268683 |
| 69 | 1 | H | -7.664601 | 9.153824 | 1.459341 |

| Compound**2**-ECD2000004_en_ | | Standard Orientation (A.U.) | | | |
|---|---|---|---|---|---|
| Center number | Atomic number | Atomic Type | X | Y | Z |
| 0 | 6 | C | 9.77109 | -6.003788 | 1.466993 |
| 1 | 6 | C | 7.803894 | -4.763945 | 2.697208 |
| 2 | 6 | C | 6.153111 | -3.211788 | 1.302464 |
| 3 | 6 | C | 6.45822 | -2.934835 | -1.292318 |
| 4 | 6 | C | 8.447966 | -4.192027 | -2.504441 |
| 5 | 6 | C | 10.128885 | -5.725296 | -1.146996 |
| 6 | 6 | C | 12.152972 | -7.208602 | -2.500166 |
| 7 | 6 | C | 11.149834 | -9.752487 | -3.485254 |
| 8 | 8 | O | 9.153869 | -9.475463 | -5.222031 |
| 9 | 8 | O | 4.324206 | -2.08734 | 2.771376 |
| 10 | 8 | O | 7.445292 | -5.049991 | 5.217831 |
| 11 | 6 | C | 2.449319 | -0.674793 | 1.678419 |
| 12 | 6 | C | 2.96118 | 2.114178 | 1.271512 |
| 13 | 6 | C | 0.373355 | 3.22074 | 0.503219 |
| 14 | 6 | C | -1.545669 | 1.522375 | 1.904957 |
| 15 | 6 | C | -0.280003 | -1.074806 | 2.092713 |
| 16 | 8 | O | 0.992938 | -1.764666 | -0.266916 |
| 17 | 6 | C | -4.133273 | 1.148313 | 0.753534 |
| 18 | 8 | O | -5.567269 | -0.564346 | 2.333247 |
| 19 | 6 | C | -4.39854 | -2.549944 | 3.350356 |
| 20 | 6 | C | -1.869368 | -3.0119 | 3.339953 |
| 21 | 6 | C | -1.135773 | -5.47832 | 4.462225 |
| 22 | 8 | O | 1.365779 | -5.885775 | 4.523337 |
| 23 | 8 | O | -2.652319 | -6.999861 | 5.259593 |
| 24 | 8 | O | -5.521052 | 3.364083 | 0.619637 |
| 25 | 6 | C | -7.886014 | 3.163636 | -0.736207 |
| 26 | 6 | C | -9.611642 | 5.245974 | 0.319402 |
| 27 | 6 | C | -8.733447 | 7.829258 | -0.676214 |
| 28 | 6 | C | -8.653239 | 7.768572 | -3.583051 |
| 29 | 6 | C | -6.991437 | 5.602106 | -4.559943 |
| 30 | 8 | O | -7.543029 | 3.238412 | -3.345218 |
| 31 | 8 | O | -6.31295 | 8.495071 | 0.291566 |
| 32 | 8 | O | -11.211537 | 7.498297 | -4.390367 |
| 33 | 6 | C | -4.102325 | 6.195611 | -4.524239 |
| 34 | 1 | H | -1.825661 | 2.22701 | 3.85027 |
| 35 | 6 | C | 0.146781 | 6.039882 | 1.050206 |
| 36 | 6 | C | 2.137029 | -8.298545 | 5.471483 |
| 37 | 8 | O | -3.470809 | 8.736427 | -4.098742 |

| | | | | | |
|---|---|---|---|---|---|
| 38 | 8 | O | -12.145612 | 4.748043 | -0.242427 |
| 39 | 1 | H | 11.025348 | -7.197549 | 2.590191 |
| 40 | 1 | H | 5.145208 | -1.780256 | -2.378377 |
| 41 | 1 | H | 8.696147 | -3.961482 | -4.540014 |
| 42 | 1 | H | 12.872676 | -6.115622 | -4.119667 |
| 43 | 1 | H | 13.759644 | -7.570553 | -1.224777 |
| 44 | 1 | H | 12.673526 | -10.758302 | -4.487185 |
| 45 | 1 | H | 10.593576 | -10.943801 | -1.851461 |
| 46 | 1 | H | 7.79776 | -8.591931 | -4.369525 |
| 47 | 1 | H | 5.940628 | -4.091703 | 5.654979 |
| 48 | 1 | H | 4.43353 | 2.445449 | -0.157809 |
| 49 | 1 | H | 3.626235 | 2.927511 | 3.071156 |
| 50 | 1 | H | 0.13295 | 2.89266 | -1.536945 |
| 51 | 1 | H | -3.984457 | 0.256751 | -1.130447 |
| 52 | 1 | H | -5.701597 | -3.869381 | 4.25538 |
| 53 | 1 | H | -8.735733 | 1.315605 | -0.332985 |
| 54 | 1 | H | -9.401692 | 5.210258 | 2.391229 |
| 55 | 1 | H | -10.113028 | 9.271951 | -0.089065 |
| 56 | 1 | H | -7.855102 | 9.557588 | -4.286058 |
| 57 | 1 | H | -7.563342 | 5.263426 | -6.534087 |
| 58 | 1 | H | -5.546991 | 6.994372 | 1.024007 |
| 59 | 1 | H | -11.258962 | 7.36033 | -6.210031 |
| 60 | 1 | H | -3.295573 | 5.679292 | -6.369957 |
| 61 | 1 | H | -3.236704 | 4.925649 | -3.12251 |
| 62 | 1 | H | 0.21339 | 6.416289 | 3.097563 |
| 63 | 1 | H | -1.610239 | 6.834582 | 0.296893 |
| 64 | 1 | H | 1.707626 | 7.084842 | 0.156534 |
| 65 | 1 | H | 4.205175 | -8.248857 | 5.522626 |
| 66 | 1 | H | 1.358176 | -8.622321 | 7.37038 |
| 67 | 1 | H | 1.465335 | -9.816543 | 4.220002 |
| 68 | 1 | H | -4.100849 | 9.169231 | -2.420675 |
| 69 | 1 | H | -12.43703 | 5.317759 | -1.968055 |

| Compound**2**-ECD2000005_en_ | | Standard Orientation (A.U.) | | | |
|---|---|---|---|---|---|
| Center number | Atomic number | Atomic Type | X | Y | Z |
| 0 | 6 | C | 8.479995 | -4.891424 | -1.119905 |
| 1 | 6 | C | 6.109674 | -4.525236 | 0.002693 |
| 2 | 6 | C | 5.93031 | -2.820617 | 2.040216 |
| 3 | 6 | C | 8.060846 | -1.54405 | 2.908009 |
| 4 | 6 | C | 10.405142 | -1.93167 | 1.766217 |
| 5 | 6 | C | 10.63278 | -3.613045 | -0.271361 |
| 6 | 6 | C | 13.173494 | -4.135968 | -1.453124 |
| 7 | 6 | C | 14.522676 | -6.353783 | -0.193604 |
| 8 | 8 | O | 16.872594 | -6.69818 | -1.408606 |
| 9 | 8 | O | 3.622686 | -2.506724 | 3.257155 |
| 10 | 8 | O | 4.119303 | -5.83956 | -0.880535 |
| 11 | 6 | C | 1.927876 | -0.920941 | 2.115444 |
| 12 | 6 | C | 2.521707 | 1.871515 | 1.860632 |
| 13 | 6 | C | -0.009632 | 3.06726 | 1.007776 |
| 14 | 6 | C | -1.994802 | 1.349162 | 2.259226 |
| 15 | 6 | C | -0.820761 | -1.281055 | 2.269697 |
| 16 | 8 | O | 0.58895 | -1.843052 | -0.049792 |
| 17 | 6 | C | -4.62478 | 1.137094 | 1.175327 |
| 18 | 8 | O | -6.07492 | -0.505889 | 2.844198 |
| 19 | 6 | C | -4.971696 | -2.629715 | 3.626216 |
| 20 | 6 | C | -2.488514 | -3.240999 | 3.351395 |
| 21 | 6 | C | -1.771569 | -5.844961 | 4.086117 |
| 22 | 8 | O | 0.337099 | -6.633865 | 2.899943 |
| 23 | 8 | O | -2.970449 | -7.179638 | 5.504768 |
| 24 | 8 | O | -5.814973 | 3.44165 | 1.067644 |
| 25 | 6 | C | -8.36283 | 3.453983 | 0.216217 |
| 26 | 6 | C | -9.262124 | 6.201828 | 0.253828 |
| 27 | 6 | C | -8.195548 | 7.710389 | -2.001458 |
| 28 | 6 | C | -8.834307 | 6.314509 | -4.484006 |
| 29 | 6 | C | -8.132731 | 3.493074 | -4.459431 |
| 30 | 8 | O | -8.685189 | 2.253005 | -2.129368 |
| 31 | 8 | O | -5.626686 | 8.30457 | -1.798432 |
| 32 | 8 | O | -11.495718 | 6.382437 | -4.941177 |
| 33 | 6 | C | -5.502926 | 2.867213 | -5.44695 |
| 34 | 1 | H | -2.221059 | 1.922866 | 4.255076 |
| 35 | 6 | C | -0.271495 | 5.861541 | 1.655555 |
| 36 | 6 | C | 1.252072 | -9.088065 | 3.546714 |
| 37 | 8 | O | -3.594321 | 4.109691 | -4.033551 |

| | | | | | |
|---|---|---|---|---|---|
| 38 | 8 | O | -11.923197 | 6.283647 | 0.298535 |
| 39 | 1 | H | 8.592095 | -6.215385 | -2.699867 |
| 40 | 1 | H | 7.845438 | -0.255391 | 4.50478 |
| 41 | 1 | H | 12.067232 | -0.920157 | 2.456136 |
| 42 | 1 | H | 12.968877 | -4.571314 | -3.478496 |
| 43 | 1 | H | 14.406822 | -2.464932 | -1.310223 |
| 44 | 1 | H | 14.750378 | -5.942656 | 1.84935 |
| 45 | 1 | H | 13.311063 | -8.058359 | -0.334946 |
| 46 | 1 | H | 17.727795 | -8.104744 | -0.624861 |
| 47 | 1 | H | 2.555637 | -5.249219 | -0.097565 |
| 48 | 1 | H | 4.074095 | 2.216838 | 0.522016 |
| 49 | 1 | H | 3.103323 | 2.60021 | 3.724359 |
| 50 | 1 | H | -0.174713 | 2.803813 | -1.044149 |
| 51 | 1 | H | -4.607385 | 0.190046 | -0.68359 |
| 52 | 1 | H | -6.286161 | -3.937363 | 4.535311 |
| 53 | 1 | H | -9.524171 | 2.337217 | 1.525508 |
| 54 | 1 | H | -8.562411 | 7.06061 | 2.01122 |
| 55 | 1 | H | -9.232769 | 9.522164 | -2.026132 |
| 56 | 1 | H | -7.80081 | 7.24203 | -6.04482 |
| 57 | 1 | H | -9.435977 | 2.612238 | -5.82372 |
| 58 | 1 | H | -4.626804 | 6.839276 | -2.299657 |
| 59 | 1 | H | -12.023638 | 8.131801 | -4.996551 |
| 60 | 1 | H | -5.428516 | 3.433905 | -7.456914 |
| 61 | 1 | H | -5.262392 | 0.796024 | -5.349801 |
| 62 | 1 | H | -0.100558 | 6.158468 | 3.711074 |
| 63 | 1 | H | -2.113772 | 6.609878 | 1.063421 |
| 64 | 1 | H | 1.223265 | 6.979998 | 0.733331 |
| 65 | 1 | H | 2.905817 | -9.395633 | 2.339436 |
| 66 | 1 | H | 1.78338 | -9.157195 | 5.55526 |
| 67 | 1 | H | -0.201553 | -10.530217 | 3.192717 |
| 68 | 1 | H | -1.970761 | 3.880161 | -4.833523 |
| 69 | 1 | H | -12.496375 | 5.770683 | -1.368908 |
